# Climate warming from managed grasslands cancels the cooling effect of carbon sinks in sparsely grazed and natural grasslands

**DOI:** 10.1038/s41467-020-20406-7

**Published:** 2021-01-05

**Authors:** Jinfeng Chang, Philippe Ciais, Thomas Gasser, Pete Smith, Mario Herrero, Petr Havlík, Michael Obersteiner, Bertrand Guenet, Daniel S. Goll, Wei Li, Victoria Naipal, Shushi Peng, Chunjing Qiu, Hanqin Tian, Nicolas Viovy, Chao Yue, Dan Zhu

**Affiliations:** 1 Laboratoire des Sciences du Climat et de l’Environnement, LSCE/IPSL, CEA-CNRS-UVSQ, Université Paris-Saclay, 91191 Gif-sur-Yvette, France; 2 International Institute for Applied Systems Analysis, A-2361 Laxenburg, Austria; 3 College of Environmental and Resource Sciences, Zhejiang University, 310058 Hangzhou, China; 4 Institute of Biological & Environmental Sciences, University of Aberdeen, 23 St Machar Drive, Aberdeen, AB24 3UU UK; 5 Commonwealth Scientific and Industrial Research Organization, St Lucia, QLD 4067 Australia; 6 Ludwig-Maximilian University, Munich, Germany; 7 Sino-French Institute for Earth System Science, College of Urban and Environmental Sciences, Peking University, 100871 Beijing, China; 8 International Center for Climate and Global Change Research and School of Forestry and Wildlife Sciences, Auburn University, Auburn, AL USA; 9 State Key Laboratory of Soil Erosion and Dryland Farming on the Loess Plateau, Northwest A&F University, 712100 Yangling, Shaanxi China

**Keywords:** Carbon cycle, Attribution

## Abstract

Grasslands absorb and release carbon dioxide (CO_2_), emit methane (CH_4_) from grazing livestock, and emit nitrous oxide (N_2_O) from soils. Little is known about how the fluxes of these three greenhouse gases, from managed and natural grasslands worldwide, have contributed to past climate change, or the roles of managed pastures versus natural grasslands. Here, global trends and regional patterns of the full greenhouse gas balance of grasslands are estimated for the period 1750 to 2012. A new spatially explicit land surface model is applied, to separate the direct effects of human activities from land management and the indirect effects from climate change, increasing CO_2_ and regional changes in nitrogen deposition. Direct human management activities are simulated to have caused grasslands to switch from a sink to a source of greenhouse gas, because of increased livestock numbers and accelerated conversion of natural lands to pasture. However, climate change drivers contributed a net carbon sink in soil organic matter, mainly from the increased productivity of grasslands due to increased CO_2_ and nitrogen deposition. The net radiative forcing of all grasslands is currently close to neutral, but has been increasing since the 1960s. Here, we show that the net global climate warming caused by managed grassland cancels the net climate cooling from carbon sinks in sparsely grazed and natural grasslands. In the face of future climate change and increased demand for livestock products, these findings highlight the need to use sustainable management to preserve and enhance soil carbon storage in grasslands and to reduce greenhouse gas emissions from managed grasslands.

## Abstract (web summary)

Grasslands, and the livestock that live there, are dynamic sources and sinks of greenhouse gases, but what controls these fluxes remains poorly characterized. Here the authors show that on the global level, grasslands are climate neutral owing to the cancelling effects of managed vs. natural systems.

## Introduction

Grasslands are managed worldwide to support livestock production, with widely contrasting practices and intensity. Today, grass forage accounts for nearly half of the global intake of livestock^1^, although the proportion of grass-fed animals is decreasing as more use is made of crop-based animal feed^2^. Over the last century, grassland management has intensified across the world, with the number of domestic ruminants increasing dramatically—from 1.4 billion head to 3.4 billion^3^. This historical increase of domestic livestock was, however, preceded in the late nineteenth century by a massive extirpation of wild grazers that were hunted, killed by diseases, or confined by expanding agricultural lands^4^. The biomass of wild mammals today is an order of magnitude lower than that of livestock^5^. Changes in management intensity and the reduction of wild animal numbers alter the greenhouse gas (GHG) emissions and sinks of the world’s grasslands, and, together, determine the net radiative forcing (RF) of grasslands on climate change. The intensification of grassland management increases CH_4_ emissions from livestock and decreases those from wild grazers. As management becomes more intensive, N_2_O emissions, from the acceleration of nitrogen turnover and the fertilization of pasturelands, also increase. However, soil organic carbon stocks can increase or decrease alongside more intensive management, through complex interactions between grazing, soil carbon inputs and decomposition processes. Meta-analyses and literature reviews indicate that land-use^6^ and land-management changes^7–10^ have significant impacts on grassland soil carbon that differ between regions and climate zones^6,9^. Site-level data suggest that soil carbon stocks can increase, as long as grazing remains light to moderate^8,11–13^, but decrease under overgrazing, causing degradation^14,15^.

Assessing the historical GHG budgets of the different grassland biomes and their drivers is of key importance for understanding the trade-offs between grassland services on food security and climate mitigation and how current grassland management could be improved to meet low-warming climate targets. Global scale GHG emissions and sinks have been assessed for all the terrestrial ecosystems^16^, the agricultural sector^17^ and croplands^18^, but grasslands have been ignored, mainly because dealing with pasture management differences, and how they change with time, was deemed too complicated a problem. A few regional-scale estimates of the GHG budget of specific grassland types have been published^19–21^, but an assessment of the full GHG balance of the world’s grasslands, separating the direct human effect of intensified management and the indirect human effects of climate change, is still lacking.

**Fig. 1:**
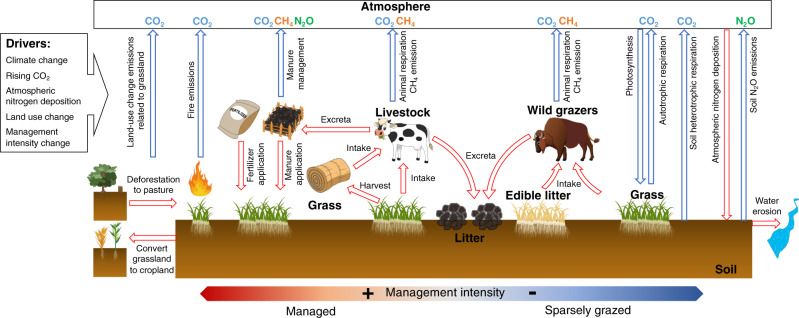
Illustration of the processes and the greenhouse gas fluxes that are accounted for in this study. The box with an arrow in the top left of the figure presents the drivers that used as input to the ORCHIDEE-GM v3.2. The processes and the greenhouse gas fluxes that are related to grassland are shown as red and blue arrows, respectively. The red-to-blue layout at the bottom of the figure indicates management intensity from high (red; intensively managed grassland) to low (blue; sparsely grazed and natural grassland).

Here, we address this research gap by quantifying the changes in carbon storage and GHG fluxes in natural and managed grasslands over the years 1750–2012. We consider the following processes: soil organic matter and plant productivity changes driven by historical shifts in livestock and wild grazers; fire and its interaction with grazing; soil carbon losses by water erosion; land-use change emissions related to grassland (pasture created by deforestation and the conversion of grassland to cropland); CH_4_ emissions from animals and excreta; N_2_O emissions from animal excreta, manure and mineral fertilizer applications; and atmospheric nitrogen deposition (Fig. 1). We use the spatially explicit, process-based ecosystem model ORCHIDEE-GM, which includes parameterizations of all these processes (Supplementary Methods 1). Although the model has previously been evaluated at regional and global scales^22,23^, here, we provide additional evaluation for the effects of overgrazing, leading to degradation and soil organic carbon loss, and the depth distribution of soil carbon incorporation. The model is run over the whole globe for the historical period 1860 to 2012 at a spatial resolution of 0.5 × 0.5°. It is forced by variable CO_2_, changing climate, nitrogen deposition, variable areas of pasture, and changes of animal density and per-capita grass intake^22^ (see Supplementary Table 1 for the simulation protocol and the time span of the input data). In the following, GHG fluxes are expressed in CO_2_ equivalent (CO_2_e) based on the global warming potentials of each gas on a 100-year time horizon^24^ to better combine the effects of emissions of different gases. The climate effect (i.e., RF) due to historical emissions and sinks of the three GHGs and to albedo changes related to grassland is calculated using a compact Earth system model OSCAR v3.1 (ref. ^25^) that accounts for the different lifetimes of the GHGs (see ‘Methods’).

## Results and discussion

### The GHG budget of grasslands

Estimates of the grassland fluxes of CO_2_, CH_4_ and N_2_O for the last five decades from ORCHIDEE-GM were evaluated against site observations, and regional GHG inventories (Supplementary Discussion 1 and Supplementary Table 2). We also compared our simulated GHG budgets with estimates from regional grassland models. The non-CO_2_ emissions estimated here were found to be consistent with all five inventories we compiled^1,26–29^. We compare simulated carbon budgets, that is, soil carbon changes, with published estimates at national/regional scale and with data from long-term ecological sites known as LTER (Supplementary Table 2). Observed soil carbon change and CO_2_ budgets are uncertain and spatially variable. The ORCHIDEE-GM model captures the sign of the carbon budget in 32 out of 42 studies (76%) and its results are within the 1-sigma uncertainty of 22 studies (52%). We found that the model results are within the 1-sigma uncertainty of literature estimates for a number of regions covering 42% of the global grassland area and 86% of the global mean grassland carbon budget during the period of 1980–2012, as simulated by the ORCHIDEE-GM. Model results are entirely outside of the 1-sigma uncertainty of literature estimates in regions covering only 0.3% of the global grassland area and 3% of the grassland carbon budget. This means our model tends to capture the grassland carbon budget in the important regions for a global assessment. The results from this comparison give us some confidence in using this model for long-term historical simulations. We also compared the vertical profile of the simulated grassland soil carbon change against those from a meta-analysis based on stable carbon isotope signature observations of soil profiles^30^ (see Supplementary Discussion 2). The results indicate that the modelled soil carbon age distribution and the recent soil carbon incorporation (since 1965) between the topsoil (0–30 cm) and subsoil (30–100 cm) are comparable with those from isotope observations of soil profiles^30^. This gives us some confidence in the values of residence time of soil organic carbon and carbon sequestration in the model. Further, a detailed analysis of how the model simulates regional losses of soil carbon and productivity in response to overgrazing was performed by comparing with the observed loss of productivity and soil carbon in site studies^11,31,32^ and by comparing the simulated overgrazing patterns with maps of grassland degradation derived from satellite data^33^ and regional surveys^34^ (see Supplementary Discussion 3). At the global scale, we compared, for information, the net carbon balance of grass-dominated grid cells in the ORCHIDEE-GM with the results of generic dynamic global vegetation models (TRENDY project) used in annual global carbon budget assessments. Note that the other global models do not incorporate detailed descriptions of changes in livestock, management practices and their feedbacks on ecosystem biogeochemistry (Fig. 1)^35^. We further compared the ORCHIDEE-GM net grassland–atmosphere CO_2_ flux against a top-down atmospheric inversion carbon sink^35^ after subtraction of forest^36^ and cropland CO_2_ budgets^18^. The results show that our estimate fell within the range of this observation-based large-scale constraint. Finally, net primary productivity (NPP), the trend of NPP and whole ecosystem carbon turnover in the model were compared against satellite-based (GIMMS NDVI^33^) and observation-driven data sets respectively (MODIS NPP^37,38^, GIMMS NPP^39^ and three soil carbon stock data sets at 1 m depth^40^). The results show that it is unlikely that our model overestimates the carbon sink of grasslands, given that: (i) it does not systematically overestimate NPP (i.e., not too high a carbon input) and (ii) simulates faster whole ecosystem carbon turnover than observation-driven estimates (i.e., not too low a carbon output; Supplementary Discussion 1).

Regarding the model’s capability of simulating overgrazed grasslands, and the response of productivity and soil organic carbon to different grazing intensities (Supplementary Discussion 3), we found that ORCHIDEE-GM captured the decreasing leaf area index (LAI) trend due to overgrazing in the western United States, southern Brazil, Argentina and Australia^33^, but underestimated overgrazing effects in central Asia and Sub-Saharan Africa. The historical model simulation also captures the areas classified as overgrazed in surveys from the 1980s in the Global Assessment of Soil Degradation (GLASOD) database^34^. The historical simulation showed an increase in overgrazed areas since the 1860s with the largest expansion of overgrazed grassland occurring in Africa. The modelled responses of soil organic carbon to different grazing intensities are comparable with those from meta-analysis based on in situ observations^11,31,32^. The above analysis gives us some confidence in using this model to simulate the global responses of grassland soil carbon and productivity to management.

**Fig. 2:**
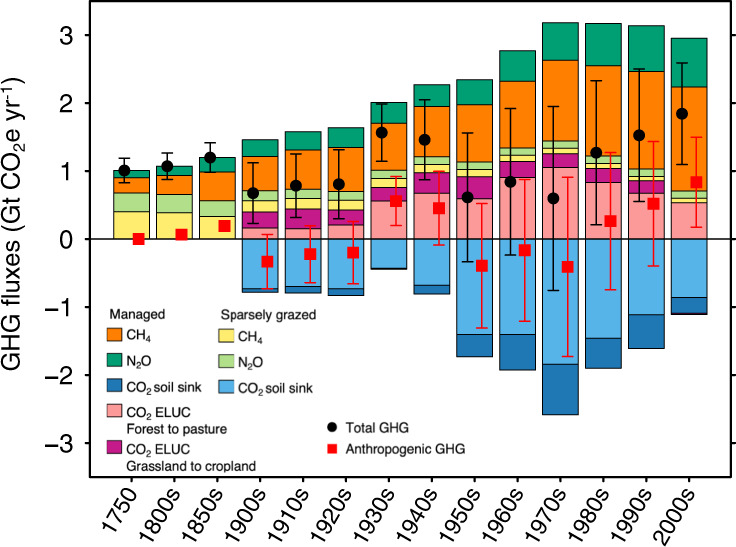
The decadal greenhouse gas (GHG) fluxes of global grassland during the period 1750–2009. Light and dark blue bars represent CO_2_ fluxes from managed and sparsely grazed grassland, respectively; orange and yellow represent CH_4_ fluxes from managed (domestic livestock) and sparsely grazed (wild grazers) grassland, respectively; light green and dark green represent N_2_O fluxes from managed and sparsely grazed grassland, respectively; and pink and purple represents land-use change emissions related to grassland from deforestation to pasture and from conversion of grassland to cropland, respectively. Black dots and their error bars indicate net total GHG balance and its 1-sigma uncertainty. Red squares and their error bars indicate the anthropogenic GHG balance after subtracting pre-industrial GHG fluxes. Negative values indicate GHG sinks and positive values indicate GHG sources.

The evolution of GHG fluxes during the last two and a half centuries (Fig. 2) shows that emissions of CH_4_ and N_2_O from grasslands have increased by a factor of 2.5 since 1750. This increase is mainly due to the intensification of management, with increased livestock numbers, greater turnover of manure and, in some regions, mineral nitrogen fertilizer addition. The largest historical emissions from deforestation to pasture occurred after the 1930s, following the large expansion of grazing land in Latin America^41^. Yet emissions from deforestation to pasture seem to show a decreasing trend after the 1970s in Latin America and East and Southeast Asia (Supplementary Fig. 1). In North America, Europe, Russia and South Asia, historical emissions from the conversion of grassland to cropland dominate land-use change emissions attributed to grassland. In contrast, grasslands globally have persistently absorbed CO_2_ from the atmosphere, resulting in an increase in soil carbon storage (Fig. 2).

Decadal fluctuations have occurred in the GHG balance of grasslands, with values ranging from a net GHG source of 0.6 ± 1.3 Gt CO_2_e yr^−1^ in the 1970s [all results are presented as mean ± standard deviation unless otherwise specified] to a source of 1.8 ± 0.7 Gt CO_2_e yr^−1^ during the 2000s (Fig. 2). Decadal variability of climate and of the area of deforestation are the two most important drivers that result in switching the sign of the decadal GHG balance of grasslands. In contrast, increasing atmospheric CO_2_ concentration, nitrogen deposition and management practices^22^ have slow trends that result in multi-decadal changes of GHG budgets of the same sign.

The increase of CH_4_ and N_2_O emissions from grasslands, shown in Fig. 2, is the sum of increased emissions from livestock^26^ (Supplementary Fig. 2) and decreased emissions from the reduced numbers of wild grazers (Supplementary Methods 2 and Supplementary Fig. 3). In our simulation we found that hunting, disease and reduced habitats due to expanding agriculture caused a decrease in CH_4_ and N_2_O emissions from wild grazers, from 0.7 ± 0.1 Gt CO_2_e yr^−1^ in 1750 to 0.3 ± 0.07 Gt CO_2_e yr^−1^ in 1900. Today, CH_4_ and N_2_O emissions are largely dominated by grass-fed livestock, and they amount to 1.6 ± 0.3 and 0.8 ± 0.3 Gt CO_2_e yr^−1^, respectively in 2005. These fluxes comprise 53 ± 9% and 42 ± 14%, respectively, of the emissions from the full livestock sector, that is, when including both grass-fed animals and those fed on crop-products^42^.

The net carbon sink in grasslands worldwide intensified over the last century (Fig. 2), mainly driven by North America, Europe and Russia (Supplementary Figs. 1 and 4). These increasing soil carbon sinks were due to the interaction between indirect human activities, like rising CO_2_ concentration, climate change (e.g., warming at high latitudes leading to higher LAI^43^ and grassland productivity^44^), atmospheric nitrogen deposition, and direct human activities like recent decreases of livestock numbers and pasture abandonment in Europe and Russia. Sparsely grazed and natural grasslands account for 80% of the total cumulative carbon sink of the world’s grasslands, and explain most of the current global sink (Fig. 2). Carbon emissions from deforestation to pasture are mostly contributed by South America, and East and Southeast Asia (70% and 21%, respectively), while carbon emissions from conversion of grassland to cropland are dominated by North America (39%), Europe (36%) and South Asia (21%). Over the last decade, managed grasslands are found to be a net GHG source of 2.0 ± 0.4 Gt CO_2_e yr^−^^1^ (land-use change emissions were excluded here to enable comparisons with the cropland estimates in ref. ^18^), that is, in mean value, similar to croplands which are a large GHG source of 2.0 ± 2.2 Gt CO_2_e yr^−1^ (ref. ^18^).

To disentangle the effects of management and climate change drivers on the carbon balance of grasslands, we conducted a series of simulations for the period 1860–2012 with one factor fixed at a time to enable us to attribute the contribution of different drivers (Supplementary Discussion 4). The increase of CO_2_ by 105 ppm since 1860 caused a net sink of CO_2_ (accounting for 85% of the net sink from all drivers including land-use change averaged for the period 1860–2012; Supplementary Fig. 5). The contribution of climate change is a carbon sink in some regions, but a carbon source in others, although, globally, it is a net sink (22%). The contribution of N deposition is comparable, being a global net sink (24% of the total net sink from all drivers). Human-caused increases of livestock densities, with larger sized animals over time, and extirpation of wild grazers, account for 39% of the global net sink including all drivers. In contrast, land-use change-related direct human activities associated with the conversion of forest to pasture and of grassland to cropland is responsible for a large net loss of carbon to the atmosphere, which offsets 141% of the net sink. A residual sink of 69% is due to nonlinear interactions of all these drivers. Net soil carbon removals induced by water erosion over grasslands resulted in a small source of 0.03 [0.03–0.06] Gt CO_2_e yr^−1^ in the 1860s, growing to a significant source of 0.23 [0.22–0.28] Gt CO_2_e yr^−^^1^ in the 1990s (Supplementary Discussion 5). These net removals comprise water erosion impacts on carbon removal and soil compensatory sinks from continuing litter input concurrent with less soil respiration due to topsoil carbon removal. Regarding fire regime changes resulting from intensified grassland management, the results of our simulation show that, altogether, management effects contributed 35% and 60% of the overall simulated downward trend of burned area during the period 2000–2012 in northern Africa and South America, respectively, through decreased fuel load as animal density increases (Supplementary Discussion 6). In northern Africa, this effect is larger than the contribution from expanding cropland from which fire is assumed to be excluded^45^.

Though various direct human activities and indirect effects are accounted for in our estimates, some other processes that are not considered may cause further uncertainties: (1) we account for fluxes from mineral soils only: carbon losses from peatland drainage for pasture are not considered. Peatlands drained for agriculture and forestry are substantial GHG sources, especially in the past few decades^46^. However, to our knowledge, historical emissions from pasture expansion on organic soils are not available; (2) though fire processes are simulated specifically, the formation of pyrogenic carbon is not considered in the model. Pyrogenic carbon, as the recalcitrant by-products of fire, can be stored in terrestrial ecosystem for centuries to millennia. However, it should be noted that the amount of pyrogenic carbon created from non-woody biomass burning (e.g., grasses burning) is small^47^ due to the relatively complete burning of non-woody components; (3) ozone’s impacts on the grassland carbon cycle are not considered. Elevated ozone (O_3_) concentrations may cause leaf damage, reduce biomass growth, and could contribute to changes in species composition^48^. However, a global data set and functions that quantify its effect on grassland productivity and soil carbon dynamics is not available.

**Fig. 3:**
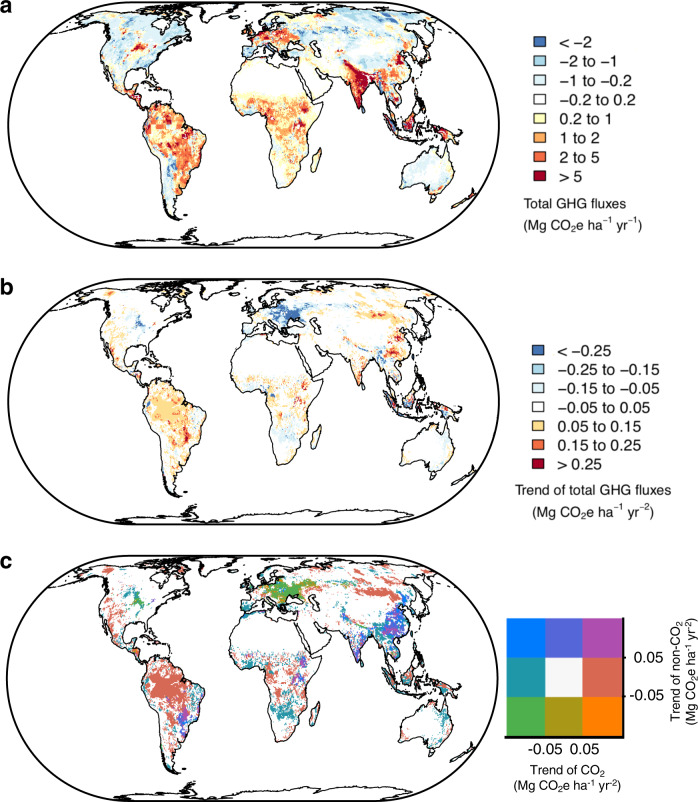
Global distribution of grassland greenhouse gas (GHG) balance during the period 1981–2010. **a** Mean value, **b** its trend, and **c** the trends of CO_2_ fluxes versus non-CO_2_ fluxes. Negative values in **a**, the total GHG fluxes, indicate GHG sinks and positive values indicate GHG sources. Negative values in **b**, the trend of total GHG fluxes, indicate enhanced GHG sinks or decreased GHG emissions, while positive values indicate increased GHG emissions or attenuated GHG sinks.

Figure 3 shows the spatial distribution of the GHG balance of grasslands and its trend over the past three decades. Net GHG sinks are located in temperate North America and Eurasian grasslands, especially in the tundra and dry grassland regions that have sparse livestock populations, and small CH_4_ and N_2_O emissions but significant carbon uptake (Supplementary Fig. 6). Conversely, net GHG sources are found in regions of intensive management with high livestock densities. Regional trends also have contrasts. On the one hand, grasslands in the central United States, eastern Europe and Russia show a decreasing trend in GHG emissions (Fig. 3b) coincident with decreasing livestock populations (Supplementary Fig. 2). In these regions, decreased livestock numbers have resulted in smaller CH_4_ and N_2_O emissions and enhanced soil carbon storage (Fig. 3c). On the other hand, China, Mongolia, eastern Africa and southern Brazil show positive trends of GHG emissions due to their rapidly increasing ruminant populations and, in the case of Brazil, the expansion of pasture over carbon-rich ecosystems (Fig. 3c).

### The net climate effect of grasslands

**Fig. 4:**
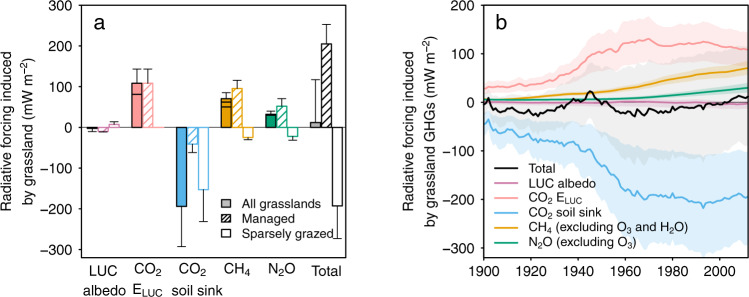
Radiative forcing induced by direct and indirect human activities on grasslands worldwide a in the year 2012 and b for the period 1900–2012. The ‘Total’ column/line is the total contribution of grassland to the global radiative forcing (RF), accounting for the land-use change induced albedo change related to grassland, land-use change emission related to grassland (CO_2_ *E*_LUC_) and the three grassland greenhouse gas (GHG) fluxes (CO_2_ soil sink, CH_4_ and N_2_O). In **a**, the RF of each component is further divided for managed and sparsely grazed grasslands, and is shown as an empty bar outlined in the same colour with (managed grasslands) and without (sparsely grazed grasslands) hatching, respectively. The stacked bar for CO_2_ *E*_LUC_ includes, from bottom to top, the effect from deforestation to pasture and conversion of grassland to cropland. The stacked bar for CH_4_ includes, from bottom to top, the direct effect from CH_4_, and its associated effects through tropospheric O_3_ and lifetime change induced by ozone precursors, and stratospheric H_2_O. The stacked bar for N_2_O includes, from bottom to top, the direct effect from N_2_O, and its associated effects through stratospheric O_3_. The uncertainties shown in **a** are 1-sigma standard deviation. The coloured shaded areas in **b** are the 1-sigma confidence interval derived from our uncertainty assessment (see ‘Methods').

The GHG balance of grasslands presented in the previous section contains pre-industrial fluxes and perturbations by human activities including both direct effects from land use and management changes and indirect effects from climate change, increased CO_2_ and anthropogenic atmospheric nitrogen deposition. After subtracting pre-industrial emissions (see ‘Methods’), the anthropogenic climate change effect of the grassland biome is found to be neutral (Fig. 2), that is, an RF of 12 ± 105 mW m^−2^ as calculated by the OSCAR Earth system model (Fig. 4a) (Supplementary Methods 2). In spite of this, the historical increase in livestock alone caused a substantial warming (147 ± 27 mW m^−2^ by CH_4_ and N_2_O emissions) partly balanced by a cooling from the reduced number of wild grazers −47 ± 11 mW m^−2^ (CH_4_ and N_2_O; Supplementary Table 3). The importance of the negative RF component from lost wild grazers has been overlooked in previous studies. Deforestation to pasture and the conversion of grassland to cropland caused net warming of 81 ± 26 and 27 ± 9 mW m^−2^, respectively. But the most intriguing result is that the cooling effect of carbon sinks (−194 ± 99 mW m^−2^) nearly offsets the warming effect of land-use change emissions and CH_4_ and N_2_O sources (209 ± 39 mW m^−^^2^), when their RF is compared (Supplementary Table 3). We show below that anthropogenic carbon sinks are mainly located in sparsely grazed and natural grasslands, whereas CO_2_ and non-CO_2_ sources prevail in managed grasslands.

**Fig. 5:**
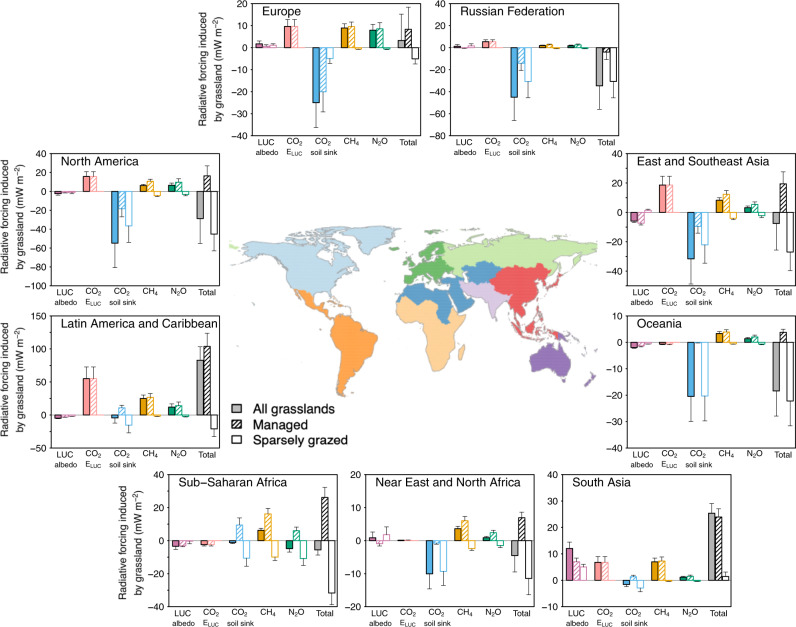
The contribution of different regions to the global radiative forcing of grasslands. The ‘Total’ column is the total contributions of grassland to the global radiative forcing (RF), accounting for the land-use change induced albedo change related to grassland, land-use change emission (CO_2_ *E*_LUC_) from deforestation to pasture and conversion of grassland to cropland, and the three grassland GHG fluxes (CO_2_ soil sink, CH_4_ and N_2_O). The RF of each component is further divided for managed and sparsely grazed grasslands, and is shown as an empty bar outlined in the same colour with (managed grasslands) and without (sparsely grazed grasslands) hatching, respectively. The uncertainties are 1-sigma standard deviation. The vertical-scale of the regional contribution has been adjusted so that the effects can be easily seen.

Regionally, grasslands in North America, Russia and Oceania have a small negative RF (Fig. 5) owing to increased carbon storage in soils (Supplementary Fig. 1). The grasslands of Sub-Saharan Africa also have a small net negative RF from the reduced number of wild grazers (reduced CH_4_ and N_2_O emissions in sparsely grazed and natural grasslands). Grasslands in South Asia have a positive RF due to a decreased albedo from pasture reduction, grassland conversion to cropland and livestock increase. The largest positive RF was ascribed to grasslands in Latin America, due to increases in livestock CH_4_ emissions, N_2_O from soils and deforestation-induced CO_2_ losses (Supplementary Fig. 1). Grassland in other regions has small positive or negative RF resulting from carbon sinks nearly compensating non-CO_2_ emissions.

### The cancelling climate effects of managed and sparsely grazed grasslands

Globally, the positive RF from managed grasslands, including emissions from land-use change related to grasslands, equals 205 ± 48 mW m^−2^ (Fig. 4a). This management-induced RF counter-balances the negative RF from sparsely grazed grasslands (–193 ± 80 mW m^−2^) (see ‘Methods'). This result illustrates the important climate cooling service provided by sparsely grazed areas, and the key role played by quickly increasing livestock numbers, associated with more CH_4_ and N_2_O emissions, in determining the contemporary net RF of the grassland biome.

Managed grasslands in all regions, except Russia, have a net warming effect on climate (Fig. 5). The direct human effect of grassland management on climate was analysed by comparison with a baseline simulation in which all grasslands would remain natural without livestock changes or fertilization (see ‘Methods' and Supplementary Table 1). The results indicate that management intensification caused 9% less soil carbon storage since the pre-industrial period (set to before 1750), because grazing and mowing reduced the carbon input to soils (i.e., overall carbon outputs from grassland ecosystem increased) more than they increased input via the stimulation of plant productivity through the generation of new leaves (Supplementary Figs. 7 and 8). In particular, regions with overgrazing have a decreased productivity and either smaller soil carbon gains or carbon losses (see Supplementary Discussion 3).

In contrast, regions with sparsely grazed grasslands provided an important climate cooling service, as in North America, Russia and Oceania (Fig. 5), through indirect climate change effects that enhanced carbon sinks. This result makes it clear that countries should assess not only the GHG budgets of their managed pastures (such as in the current national GHG reporting rules to the UNFCCC) but also the sinks/sources of sparsely grazed rangelands, steppes, tundra and natural grassland systems. Full GHG reporting for the entire territory of each country could facilitate the assessment of progress towards the goals of the Paris Agreement^49^ and better link national GHG budgets to the observed growth rates of GHGs in the atmosphere. Moving to a complete estimate of all GHG fluxes from grasslands calls for global models to incorporate a realistic representation of management practices, including livestock grazing, degradation, erosion and fires.

Today, on a global basis, managed grasslands provide half of the feed needed for livestock, with the other half coming from crop-based feed and residues. To fully assess the future climate impact of the livestock sector, or more generally, of the entire agricultural sector, the crop–feed-related GHG balance or emissions since 1750 would need to be included: a requirement that could be built upon data already compiled for the last decade^18^.

Despite increased livestock and management intensification, intriguingly, grasslands worldwide are found to exert no warming effect on climate. This conclusion is thanks to the presence of intensified carbon sinks, especially over sparsely grazed grasslands, which mainly result from the increased productivity of grasslands exposed to increased CO_2_ and nitrogen deposition. Maintaining this cooling service will be important for meeting the Paris Agreement targets. However, the recent trend (Fig. 4b) reveals that global grasslands are transitioning from a net cooling towards a net warming effect on climate. This trend can be attributed to the recent grassland management intensification for livestock production and the conversion of tropical forest to pasture. In the context of low-warming climate targets, the mitigating or amplifying role of grasslands will depend on: (1) future changes in grass-fed livestock numbers; (2) the stability of accumulated soil carbon in grasslands; and (3) whether carbon storage can be further increased over time, or if it will saturate, as observed in long-term experiments^50,51^. For example, a projected continual growth in bovine and ovine meat production and consumption in the near future, especially an accelerated growth of milk demand and production^52^, would result in a continual increase in grass biomass demand and thus increases in CH_4_ and N_2_O emissions. The future grassland net CO_2_ flux will depend on changes in environmental drivers such as warming, increased CO_2_ concentration, management intensity^53^, nitrogen addition, including fertilizer and atmospheric nutrient deposition, future land-use^6,54^, and fire regimes. To prevent further warming contributions from managed grasslands, sustainable management through optimized grazing, pasture improvement and restoration of degraded pasture^7,55,56^ are critical to enhance grassland soil carbon sequestration. Halting deforestation to pasture is also a priority in countries like Brazil where moderate intensification could avoid extensive cattle production and clearing of forests. Technologies reducing livestock CH_4_ emissions, such as improving feeding practices, specific agents and dietary additives, and longer-term management change and animal breeding^57,58^, and practices increasing nitrogen use efficiency and thus reducing N_2_O emissions from fertilization and manure (e.g., as collected in ref. ^59^), are also key for climate mitigation and should be explored in integrated assessments.

## Methods

### Methods overview

This study assesses CO_2_, CH_4_ and N_2_O fluxes in grassland ecosystems (including ecosystem-level fluxes from grasslands, and farm-level CH_4_ and N_2_O fluxes from management activities, i.e., grass forage consumption and manure management). The process-based land surface model ORCHIDEE-GM v3.2 (refs. ^22,60^) was used to simulate all carbon cycling flux and carbon pool components within ecosystems, between farm and ecosystem (i.e., grass forage from mown grasslands and managed manure applied to pasture), and CH_4_ and N_2_O emissions produced at the ecosystem and farm level. ORCHIDEE-GM v3.2 simulated the soil carbon changes of 11 soil layers. Litter input (structural and metabolic, above and below ground, is discretized in each model layer and decomposition is simulated from the fraction of three pools with different turnovers in each layer, modified by the temperature and soil moisture content of each layer which are simulated every 30 min by the land surface model. The model distinguishes explicitly between sparsely grazed and managed grassland, and calculates emissions from domestic livestock^22^ and wild grazers^4^. Populations of wild grazers since 1750 were reconstructed on a 0.5° global grid (Supplementary Methods 1). Pre-industrial CH_4_ and N_2_O fluxes are subtracted from historical values to quantify post-industrial anthropogenic fluxes. To calculate the RF induced by each separate anthropogenic flux (nine different regions, three gases with a separation between land-use change emissions and soil carbon storage for CO_2_, managed pastures and sparsely grazed grasslands), the compact Earth system model OSCAR v3.1 (refs. ^25,61,62^) was used. This model is specifically designed to attribute regional and global climate change to multiple forcings and feedbacks^63–65^. Here, OSCAR is driven by GHG flux distributions from ORCHIDEE-GM. These drivers are integrated to disentangle the effect of each climate forcer for managed grasslands (mown, grazed by livestock, fertilized) versus sparsely grazed and natural grasslands for nine regions of the globe. To calculate the contribution of grassland to the land-use change induced albedo change and associated RF, we used a diagnostic method that combines present-day satellite data of surface albedo with historical land–cover maps^66^ (see also ref. ^61^).

### The estimation of GHG fluxes and their uncertainty

ORCHIDEE-GM v3.2 (refs. ^22,60^), which includes specific parameterizations of grassland management^67^, was applied over the entire globe, at a spatial resolution of 0.5° × 0.5°, with variable CO_2_, climate, atmospheric nitrogen deposition, land-use change and management intensity (including manure and mineral fertilizer application, grazing density and managed area)^22^. Historical changes of grass biomass consumption (biomass intake during grazing and harvested grass forage for confined livestock, including landless production systems^1^) were reconstructed from historical data^1,22^, and prescribed as input data to the model^22^. A detailed description of the model, inputs and simulation setups can be found in Supplementary Methods 1. We defined grassland areas as a dynamic variable including all lands that have been grasslands (natural or human managed) since 1750. For the period of 1860–2012, grassland areas were defined by prescribed historical land-cover maps^68^ (Supplementary Table 4), and managed (i.e., mown, grazed and fertilized) and sparsely grazed grassland areas were reconstructed using the method detailed in ref. ^22^ (Supplementary Fig. 2 and Supplementary Methods 1). We assumed no changes in grassland areas between 1750 and 1860 due to lack of data. Grassland area changed annually across history in the historical land-cover maps^68^ from 51.7 million km^2^ in 1860 to 49.7 million km^2^ in 2012. Managed grasslands are defined as all grasslands used to feed livestock (i.e. grazed and/or mown for grass forage, fertilized in some regions), and sparsely grazed grasslands are natural grasslands not affected by livestock but may be sparsely grazed by wild grazers. A detailed discussion on the grassland and managed grassland areas, and the distributions used here, can be found in Supplementary Discussion 7. Fire^69,70^ and interactions between grazing and fire are explicitly represented in ORCHIDEE-GM (Supplementary Discussion 6). Though the model does not simulate erosion explicitly, we account for grassland carbon fluxes due to water erosion by using estimates from a model framework combining the carbon cycle of ORCHIDEE-MICT^71^ (a similar version that ORCHIDEE-GM v3.2 is built on) and a modified version of the Revised Universal Soil Loss Equation (RUSLE) model that is applicable at a large spatial scale^72^ (Supplementary Discussion 5). It should be noted that only mineral soils were considered in this study, since the model does not simulate peatlands (organic soils). Grassland GHG fluxes due to land-use change from sparsely grazed grasslands to managed grasslands are explicitly simulated by the model, given reconstructed historical management intensity changes. For each year, we estimated the grassland GHG balance, including new grasslands converted from other land-cover types (like forests and croplands), and land-use change emissions (CO_2_ *E*_LUC_) related to grassland. CH_4_ and N_2_O emissions following the conversion of natural ecosystems to pasture and the increased livestock density, caused by livestock digestion and excreta deposition, were accounted for through the changes in grass biomass consumption.

In ORCHIDEE-GM, carbon stocks and associated carbon fluxes for land-cover transitions involving grasslands are attributed to the original source land cover type if it is a grassland type. For instance, the carbon balance of a cropland created from a grassland is reported under grassland. The carbon balance of a forest cleared to pasture is also reported under grassland. However, in the latter case, legacy emissions from harvested wood products during deforestation to pasture were not included in the land use-induced component of the grassland GHG balance.

In our estimates of the net grassland GHG balance induced by land use change, with the above definitions, carbon losses due to deforestation to pasture, which is dominated by biomass loss^73^ rather than changes in soil organic carbon^6^, and due to conversion of grassland to cropland were separated with factorial simulations (CO_2_ *E*_LUC_) as in the DGVMs that were run for the global annual carbon budget assessments from the Global Carbon Project^35^. Specifically, three sets of simulations were performed with ORCHIDEE-GM. The first set was forced with a time-invariant pre-industrial land-cover distribution. The second simulation was forced with historical land-use transitions from forest to grassland (with invariant cropland areas as in 1860, in order not to include cropland land-use change GHG emissions), climate, atmospheric CO_2_ concentration, and nitrogen deposition. The third simulation was forced with historical land-use transitions from grassland to cropland (with invariant forest areas as in 1860), climate, atmospheric CO_2_ concentration, and nitrogen deposition. Land-use change emissions from deforestation to pasture were diagnosed by the difference between the first and the second simulations. Land-use change emissions from conversion of grassland to cropland were diagnosed by the difference between the first and the third simulations. We note that this approach to quantify land cover change emissions involving grasslands includes a foregone sink named the lost atmospheric sink capacity^74,75^ and thus may give higher values in the recent period than emissions calculated by land use bookkeeping models (e.g. ref. ^76^).

The grassland GHG balance at ecosystem and farm scale includes CO_2_, CH_4_ and N_2_O fluxes of grassland ecosystems and off-site CO_2_, CH_4_ and N_2_O emissions from the digestion of harvested forage by livestock and manure management. The CO_2_ and CH_4_ fluxes of the grassland ecosystem were simulated by ORCHIDEE-GM. Tier 1 or Tier 2 methods of the 2006 IPCC guidelines for national greenhouse gas inventories^77^ were used for calculating N_2_O emissions from managed soils, CH_4_ emissions from digestion of harvested forage, and N_2_O and CH_4_ emissions from manure management. The activity data required for estimating N_2_O and CH_4_ emissions through IPCC Tier 1 or Tier 2 methods were either simulated by ORCHIDEE-GM v3.2 (like grazed biomass, excreta nitrogen deposited as urine and dung by grazing animals on grassland, harvested grass forage, and excreta nitrogen from housed livestock consuming harvested grass forage), or model inputs (like manure and fertilizer application, and atmospheric nitrogen deposition). It should be noted that potential CH_4_ and N_2_O uptake by grassland soils is not taken into account here. The grassland GHG balance (unit: Gt CO_2e_ yr^−^^1^) was calculated as

1 $${\mathrm{{GHG}}} = F_{{\mathrm{{CO}}}_2 \mbox{-} {\mathrm{C}}} \times \frac{44}{12} + E_{{\mathrm{{LUC}}} \mbox{-} {\mathrm{{grass}}}} \times \frac{44}{12} + F_{{\mathrm{{CH}}}_4 \mbox{-} {\mathrm{C}}} \times \frac{16}{12} \times {\mathrm{{GWP}}}_{{\mathrm{{CH}}}_4} + F_{{\mathrm{{N}}}_2{\mathrm{{O}}} {\mbox {-}} {\mathrm{{N}}}} \times \frac{44}{28} \times {\mathrm{{GWP}}}_{{\mathrm{{N}}}_2{\mathrm{{O}}}},$$

where the molecular weight conversion factors 44/12, 16/12 and 44/28 are used to convert the mass of CO_2_-C, CH_4_-C and N_2_O-N into CO_2_, CH_4_ and N_2_O, respectively. ${\mathrm{GWP}}_{\rm{CH}_4}$ (Gt CO_2e_ per Gt CH_4_) and ${\rm{GWP}}_{\rm{N}_2\rm{O}}$ (Gt CO_2e_ per Gt N_2_O) are global warming potential constants used to give the integrated RF of CH_4_ and N_2_O in terms of their CO_2_ equivalent. Here, we adopt the GWP for a time horizon of 100 years, with inclusion of climate-carbon feedbacks (i.e., GWP100 AR5 + OSCAR +  IRF and REs updates in Table 2 of ref. ^24^) of 34 and 267 for CH_4_ and N_2_O, respectively. $F_{\rm{CO}_2{\mbox{-}}\rm{C}}, F_{\rm{CH}_4\mbox{-}\rm{C}}$ and $F_{\rm{N}_2\rm{O}\mbox{-}\rm{N}}$ are annual fluxes of CO_2_, CH_4_ and N_2_O, respectively (unit: Pg C yr^−1^ or Pg N yr^−1^), between the grassland ecosystem/farm system and the atmosphere. *E*_LUC-grass_ (unit: Pg C yr^−1^) is the land-use change emissions from deforestation to pasture. All the GHG flux calculations in this study were made on a grid-cell basis. A detailed description of the estimation of the GHG fluxes can be found in Supplementary Methods 1.

The uncertainty in the regional and global GHG fluxes and their components was assessed using a Monte Carlo approach (*n* = 10,000). Given the scarcity of evaluation data for changes in grassland soil carbon, we estimated the uncertainty of the grassland carbon budget by comparing the simulated carbon balance with a compilation of observations at site and regional level obtained from an extensive literature survey (Supplementary Discussion 1). The relative uncertainty was estimated as the relative standard error of the mean distance between the simulated carbon budgets and the observations collated from the literature, with model output sampled across the grid-points or regions with available data. In total, 22 observation-based studies (Supplementary Table 2) are used for estimating the relative uncertainty, while regional estimates from ecosystem models, or estimates giving only a range of observations (i.e., without mean values), are excluded. We derived a relative uncertainty of 46%. It is noteworthy that the relative uncertainty derived from model structural uncertainty from 16 TRENDYv6 models (42%; Supplementary Table 5) is as large as that estimated by model-data comparison. Here, the relative uncertainty for land-use change emissions related to grassland (31%) is derived directly from the estimates of the TRENDYv6 models^35^ (standard deviation divided by multi-model ensemble mean; Supplementary Table 5). For each element of the Monte Carlo approach, we randomly selected regional/global CO_2_ fluxes (e.g., grassland CO_2_ fluxes and land-use change emissions) from their distributions. For CH_4_ and N_2_O, we assessed regional and global CH_4_ and N_2_O emission uncertainties due to emission-related factors (Supplementary Methods 1) through a Monte Carlo approach (*n* =  10,000). For each element of the Monte Carlo approach, we randomly selected emission-related factors from their distributions. Following a suggestion by ref. ^77^ (Vol 4, Chapter 3), a normal distribution was assumed for factors with symmetric uncertainty, and a lognormal distribution was assumed for factors with asymmetric ranges of uncertainty.

### The estimation of anthropogenic GHG fluxes

Following the IPCC^78^, we defined the year 2012 as the present day and the year 1750 to be the end of pre-industrial times. Given the existence of domestic livestock and wild grazers in the pre-industrial era, CH_4_ and N_2_O emissions from both domestic livestock and wild grazers before 1750 do not contribute to the global RF. They were estimated in this study to emit 13.8 ± 2.7 Tg C yr^−1^ of CH_4_ and 0.9 ± 0.3 Tg N yr^−1^ of N_2_O, for a total of 1.0 ± 0.2 Gt CO_2_e yr^−1^ (converted using GWP100 with climate-carbon feedback^24^), while global grasslands were assumed to be CO_2_ neutral during pre-industrial times. Therefore, for the purpose of attributing the contribution of grassland’s GHG fluxes to RF, we defined the human-induced (anthropogenic) GHG fluxes of grassland by subtracting pre-industrial CH_4_ and N_2_O emissions from the historical/contemporary GHG fluxes. The net CO_2_ fluxes from sparsely grazed grasslands were also considered to be human-induced, because they are driven by indirect human drivers.

The anthropogenic GHG fluxes from grassland, for the period 1750–2012, were used by OSCAR v3.1 to calculate the grassland contribution to RF. The total (i.e. anthropogenic + pre-industrial) GHG fluxes for the period 1860–2010 were either directly simulated by ORCHIDEE-GM, or estimated through IPCC Tier 1 or Tier 2 methods using active data simulated by ORCHIDEE-GM or as model inputs. They were extrapolated over the period 1750–1859 following a set of assumptions. First, grassland is assumed to be CO_2_ neutral with net CO_2_ fluxes of 0 Gt CO_2_ yr^−1^. Second, CH_4_ and N_2_O emissions from managed grasslands (i.e., mainly from domestic ruminants, and to a lesser extent N_2_O emissions from atmospheric nitrogen deposition) were varied following the human population variation in each region, with the emissions of 1860 used as the reference value. Third, CH_4_ and N_2_O emissions from sparsely grazed grasslands (i.e., mainly from wild grazers, and to a small extent from N_2_O emissions from atmospheric nitrogen deposition) were varied, following the reconstructed regional wild grazer population, over the period 1800–1859 (see Supplementary Methods 1 for details), but kept constant, at the 1800 value, over the period 1750–1799. The human population density since 1750 was retrieved from the History Database of the Global Environment (HYDE) compiled by the Netherlands Environmental Assessment Agency^41^. The HYDE gridded data are available for the beginning of each decade from 1750 until 1860. Annual data were linearly interpolated within each decade.

### Attribution of RF induced by anthropogenic GHG fluxes from grasslands

In all the simulations by OSCAR v3.1 (ref. ^25^), climate change was forced and based on observations. The model was fed by reconstructions of past climate change following HadCRUT4 (ref. ^79^ and updates in 2018) for global temperature, HadISST1 (ref. ^80^ and updates in 2018) for sea surface temperature, and CRU^81^ for regional land temperature and precipitation. The oldest 30-year data period was assumed to correspond to the pre-industrial climate. The anthropogenic GHG fluxes from grassland since 1750 were used as input for OSCAR v3.1. The uncertainty in those simulations was assessed via a Monte Carlo approach (*n* = 10,000) to the OSCAR simulations, in which biophysical parameters of the model were randomly drawn (see ref. ^61^) and associated to every single set of anthropogenic grassland GHG fluxes of the first Monte Carlo ensemble. Because OSCAR naturally produces a range of uncertainty larger than we considered to be reasonable for the three GHGs simulated in this study, we weighted our Monte Carlo ensemble using existing emission inventory data sets as constraints (see Supplementary Methods 2 for details).

The attribution is made to nine regions and eight sectors. Sectors are defined as emissions of CO_2_, CH_4_ and N_2_O from managed versus sparsely grazed grasslands, and of CO_2_ from land-use change emissions of deforestation to pasture and of conversion of grassland to cropland. The attribution protocol requires that, in addition to a control experiment, 73 simulations are made, one for each combination of region and sector, plus one for the rest (i.e. all the remaining emitting sectors unrelated to grasslands). In each of these simulations, the same emissions as in the control experiment are prescribed, except that a small fraction of 1% of the region’s sectoral emissions are removed. The difference between the control simulation and each of those factorial simulations, once normalized by the sum of the 73 differences, provides the contribution of each region and sector. This approach to attribution is called the normalized marginal method, and it is described further by ref. ^65^ and references therein.

The RF induced by albedo changes in grassland-related land cover (${\mathrm{RF}}_{{\mathrm{grass}}}^{{\mathrm{LCC}}}$) was assessed using the same method as used by OSCAR v3.1 (ref. ^25^) derived from ref. ^66^. The ${\mathrm{RF}}_{{\mathrm{grass}}}^{{\mathrm{LCC}}}$ was calculated, using a time step of one year, following annual changes of grassland area across history in the historical land-cover maps^68^. Here, grassland-related land-cover change was defined as both grasslands converted from (grassland increase) and to (grassland decrease) other land-cover groups (i.e., other biomes). In this case, any RF between two biomes should be equally attributed to the biome with decreased area and the biome with increased area. To ensure that the sum of biome-specific RF (i.e., $\mathop {\sum }\nolimits_b {\mathrm{RF}}_b^{{\mathrm{LCC}}}$) was equal to the RF from albedo change induced by all land-cover changes, and therefore to avoid double counting, we divided the final ${\mathrm{RF}}_{{\mathrm{grass}}}^{{\mathrm{LCC}}}$ by 2, as in Supplementary Methods 2, Equation (45). The uncertainty of ${\mathrm{RF}}_{{\mathrm{grass}}}^{{\mathrm{LCC}}}$ was assessed as the standard deviation of the 18 estimates of ${\mathrm{RF}}_{{\mathrm{grass}}}^{{\mathrm{LCC}}}$ derived from the combinations of different data sets: three land-cover climatologies, three radiation flux climatologies, and two albedo climatologies (see Supplementary Methods 2 for detail). The total current global RF induced by land-use change induced albedo estimated in this study (–0.09 ± 0.02 W m^−2^; Supplementary Fig. 9) is within the uncertainty range of the IPCC estimate of –0.15 ± 0.10 W m^−2^ (ref. ^78^). Values of ${\mathrm{RF}}_{{\mathrm{grass}}}^{{\mathrm{LCC}}}$ for managed and sparsely grazed grasslands (see above for the definitions) were also separated, and the same was done for the anthropogenic GHG fluxes and their RF values.

## Supplementary information

Supplementary Information

## Data Availability

The data that support the findings of this study are available at the public Data Repository of the International Institute of Applied Systems Analysis (IIASA DARE; https://dare.iiasa.ac.at/110; DOI: 10.22022/esm/11-2020.110).

## Code availability

The source code for ORCHIDEE-GM v3.2 is available at 10.14768/20190319001.1. The source code of OSCAR is available at https://github.com/tgasser/OSCAR (10.5281/zenodo.4282113). The code used to generate all the results of this study and the specific scripts used to execute the attribution with OSCAR are available from the corresponding author upon request.
