# Supplementary material for: Climate warming from managed grasslands cancels the cooling effect of carbon sinks in sparsely grazed and natural grasslands
Source: Nat Commun. 2021 Jan 5;12:118. doi: 10.1038/s41467-020-20406-7 (PMC7785734; doi:10.1038/s41467-020-20406-7)
Supplement: Supplementary file 1 — Supplementary Information [file 41467_2020_20406_MOESM1_ESM.pdf]

Supplementary Information for:

Climate warming from managed grasslands cancels the cooling effect of carbon sinks in sparsely grazed and natural grasslands

Jinfeng Chang[1,2,3]*, Philippe Ciais[1], Thomas Gasser[2], Pete Smith[4], Mario Herrero[5], Petr Havlik[2], Michael Obersteiner[2], Bertrand Guenet[1], Daniel S. Goll[1], Wei Li[1], Victoria Naipal[6], Shushi Peng[7], Chunjing Qiu[1], Hanqin Tian[8], Nicolas Viovy[1], Chao Yue[9], Dan Zhu[1]

[1] Laboratoire des Sciences du Climat et de l'Environnement, LSCE/IPSL, CEA-CNRS-UVSQ, Université Paris-Saclay, 91191 Gif-sur-Yvette, France

[2] International Institute for Applied Systems Analysis, A-2361 Laxenburg, Austria

[3] College of Environmental and Resource Sciences, Zhejiang University, Hangzhou, 310058, China

[4] Institute of Biological & Environmental Sciences, University of Aberdeen, 23 St Machar Drive, Aberdeen, AB24 3UU, UK

[5] Commonwealth Scientific and Industrial Research Organization, St Lucia, QLD 4067, Australia

[6] Ludwig-Maximilian University, Munich, Germany

[7] Sino-French Institute for Earth System Science, College of Urban and Environmental Sciences, Peking University, Beijing 100871, China

[8] International Center for Climate and Global Change Research and School of Forestry and Wildlife Sciences, Auburn University, Auburn, AL, USA

[9] State Key Laboratory of Soil Erosion and Dryland Farming on the Loess Plateau, Northwest A&F University, Yangling, Shaanxi 712100, P.R. China

*To whom correspondence should be addressed: changjf@zju.edu.cn

Table of content:

Supplementary Discussion 1. Comparison of the greenhouse gas fluxes from the literature and those simulated in this study
Supplementary Discussion 2. Vertical profiles of the simulated grassland soil carbon change
Supplementary Discussion 3. Model's responses to changes in grassland management
Supplementary Discussion 4. Attribution of the simulated grassland carbon balance to different drivers
Supplementary Discussion 5. Impact of erosion on the grassland carbon budget
Supplementary Discussion 6. Effect of grassland management on the simulated fuel loads, burned area and fire emissions
Supplementary Discussion 7. Grassland area, managed grassland area and management intensity
Supplementary Discussion 8. Effect of rising atmospheric CO_2 concentration on the simulated productivity
Supplementary Discussion 9. The climate effect of grassland greenhouse gas fluxes
Supplementary Methods 1. Model and methods used for grassland greenhouse gas assessment
Supplementary Methods 2. Model and methods used for radiative forcing attribution

Supplementary Discussion 1. Comparison of the greenhouse gas fluxes from the literature and those simulated in this study

Here, we validate our estimates of contemporary CO_2, CH_4 and N_2O fluxes against individual gas estimates derived from diverse methodologies (Supplementary Table 2; Supplementary Figures 10–14).

We estimate that the cooling effect of carbon sinks in grasslands worldwide has outweighed the emissions of deforestation for pasture, and has offset part of the CH_4 and N_2O emissions. Quantifying the net carbon sink of global grassland is a critical endeavour that requires careful validation. Unfortunately, as far as we know, no direct observations of the global grassland carbon budget are available. We therefore adopt the following three steps to validate the grassland carbon budget and its components indirectly at global scale and, for its components, directly at national/regional scale. From a global perspective, we first validate the simulated net land carbon budget, and that from all grass-dominated grid cells, against estimates from a set of dynamic global vegetation models (DGVMs; TRENDY project) used in annual global carbon budget assessment[1]. We then constrain the recent grassland carbon budget using indirect global land carbon budget estimates. In a second step, we validate the critical components affecting the budget, including net primary productivity (NPP), the trend of NPP, and whole ecosystem carbon turnover, against satellite-based and observation-driven data sets, respectively. Finally, we compare the simulated budgets with estimates from the literature derived using diverse methodologies at national/regional scale and from long-term grassland experimental sites.

Over the past five decades, the net global land carbon sink simulated by ORCHIDEE-GM v3.2 is well within the uncertainty range of the multi-model ensemble of TRENDY v6 models (Supplementary Figure 10a). When the carbon budgets over grass-dominated grid cells (with grassland covering over 60% of the land area in 2000, according to the land-cover change map used in this study) are extracted, the comparison again shows that our estimated sink is within the uncertainty range of the multi-model ensemble mean of TRENDY v6 models (Supplementary Figure 10b). Although no global estimate of the grassland carbon budget is available, we can constrain it with the available indirect estimates of the land carbon budget and those from non-grassland ecosystems. Global carbon budget assessment[1] estimated a terrestrial land sink of 2.6 ± 0.9 Gt C yr^{-1} for 1990 to 2007. Forest ecosystems sequestrated 2.4 $\pm$ 0.4 Gt C yr^{-1} during the same period[2], with the rest (0.2 ± 0.9 Gt C yr^{-1}) contributed by cropland and grassland. Carlson et al.[3] estimated a carbon loss from global cropland, due to

peatland draining, of about 0.15 ± 0.02 Gt C yr^{-1}. Thus, the grassland carbon budget can be indirectly constrained to be a sink of 0.35 ± 0.9 Gt C yr^{-1}, assuming no other net carbon loss or gain over cropland. Our estimate (0.37 ± 0.19 Gt C yr^{-1} for 1990 to 2007) fell within this constrained grassland budget range.

Ecosystem NPP is a major carbon input, and soil + vegetation carbon turnover is a major carbon output: both affect the net carbon budget of grasslands.

We compare modelled mean annual NPP against those from two satellite-based models (GIMMS NPP[4]; and MODIS NPP[5,6]; data from 2000–2011 were used) over grass-dominated grid cells (Supplementary Figure 11). Both positive and negative model-data differences are found. The results show that our model does not systematically overestimate the NPP of grass-dominated grid cells. In addition to mean annual NPP, the NPP trend also affects the grassland carbon budget and its trend[7,8]. Thus we validate the trend of annual NPP simulated by ORCHIDEE-GM against the trend of annual mean GIMMS NDVI[9] during the period of 1982–2010 over grass-dominated grid cells. Though NDVI, an index of greenness, does not represent the productivity directly, it is a useful proxy for trends in productivity[10], albeit with some uncertainty[11]. Spatially, our model is generally good at capturing the phase of the trends shown in the GIMSS NDVI data over grass-dominated grid cells (Supplementary Figure 12).

Soil carbon turnover time is defined as soil carbon stock divided by soil respiration, with the implicit assumption that the ecosystem is in a steady state. Several data sets of global soil carbon stocks exist. However, a global data-driven soil carbon turnover product with consistent respiration and carbon stock products is not available. To evaluate model performance on carbon turnover time, we adopt the "apparent whole ecosystem carbon turnover time" over grasslands as the ratio between soil + vegetation carbon stocks and net primary productivity (NPP) following ref [12]. We do not use the whole ecosystem carbon stocks including soil and vegetation carbon stock, given the fact that 1) there is no global product of grass biomass; and 2) biomass only comprises a small fraction of the ecosystem carbon stocks in grassland ecosystems. The apparent whole ecosystem carbon turnover time from model simulations can thus be evaluated against data-driven estimates using data-based soil carbon stocks and remote sensing NPP products. For this comparison, we use six sets of apparent whole ecosystem carbon turnover times, derived from the combination of two satellite-based models of NPP (MODIS NPP[5,6]; and GIMMS NPP[4]), and three soil carbon stock data sets at 1 metre depth (derived from ref[13]; one is from SoilGrids[14,15], and two are from the Harmonized World Soil Database

(HWSD[16]) using SOTWIS bulk densities and Saxton bulk densities[17], respectively). The comparison (Supplementary Figure 13) shows that our model tended to underestimate whole ecosystem carbon turnover time (i.e., we simulated a faster whole ecosystem carbon turnover) compared to the mean estimate of the six sets. Given that our model: i) does not systematically overestimate NPP (i.e., not too high a whole ecosystem carbon input), and ii) simulates faster whole ecosystem carbon turnover than observation-driven estimates (i.e., not too low a carbon output), it is not likely that our model simulates a large overestimate of the carbon sink of grasslands. However, it should be noted that the apparent whole ecosystem carbon turnover time adopted in this study tends to be smaller (i.e., faster turnover) than actual soil carbon turnover time, because the former overestimates the soil carbon input as not all NPP enters the soil. It is particularly the case for intensively managed grasslands, where a higher fraction of NPP is removed by grazing and mowing.

National and regional estimates of grassland carbon budgets from previous studies were collected to validate the budgets simulated in this study (Supplementary Table 2; Supplementary Figure 14). Various methods were used to estimate the grassland carbon budget: soil organic carbon (SOC) inventories (repeated soil samplings or regional comparisons), up-scaling fluxes from tower observations, atmospheric inversion models, remote sensing plus empirical models, process-based models calibrated for specific regions, and combinations of independent approaches. Spatially, most estimates relate to North America, Europe and China, with one estimate each for Brazil, New Zealand and the Russian Federation.

Over North America, our estimates are generally within the range of regional estimates from the different methods (SOCCR-2, adapted from ref[18]; and ref[19]), but higher than those from soil carbon inventories combining process-based models and the IPCC Tier 2 method [20]. For the United States, our simulated carbon sink is comparable with the estimates based on flux tower measurements[21] and regional process-based models in The Land Carbon Project[22-24].

Over Europe, our estimates are comparable with regional estimates based on flux tower measurements across Europe[25] and those from a combination of independent approaches[26,27]. However, the simulated carbon sink over grasslands in the United Kingdom is not consistent with estimates from soil carbon inventories, in which carbon loss[28] or insignificant soil carbon change[29,30] was found. Over the grasslands of Belgium, our model tends to simulate a larger carbon sink than estimates from soil carbon inventories. Although the results do not agree with each other – a carbon sink[31-33], a source [34,35] and a non-significant change[36] were all estimated.

For grasslands in the Netherlands, we have conservative carbon sink estimates compared to those from soil carbon inventories.

In Brazil, ref[37] estimated a strong carbon source over grasslands using land-use/management induced soil carbon loss from the literature and grassland areas. Our model simulates a carbon loss over Brazilian managed grassland soils (8 ± 4 Tg C yr^{-1}), a carbon sink for sparsely grazed grassland (-14 ± 6 Tg C yr^{-1}), and a large land-use change carbon source due to deforestation for pasture (91 ± 23 Tg C yr^{-1}). The total balance of 85 ± 28 Tg C yr^{-1} is consistent with the estimates from ref[37]. We estimate a neutral carbon balance of -3 ± 1 g C m^{-2} yr^{-1} over the grasslands of New Zealand for the past decades, which is within the range of measured values which range from a sink of 100 ± 37 gC m^{-2} yr^{-1} to a source of 121 ± 25 gC m^{-2} yr^{-1} (from different grassland forms[38]). For grasslands in Russia, we estimate a conservative carbon sink of 122 ± 56 Tg C yr^{-1} around 1990, compared to a stronger sink of 197 Tg C yr^{-1} reported by ref[39]. Our lower estimate may be due to the fact that we simulated a faster whole ecosystem carbon turnover than data-driven estimates derived from remote-sensing NPP and a soil carbon database (see above comparison on the whole ecosystem carbon turnover time).

Over the grasslands of China, our estimate is consistent with the national estimate compiled from a combination of independent approaches[40]. For temperate grassland in northern China, our estimates are within the uncertainty range of estimates from a soil carbon inventory[41], and are conservative compared to other estimates using different methods[42-45]. For grasslands in southern China, only two estimates of the carbon budget from regional process-based models are available. Our estimates are consistent with one estimate[46], but have a stronger sink than the other[47]. For grasslands in the Qinghai-Tibetan plateau, our estimates are within the uncertainty range of estimates from a soil carbon inventory[48], have a consistent sink with values from the other inventory[49], and are comparable with estimates from other process-based models[50-53].

Measured changes of soil organic carbon at three long-term grassland experimental sites were collected (one site in the United States[54]; two sites in the United Kingdom[55]). For comparability, we conducted site simulations at LTER sites with exactly the same model version and the same parameterizations as those in the global simulation: i) using CRU-NCEP climate forcings around the grid cell where the experimental sites are located (using a grid cell with the mean annual temperature and annual total precipitation closest to the values reported in the literature), and ii) applying the same land use change trajectory and grassland management practices as

indicated in the literature for each LTER site*. We found that ORCHIDEE-GM v3.2 reproduced a carbon loss at the Kellogg virgin LTER grassland site in the United States (68 ± 31 g C m^{-2} yr^{-1}) consistent with the observations (86.5 g C m^{-2} yr^{-1}). It should be noted that a relative uncertainty of 46%, estimated as the relative standard error of the mean distance between simulated carbon budgets and compiled observations from the literature (see Methods for detail), was applied for the modelled carbon balance at long-term grassland experimental sites. At Palace Leas and Park Grass in the United Kingdom, no significant SOC differences between the two inventories are found when accounting for all plots. Our model simulates a carbon balance close to neutral at Palace Leas (5 ± 2 g C m^{-2} yr^{-1}), and a small source at Park Grass (23 ± 10 g C m^{-2} yr^{-1}), which are both within the range of observations variation across different plots (–15 to 78 g C m^{-2} yr^{-1} at Palace Leas, and –16 to 25 g C m^{-2} yr^{-1} at Park Grass). The comparison with the long-term grassland experimental sites demonstrates the capability of our model in reproducing the observed soil carbon dynamics under different land cover change trajectories (the site in the United States[54]) and management histories (the two sites in the United Kingdom[55]). This result, to some extent, justified the use of our model to simulate grassland carbon balance under land cover change and management change.

In summary, the national/regional grassland carbon budgets estimated in this study are generally comparable with estimates from the literature obtained using various methods, although our estimates are not always consistent with the observed soil carbon change from inventories. It should be kept in mind that: i) most of the SOC change measurements were conducted for topsoil (0 to around 0.3 m depth), while our model simulated that of the whole soil column; and ii) though wild fire and water erosion are considered, some soil carbon loss pathways such as residue burning, and dissolved organic carbon are not taken into account by our model. The above factors could bring bias into our comparison. Based on the sensitive validations above, we conclude that our model is not likely to greatly overestimate the carbon sink of grasslands globally. It gives us sufficient confidence in the simulated historical carbon

* For the virgin grassland site at the *Kellogg Biological Station*'s Long-Term Ecological Research (LTER) site, southwest Michigan, United States, oak-hickory forest (represented as temperate broadleaves deciduous forest in ORCHIDEE) was cleared for grassland in 1960, and the grassland was mown once per year since then. For the *Palace Leas (PL)* meadow hay plots located at northeast England, United Kingdom, the meadow was established in 1897. Hay is cut and removed from the plots every year and the aftermath is grazed by sheep or cattle until autumn. For the *Park Grass (PG)* continuous hay experiment located at southeast of England, United Kingdom, the plots were established in 1856 on a permanent pasture. Between 1856 and 1875, the plots were cut for hay once per year and the aftermath was grazed by sheep penned on each plot to prevent nutrient transfers, but since 1875 there has been no grazing and a second hay cut has been taken.

budgets in grasslands worldwide (with a ±46% uncertainty estimated; see Methods and Supplementary Table 2), and its cooling effect.

The grassland CH_4 emissions from domestic livestock were 43 ± 8 Tg CH_4 yr^{-1} in 2003, similar to the estimate of 44 Tg CH_4 yr^{-1} derived from grassland-derived feed by ref[56]. Most studies estimate CH_4 emissions from the livestock sector as a whole (including those from enteric fermentation and manure management, and from livestock fed with all kinds of feedstuff) using different tiers of IPCC guidelines (Tier 1 or Tier 2). The emissions range from 96 to 118 Tg CH_4 yr^{-1} in 2010 [57-62]. Our estimate is that grassland CH_4 emissions (49 ± 10 Tg CH_4 yr^{-1}) account for 42 – 51% of those from the whole livestock sector. It is noteworthy that a study using Tier 3 of the IPCC guidelines estimated much lower livestock CH_4 emissions in 2000 (52 Tg CH_4 yr^{-1}; ref[63]). The grassland CH_4 emissions from wild grazers are derived initially from a combination of wild animal CH_4 emissions (grazer + browser; ref [64]) and diet information[65] for the reference year 1800 (see Supplementary Methods 1, Section *"Reconstructing the history of wild grazer density and grazed area"* for details). We estimated that 11.8 Tg CH_4 were emitted by wild grazers in 1800, comprising nearly half of the total wild mammal's CH_4 emissions from ref [64]. The estimated grassland CH_4 emissions from wild grazers were 1.8 ± 0.4 Tg CH_4 in 2006, comprising only 14% of the CH_4 from both grazers and browsers estimated by ref[64] (13 Tg CH_4). The difference mainly comes from the resolution of the Anthromes products[66,67] used to calculate wild animal extirpation. Ref[64] used a coarse urbanization index from the Anthromes products[66] to calculate the regional extirpation of wild mammals and the consequent CH_4 emission reductions. Here, we use gridded Anthromes products[66,67] to reconstruct the gridded history of wild grazers and their CH_4 emissions, which should better represent the spatial pattern of the wild grazers extirpation. Large ranges of the CH_4 from both grazers and browsers (2–15 Tg CH_4 yr^{-1}) were found from different studies[68]. Our estimate, from wild grazers only, lies in the lower part of this range[68], and is similar to the value in a recent study using various emission-body mass models[69].

Current global estimates of N_2O emissions from grasslands mainly focus on those from domestic livestock. To be comparable with ref [59,62,63,70], we exclude N_2O emissions due to atmospheric nitrogen deposition over grasslands and from wild grazers. Our estimates of the global grassland N_2O emissions from domestic livestock are in agreement with the N_2O emission from manure left on pasture estimated by ref[62] (Supplementary Table 2) for 1961 and 2010 (a difference of 0.2 Tg N_2O yr^{-1}). Our estimate is comparable with those from the literature[59,63,70], and is also consistent with that from a process-based model which includes a

representation of soil nitrogen dynamics associated with N_2O emissions[71]. Spatially, our estimates are in good agreement with the country-scale estimates from ref[62] (r = 0.81, $p < 0.0001$; country-scale grassland N_2O emissions from this study and from FAOSTAT are first averaged for the period 1961–2012, then log transformed and compared).

In summary, non-CO_2 emissions estimated in this study are consistent with all 5 inventories we compiled with emissions from grassland (one for grassland CH_4 emissions[56], and four for grassland N_2O emissions[59,62,63,70]). They are all within the uncertainty range of our estimates.

Supplementary Discussion 2. Vertical profiles of the simulated grassland soil carbon change

In ORCHIDEE-MICT [72], the model version that ORCHIDEE-GM v3.2 is built on, the three soil carbon pools (active, slow and passive) share a common 32-layer discretization scheme with soil temperature, to a maximum depth of 38 m (such a large soil depth is needed only when simulating permafrost C profiles and cryoturbation burial of C with depth). Here, our model simulated the SOC changes of the whole soil column to a depth of 2 m, rather than 38 m, because we do not consider cryoturbation. New carbon input to the soil pools from decomposed litter (separated into metabolic and structural) is partitioned with depth using an exponential function which corresponds to the prescribed root profile for each vegetation type (i.e., plant functional type, PFT). Decomposition of soil carbon is calculated in each layer, as a function of the fraction of each pool, using a base turnover rate, modified by soil temperature, moisture, and texture [73,74]. Vertical mixing of soil carbon due to bioturbation is accounted for by a diffusion term in the soil carbon equation. Ref [72] gives a detailed description of this soil carbon discretization, and a validation of the spatial pattern and vertical profiles of soil organic carbon over high latitude regions (see their Section 9.2 and Figure 21).

Ref [75] used stable carbon isotope signature data from whole-soil profiles to derive the age distribution curve with depth for 55 tropical grassland and forest soil profiles (see their Fig. 2). We conducted a series of simulations to derive the age distribution of SOC in the soil profile, and validate the age distribution against those shown in ref [75]. In these simulations, we stopped soil carbon input (i.e., vegetation litter in the model) for 3, 10, 30, 50, and 100 years before the year 2010, respectively. The SOC differences in each layer at the end of 2010 between these test simulations and the reference simulation (i.e., with continuous soil carbon input) can then

be used to determine the fraction of SOC with age lower than 3, 10, 30, 50, and 100 years, respectively as a function of depth. Supplementary Figure 15 shows the simulated carbon age distribution over the 32 tropical grassland sites used by ref [75] (in their Figure 2). The model was run for each 0.5 × 0.5 degree grid cell covering the locations of the 32 sites. For comparability with ref [75], the simulated soil carbon profile in the top 1 m of the soil was used. We find a decrease in the simulated new carbon fraction with increasing soil depth, as seen in the observations. The modelled new carbon fraction is lower than that fitted to the observations by a bi-exponential regression with age (as in ref [75]) for 0 and 10 cm depths, but higher than that for 20, 30, and 40 cm depths. We simulated that carbon of age less than 50 years represents 54 ± 9% of top-soil carbon (0-30 cm) and 22 ± 6% of deep carbon (30-100 cm), comparable to, although a little higher than, that calculated by ref [75] (45% of topsoil carbon and 13% of deep carbon; see their Fig. 2). The modelled soil carbon age distributions are comparable with those from meta-analysis based on isotope observations of soil profiles.

To validate the simulated distribution of the "recent" carbon that has been transferred from the atmosphere to soil organic matter since 1965 against "bomb-radiocarbon" observation-based estimates from ref [75], we conducted another simulation with no soil carbon input after 1965. The SOC differences in each layer at the end of 2012 between this simulation and the reference simulation (i.e., with soil carbon input) can then be used to show the impact of carbon incorporated into the soil since 1965. Here, model results were extracted over the locations of all grassland sites used by ref [75]. Supplementary Figure 16 shows the depth distribution of incorporated "recent" carbon since 1965 as a proportion of the total carbon incorporated in the top meter. The simulated depth distribution is similar to that for grasslands presented in the Fig. 3 of ref [75]. In particular, we simulated that around 84% [with 95% confidence interval of 82-86%] of the SOC incorporation since 1965 was in the topsoil (0-30 cm). The values are well within the range of observations. The modelled values of recent soil carbon incorporation (since 1965) between topsoil and subsoil are comparable with those from radiocarbon observations of soil profiles. The spatial pattern (Supplementary Figure 16) shows that around 80% of "recent" carbon since 1965 is simulated to be incorporated in topsoil over tropical and temperate grasslands, while this fraction is lower for grasslands in permafrost area such as high latitude regions of Siberia and North America and the high-altitude region of the Qinghai-Tibetan plateau. This is probably due to the fact that the longer freezing period of subsoil, compared to topsoil, in permafrost regions, causes lower soil heterotrophic respiration. Thus, more of the "recent" carbon incorporated since 1965 is left in the subsoil rather than being mixed into the

topsoil.

Supplementary Discussion 3. Model's responses to changes in grassland management

Here, we discuss the ability of the model to capture the historical dynamics of grassland management including overgrazing, associated degradation and their effect on the carbon balance of grasslands.

First, we compared the contemporary distribution of grassland productivity decrease (an indicator of degradation) estimated by our model and that detected by remote-sensing NDVI data during the period 1982-1991.

Second, by defining overgrazing in the model as when the NPP of a grazed grassland goes below a certain fraction of natural grassland NPP, we compared the modelled overgrazed area against independent degraded land area data from the Global Assessment of Soil Degradation (GLASOD) database[76].

Thirdly, we evaluated the modelled response of remaining un-grazed aboveground NPP (ANPP) carbon to increasing grazing intensities against local data from a meta-analysis [77].

Last, we evaluated the modelled response of soil organic carbon to different grazing intensities against data from several meta-analysis and review studies[77-79].

Modelled degradation matches decreasing NDVI

Loss of plant productivity is a characteristic of land degradation. One way to detect areas potentially under degradation, with decreasing productivity at the global scale, is to use remote-sensing proxies of productivity. The vegetation greenness (NDVI) is a good proxy for productivity that has been used in land degradation research for both regional studies[80,81] and global assessments[82-84]. NDVI data from AVHRR satellites cover the period from 1982 to the present, and have been complemented by other space-borne multi-spectral imaging instruments in the past two decades (e.g., SPOT vegetation and MODIS[9]). Given the fact that NDVI data do not differentiate between vegetation types in their footprint (0.0833 × 0.0833 degree resolution for AVHRR), for comparing simulated results with satellite NDVI trends, we used modelled mean leaf area index (LAI) of all plant functional types in each 0.5° grid cell, rather

than grassland LAI only. Forest dominated ecoregions, according to the IGBP land cover map from ref[85], were masked out.

Supplementary Figure 17 shows the (non-forest dominated) grid cells with significant ($p < 0.1$) decrease in the GIMMS3g AVHRR satellites NDVI products (at 0.0833 × 0.0833 degree resolution), and modelled LAI (at 0.5 × 0.5 degree resolution) during the period 1982-1991. To detect degradation, potentially due to land management practices like overgrazing, rather than to climate, grid cells with a concurrent decrease of productivity and precipitation were masked based on the observation that precipitation is the dominant factor controlling the productivity of grasslands, except in wet tundra biomes (blue in Supplementary Figure 17a and 17b). This procedure follows the approach used by ref[83] to detect degradation from NDVI. We found that our model can generally capture the satellite-based decreasing productivity trend (degradation) in the western United States, southern Brazil, Argentina, and Australia. Many of these areas (e.g., western United States; Supplementary Figure 18a) have high grazing intensity compared to surrounding areas but there are exceptions (e.g., southern Brazil and Australia). In central Asia and Sub-Saharan Africa, however, the observed decreasing NDVI from *degradation* was not well captured by the model. It should be kept in mind that i) we cannot separate the contributions of grassland and cropland in our comparison, and ii) the spatial resolution of the GIMMS3g NDVI (at 0.0833 × 0.0833 degree resolution) and the modelled LAI (at 0.5 × 0.5 degree resolution) are different, which prevents a detailed comparison being made for "pure" grasslands.

From this work we conclude that the model is capable of capturing the broad-scale patterns of the NDVI decrease attributed to degradation, according to the approach of ref[83], despite the simplified assumption used to reconstruct livestock density, and the lack of "migratory" grazing across grid cells.

Modelled overgrazing matches the GLASOD survey of land degradation

The Global Assessment of Soil Degradation (GLASOD) database[76] provides a reference data set of overgrazed areas based on regional surveys, often based on expert assessment and knowledge. Here, we compared the continental scale overgrazed area and its spatial pattern simulated by our model for the 1980s to the GLASOD data. Modelled output for the 1980s was used to be consistent with the time coverage of GLASOD. We classified a grassland grid cell as being *degraded by overgrazing* in the model if the modelled NPP of managed grassland

($NPP_{managed}$) is lower than 90% of the modelled NPP of natural grassland ($NPP_{natural}$) in the same grid cell. In addition, three overgrazing degradation levels are defined: light, moderate and heavy degradation if $NPP_{managed}$ is within 70-90%, within 50-70% and lower than 50% of $NPP_{natural}$ respectively. We found that the modelled land area *degraded by overgrazing* is comparable with the degraded areas from GLASOD globally[76] (adapted from Table 2 and 3 of ref[78]; Supplementary Table 6 and Supplementary Figure 19).

At the continental scale, we simulated a higher overgrazed area than GLASOD in Africa (+22%), North America (+111%) and South America (+57%), but a lower area in Australia (-41%) and Eurasia (-34%). GLASOD gives a higher fraction of moderate to strong degradation due to overgrazing than our model. However, it should be kept in mind that our level of overgrazing is defined according to productivity loss, while the GLASOD surveys focused on soil degradation. The spatial pattern of modelled overgrazed lands mainly agrees with that from the GLASOD database. The differences are mainly in the Great Plains of North America, the Middle East (Arabian peninsula, Iran, and Afghanistan), southwest Russia, and western and central Australia. It should be noted that the model does not reproduce the overgrazed area in Iran, Afghanistan, and central Australia, mainly because information on grass biomass consumption is not available for these regions[63] and thus no grazing is simulated by our model there (grazing intensity = 0; blank areas in Supplementary Figure 18). It should also be kept in mind that GLASOD is thought to be broadly accurate, but is less certain at any particular location[78].

Globally, in the historical simulations the simulated overgrazed area is found to have increased from 129 million ha in the 1860s to 318 million ha in the 2000s (Supplementary Figure 20). We found a small increase of overgrazed area in North America (+14 million ha) and in Australia (+13 million ha) from the 1860s to the 2000s. In Eurasia, the modelled overgrazed grassland increased by 25 million ha, during two periods of recent history: between the 1880s and the 1920s and between 1950s until the present. Overgrazed grassland area is simulated to have gradually increased in South America between the 1860s and the 1980s, with a fast expansion in the past two decades. Africa is simulated to have tripled its overgrazed area from 40 million ha in the 1860s to 122 million ha in the 2000s. It should be kept in mind that 1) the spatial distribution of ruminants is kept constant across history in the reconstruction used to force ORCHIDEE-GM (the gridded livestock distribution in the world from the year 2005, GLW2[86]), 2) changes in grazing stocking density (an input to the model) and the overgrazed area simulated by our model are mainly driven by historical changes in livestock numbers at

the country/regional level, 3) sub-grid (below the scale of a 0.5° grid cell) heterogeneity of stocking density is ignored in the model, i.e. all areas in the real world that have been fenced to exclude grazing, conservation areas, inaccessible areas, and their historical changes, due e.g. to policies like the grazing act in the United States, or to changes in land use ownership and grazing regulations, and 4) cross grid cell migratory motion of livestock is ignored in the model.

We conclude that our simulations generally capture areas classified as overgrazed in the GLASOD data set during the 1980s. This gives us confidence that our simplified treatment of management provides a rather realistic simulation of degradation areas.

Modelled (over)-grazing decreases productivity like observed

ORCHIDEE-GM v3.2 explicitly simulates the following effects of grassland management on productivity: 1) harvest and grazing tend to reduce leaf biomass and LAI; 2) harvest and grazing also reduce leaf age, and younger leaves have higher maximum photosynthetic capacity (Vc_{max}); 3) defoliation occurs due to livestock trampling; 4) manure and mineral fertilization increase the nitrogen availability and stimulate plant growth in the model via an empirical response function [87]; 5) strong defoliation (e.g., through overgrazing) could reduce carbon reserve accumulation (e.g., soil seed stock, rhizomes; represented as carbon reserve in the model), and further affect the regrowth at the beginning of next growing season.

We adopted grazing intensity (GI) from ref[77] as an indicator of management intensity: GI is defined as the ratio of total harvested and grazed carbon to total aboveground NPP (ANPP) over a managed grassland. Supplementary Figure 18b shows the spatial pattern of GI over managed grassland in 2000s from our historical simulation *E4* in this study (see simulations list in Supplementary Table 1). High GI is simulated in part of the western United States, the southern edge of Amazonia, southeast Argentina, Europe, northern and eastern Africa, India, Mongolia, and northern and southwestern China.

We show in Supplementary Figure 21 that our model captures the observation-based negative relationship between remaining ANPP and grazing intensity. The regressions diagnosed from the model output (an emerging output from the set of complex model equations) are similar to the ones found in the meta-analysis of ref[77] (see their Fig. 4), which was derived from reported numbers of animal unit months at different sites, and the carrying capacity calculated from NPP simulated by the Miami empirical model[88,89]. The regressions shown in Supplementary Figure 21 are for different climatic zones according to ref[90]. We simulated steeper (negative) slopes of

regressions in managed grasslands over moist warm climate zones, and flatter slopes over dry warm climate zones.

Based on this test, we conclude that the model is consistent with the most up to date meta-analysis of site data for the decrease of remaining ANPP with increased grazing, which gives us confidence that the input to SOC pools as a function of grazing is realistic.

Modelled (over)-grazing decreases soil organic carbon (SOC) as observed

The effects of grazing / harvest on net primary productivity impacts soil organic carbon (SOC) depending on the soil input (remaining NPP left as litter). However, our model does not represent several other processes of potential importance for SOC balance including, but not limited to: 1) overgrazing alters soil structure and has impacts on processes like water infiltration; 2) overgrazing depletes soil nutrient storage causing permanent degradation, 3) degradation from selective grazing by livestock, which may change species composition and cause e.g. woody encroachment. Therefore, it is crucial to evaluate the modelled responses of the grassland carbon balance (i.e., soil organic carbon change) to different management intensities.

We compared the modelled responses of SOC to different grazing intensities against data from the literature. Several studies have collected data focused on in-situ grassland plot to investigate the response of SOC storage to grazing and grazing intensities [77-79]. To make the comparison with model output, we conducted a set of global gridded simulations with different levels of prescribed grazing intensities. Although grazing intensity can be prescribed *quantitatively* in our simulations, it is usually only *qualitatively* reported in literature[77]. Thus we need to relate grazing intensities in the simulation experiments to site-level qualitative information. For example, ref[78], following ref[91], defined *heavy grazing* as a removal of approximately 50% of aboveground annual production (i.e., ANPP), which is 33% higher than the US Natural Resources Conservation Service recommendation. The above definition implies a 37.5% removal under *moderate grazing*. Ref[77] calculated GI based on numbers of animal unit months from literature and ANPP from the Miami model [88,89], and defined four classes of intensity as *low* (GI = 0-0.33), *medium* (GI = 0.33-0.66), *high* (GI = 0.66-1.0), and *overgrazed* (GI >= 1.0). Here, we chose grazing intensities of 25%, 37.5%, 50%, and 75% in the model to correspond to *light*, *moderate*, *heavy*, and *over grazing* respectively. Starting from the vegetation and SOC status in 1980 from the historical simulation *E4*, we conducted four test experiments with the

above four grazing intensities, along with a control run without grazing, for 20 years. The simulations were driven by gridded climate fields and CO_2 concentration data from 1981-2000 without any land cover changes. We chose the period 1981-2000 due to the fact that a majority of the grazing experiments in the meta-analysis were conducted within this period [77-79].

We did not compare the modelled response with the observations for the exact location of the in-situ measurements because the local conditions and management history are not supposed to be reproduced in our simulations. Instead, we compared the responses from the meta-analysis with model results from all grid cells with GI > 0 in Supplementary Figure 18b (i.e., with non-zero livestock distribution as in the gridded livestock in the world data set, GLW2[86]) and non-zero grass biomass use (as in the data set of ref[63]).

Compared to the control simulation without grazing, we found that increasing GI tends to reduce SOC (Supplementary Figure 22a), by an amount similar to that found in observations, see for instance Fig. 5 of ref[79]. The SOC response from our simulations also reproduces the range in the observations. The simulated SOC decrease, in response to increased grazing intensity, is largest in regions with annual mean precipitation >= 1000 mm (moist; Supplementary Figure 22b) and / or with annual mean temperature >= 10°C (warm; Supplementary Figure 22c and Supplementary Figure 23). According to our simulation, grasslands in South America and Africa are most sensitive to increased grazing intensity, because the SOC turnover time in moist/warm conditions is short, making SOC sensitive to the changes in soil input (i.e., remaining NPP after grazing). In dry/cold regions, slower decomposition allows larger SOC accumulation than in moist/warm regions, and a 20-year decrease in soil input does not much change the SOC content in percentage terms. In the meta-analysis of ref[79], however, the largest SOC decrease with intensive grazing was found in grasslands with annual mean precipitation <= 600 mm. Ref[77] (see their Fig. 5) also found the largest SOC decrease in response to grazing over such dry warm regions. This difference indicates that our model may underestimate the SOC response to grazing in dry warm regions.

Ref[78] collated the SOC of 22 paired plots under heavy and moderate grazing intensity respectively, and calculated the potential carbon sequestration rate as the difference in SOC between moderately and heavily grazed treatments divided by the duration of treatment. Similarly, we calculated the potential carbon sequestration rate as the difference in SOC at the end of year 2000 between simulation with *moderate* (GI = 37.5%) and *heavy* (GI = 50%) grazing intensity divided by 20 years of simulation. We simulated an increase in the potential

carbon sequestration rate with increasing annual mean precipitation, consistent with the finding by ref[78]. However, the average carbon sequestration rate during the first 20 years, 9.7 ± 8.8 g C m^{-2} yr^{-1} (see box plot in Supplementary Figure 24 for North America), is lower than the rate (18 g C m^{-2} yr^{-1}) estimated by ref[78].

From this test, we conclude that the SOC decrease in the model in response to increased grazing intensity is reasonable, although perhaps the model overestimates the negative sensitivity to grazing in warm and wet regions characterized by a mean rainfall above 1000 mm yr^{-1} and underestimates the sensitivity to grazing in warm and dry regions.

In summary, from the four complementary evaluation tests in Supplementary Discussion 3 detailed above, the grazing parameterization of our model can reproduce overgrazing diagnosed from NDVI, degraded grassland regions (GLASOD) and observed negative relationships between remaining NPP and increased grazing, and between SOC and increased grazing.

There are some regions where the model performances are weaker though (central Asia). Regional historical drivers of over-grazing (e.g. the homestead act and grazing act in the United States, land use conflicts between pastoral and agricultural communities in Africa) and feedbacks on degradation (e.g. increased wind erosion and dust, shrub encroachment and other desertification processes) are not represented in the global model for dry regions.

For example, the US Great Plains experienced widespread overgrazing before the 1960s followed by a partial recovery[92], and our simulations may not account well for this overgrazing history (Fig. R3). We simulated relatively low GI in the region (GI < 0.2), driven by our low reconstructed grazing stocking rate (Supplementary Figure 18). In reality, there could have been hotspots with high grazing stocking rates and also changes in livestock distribution in the history of that region [92]. But it is beyond our capacity (and the purpose of this "global" paper) to reconstruct a better density map, because, as far as we know, large scale quantitative information on historical distribution of ruminants is not available.

Alternatively, to investigate the potential carbon losses and gains due to plausible management changes in North America, we conducted a sensitivity simulation with prescribed grazing intensity of 50% (*heavy grazing*) during the period 1900-1960 and of 37.5% (*moderate grazing*) after 1960 (hereafter referred to as simulation $E_{prescribe}$). Significant soil carbon losses over managed grassland before the 1960s are simulated with the arbitrarily prescribed *heavy grazing* intensity, while marginal carbon sinks over managed grasslands are found after 1960s with

arbitrarily *moderate grazing* intensity (Supplementary Figure 25b). In simulation $E_{prescribe}$, managed grasslands are simulated to be a net source of +0.02 Gt CO_2 yr^{-1} since 1900 (Supplementary Figure 25b), in contrast to the net sink of -0.12 Gt CO_2 yr^{-1} since 1900 in simulation *E4* (Supplementary Figure 25a; i.e., the original simulation with the default reconstructed grazing intensity; Supplementary Table 1). On the other hand, the high grazing intensity scenario results in less managed grassland to fulfil the reconstructed grass biomass use (Supplementary Figure 25d compared to Supplementary Figure 25c). The larger unmanaged grasslands in simulation $E_{prescribe}$ tend to sequestrate more carbon (sink of -0.36 Gt CO_2 yr^{-1} over 1900-2012) than that in simulation *E4* (sink of -0.25 Gt CO_2 yr^{-1} over 1900-2012). For the carbon budget over all the grasslands in North America, a weaker carbon sink is obtained in simulation $E_{prescribe}$ during the period 1900s to the 1950s (-0.15 Gt CO_2 yr^{-1}), compared to that from the control simulation (-0.20 Gt CO_2 yr^{-1}). Since 1960, however, the carbon budgets of the two simulations are similar (-0.57 Gt CO_2 yr^{-1} in both simulations). The resulting net climate effect of grasslands of North America in 2012 is very close for simulations *E4* (-55 ± 26 mW) and $E_{prescribe}$ (-52 ± 24 mW), meaning that, according to our simulations, distant past management changes (e.g., before the 1950s in North America) have a small legacy impact on the contemporary carbon balance of grasslands but a significant impact in the 20th century period when past management was assumed to be more intense.

Supplementary Discussion 4. Attribution of the simulated grassland carbon balance to different drivers

To disentangle the effects of management and environmental drivers, we conducted a series of simulations with one factor fixed at a time to attribute the contribution of environmental drivers 1) climate change, 2) rising CO_2, 3) atmospheric nitrogen deposition, and management drivers 4) land-use change related to grassland area change transitions, 5) changes in livestock densities, human input of carbon and nitrogen from manure, and nitrogen from mineral fertilizers, and human extirpation of wild grazers (Supplementary Table 7). The contribution of each environmental driver has the net effect to enhance grassland carbon storage during the period 1860-2012. The increase of CO_2 by 105 ppm since 1860 has the largest contribution (85% of the net cumulative sink of all the drivers including land use change). The contribution of climate change alone is positive (sink) in some regions but negative in others, although it is globally positive (22%). The contribution of N deposition is comparable, being a global net sink (24%

of the total cumulative sink from all drivers). Human-caused changes of livestock densities and extirpation of wild grazers accounts for 39% of the global cumulative sink including all drivers. In contrast, land use change related to grassland conversion (forest to pasture and grassland to cropland) is responsible for a large cumulative loss of carbon to the atmosphere, which offsets 141% of the cumulative sink. A residual sink of 69% is due to nonlinear interactions of all the drivers (Supplementary Figure 5a).

Increasing CO_2 and atmospheric nitrogen deposition have a consistent positive effect on the grassland CO_2 sink in all regions due to their positive effect on grassland productivity. Land-use changes related to deforestation to pasture and conversion of grassland to cropland are net sources in all the regions where they occurred. On the other hand, the effect of individual drivers can vary in different regions. Climate change has a positive effect on grassland carbon storage in North America, the Russian Federation, East and Southeast Asia, and Oceania, almost no effect in Europe and South Asia, and a negative effect in the Near East and North Africa, Latin America and the Caribbean, and Sub-Saharan Africa. The effect of climate change also has decadal variations due to climate variability in different regions and over time (Supplementary Figure 5b). For example, the strong grassland carbon sink in the 1970s is derived from the strong sink in Oceania and Latin America, which is probably caused by the favourable climate for grass growth and thus increased soil carbon input. Grassland management (with an increase or decrease of livestock numbers) and the extirpation of wild grazers, have a positive effect on the grassland CO_2 sink in most regions, while they have a negative effect in Latin America and Caribbean. At the global scale, grassland management and the extirpation of wild grazers have a positive effect on the grassland CO_2 sink over history, but the effect has become neutral in the past decade.

Supplementary Figure 6 shows the spatial distribution of the carbon balance over the past three decades. Net SOC sinks are located in the temperate North American and Eurasian grasslands, especially in Europe where livestock density has decreased significantly in the past two decades. Conversely, net carbon sources are found in South America and Africa, partly due to deforestation for pasture and increasing livestock numbers (thus higher grass biomass consumption and less litter left as soil input) in those regions. We further investigated the effects of climate change, increasing CO_2, and atmospheric nitrogen deposition since 1860 on soil organic carbon content change in topsoil (0-30 cm) and subsoil (30-100 cm). The results are shown in Supplementary Figure 26. For each driver, our model gives similar spatial patterns of the impacts on topsoil and subsoil SOC. These drivers, in general, have stronger impacts on

topsoil than subsoil SOC. Climate change (mainly temperature and precipitation) has positive or negative effects on SOC across the globe. Increasing CO_2 has a positive effect on grassland SOC all over the world, with the exception of the tropics where grasslands are mainly converted tropical forests. This result indicates that, in the model, the SOC of tropical forest has a larger positive response to increasing CO_2 than the SOC of tropical grassland, and that conversion of tropical forest to grassland, under a simulation with increasing CO_2, leads to a larger decrease in SOC than a simulation with stable CO_2 levels. Increasing atmospheric nitrogen deposition also has a positive effect on grassland SOC, especially over regions with relatively high deposition rates, such as eastern North America, Europe, and China.

Supplementary Discussion 5. Impact of erosion on the grassland carbon budget

We account for grassland carbon fluxes due to water erosion by using estimates from a global gridded erosion module emulated in the carbon cycle of ORCHIDEE-MICT by an adjusted version of the Revised Universal Soil Loss Equation (Adj.RUSLE) model [93]. The emulator comprises grassland soil carbon dynamics, erosion removal fluxes, and the compensatory soil sink process from NPP inputs. The soil carbon dynamics scheme of the emulator is derived from the ORCHIDEE-MICT model, while Adj.RUSLE is used to calculate the annual average soil erosion rate for each biome in each grid cell. The erosion factors in the Adj.RUSLE model are modified so as to be compatible with coarse resolution environmental input data. The model is calibrated to be applicable at the large spatial scale and has been validated at the global scale for different land uses[94,95].

Water erosion decreases soil organic carbon, but the removal of soil organic carbon leads to a decrease in soil respiration. With the assumption that carbon input through NPP will not change, i.e. there is no feedback of erosion on a loss of NPP through degraded soil physical properties and fertility, erosion can result in a net loss or an enhanced carbon sequestration when NPP inputs compensate for carbon removed laterally by erosion. Therefore, the net carbon fluxes caused by water erosion include both gross carbon erosion removal and the extra sink due to reduced soil respiration compared to NPP derived inputs. The soil erosion and extra sink from ref[93] is based on a ORCHIDEE-MICT version similar to the one used in this study (but without accounting for grassland management) and with identical land cover change history as used here[96]. Therefore, we used the gridded gross carbon erosion removal fluxes and the extra sink

over grassland for the period 1860-2005 from simulation S1 of ref[93] directly to estimate net carbon fluxes caused by water erosion over grassland. For the period 2006-2012, fluxes with values equal to the mean values during the period of 2000-2005 are applied. In addition, to account for the uncertainty in water erosion, the minimum and maximum soil erosion scenarios from ref[94] are applied to calculate the range of erosion fluxes.

Globally, we found that gross soil carbon losses due to water erosion in grasslands increased from 204 [165 – 208] Tg C yr^{-1} in 1860s to 287 [242 – 380] Tg C yr^{-1} in 1990s (values in the bracket are estimates obtained by using the minimum and maximum soil erosion rates; Supplementary Figure 25a). Carbon lost by this removal process was assumed to be respired as CO_2 to the atmosphere, which is a conservative estimate of the net carbon loss from soils due to water erosion (see below). In the meantime, the erosion-induced soil carbon sink due to a soil respiration reduction increased from 196 [156 – 266] Tg C yr^{-1} in 1860s to 223 [181 – 303] Tg C yr^{-1} in 1990s (Supplementary Figure 27b).

Overall, we found that net carbon fluxes induced by water erosion over grasslands increased from a marginal source of 8 [8 – 15] Tg C yr^{-1} in 1860s to a significant source of 63 [60 – 76] Tg C yr^{-1} in 1990s (Supplementary Figure 27c). During the 1990s, the strongest water erosion and resulting net carbon source are simulated over regions with high precipitation (e.g., tropical regions, southeast Asia, and northwest North America) or/and high rainfall erosivity[97], or/and high slope steepness (e.g., southwest China, and the Alps; Supplementary Figure 28).

Wind erosion was not accounted for as, to our knowledge, no global dataset is available. In addition, overgrazing and drought tend to reduce vegetation coverage and enhance wind and/or water erosion rates. However, there is no global parameterization of the above effects as far as we know, which prevent us taking them into account. Nevertheless, the grassland net carbon fluxes due to erosion used in this study could potentially be underestimated because we neglect wind erosion (e.g., ref [98]) and the potential enhanced water erosion from overgrazing and drought. Although it is beyond the scope of the grassland carbon balance focus of this study, it should be noted that not all of the eroded soil carbon is released as CO_2, and part of the carbon is redeposited and can be buried for long periods in alluvial and colluvial reservoirs [99]. For example, previous studies have shown that up to 80% of the eroded soil and carbon can be redeposited, with about 10% to 20% of the eroded carbon being sequestered[100,101].

Supplementary Discussion 6. Effect of grassland management on the simulated fuel loads, burned area and fire emissions

To account for the impact of fire on the grassland carbon balance and the interaction between grassland management and fire, the SPITIFIRE model, which was part of another version of the ORCHIDEE model, was included in the version of ORCHIDEE-GM v3.2 used in this study [72,102,103]. The SPITFIRE fire module explicitly simulates open vegetation fires, including fuel load impacts on fire propagation and fire impacts on grassland and woodland dynamics [102,103]. Here, we first compared the simulated burned area and fire CO_2 emissions against observation-derived estimates and against an ensemble of state-of-the-art global fire models that are included in the fire model inter-comparison project (FireMIP [104]). We then investigated the effects of grassland management on burned area, CO_2 emissions, and fuel loads.

The simulated global burned area during the period 1997-2012 (4.9 ± 0.2 million km^2; without accounting for grassland management) is found to be well within the range of three satellite-based observation data sets (4.9 ± 0.2 million km^2 in GFED4s data set; 4.0 ± 0.3 million km^2 in ESA-CCI data set; and 3.6 ± 0.2 million km^2 in GFED4 data set) and those from FireMIP models (ranging from 3.5 ± 0.3 to 5.4 ± 0.3 million km^2 [105]; Supplementary Figure 29a). The simulated global fire emissions are 2.0 ± 0.1 Pg C yr^{-1} during 1997-2012 without accounting for grassland management, which is comparable to the GFED4s data set using satellite burned area and fuel load from the diagnostic CASA model (2.2 [1.8 – 3.0] Pg C yr^{-1}; numbers in brackets give minimum and maximum estimates) where grassland management effects on fire emissions are not considered.

Grassland management significantly decreases fuel loads (dominated by aboveground litter in our model) by 1.4 Pg C in 1900s (Supplementary Figure 29c). Following the increase in ruminant numbers, the expansion of managed grassland, and the increase in management intensity over the 20th century (Supplementary Figure 2), we simulate a decrease in fuel loads caused by grassland management reaching 1.8 Pg C in the 2000s, compared to the simulation without grassland management. The lower fuel loads further reduced burned area by 0.2 million km^2 (Supplementary Figure 29a) and decrease associated fire emissions by 0.1 Pg C yr^{-1} in the 2000s (Supplementary Figure 29b). Thus, in our simulation, grassland management significantly decreased burned area over most of the managed grassland, especially in Sub-Saharan Africa (decrease by 0.13 million km^2 (7%) in the 2000s) and South America (decrease by 0.03 million km^2 (5%) in the 2000s). In a few regions such as the northern Great Plains in

North America and southern France, the extensive grazing increases fuel loads and slightly increases burned area (Supplementary Figure 30b). We further investigated the impact of grassland management on fuel loads under different grazing intensities (mean values during the period 1991-2010). As expected, in general, fuel loads decrease along with increasing grazing intensity (Supplementary Figure 23d).

Furthermore, we investigated the modelled trend of burned area with accounting for grassland management (Supplementary Figure 30c) and the contribution due to the changes of grassland management intensity during the period 2000-2012 (Supplementary Figure 30d). To attribute the effects of management intensification, we conducted a factorial simulation with grassland management intensity fixed at the year 2000 level for the period 2000-2012 ($E_{fixintensity}$). The difference in the trends of burned area between the default simulation *E4* and $E_{fixintensity}$ is the contribution from management intensification. Our results show that recent intensification of grassland management led to a decreasing burned area in some regions of Sub-Saharan Africa and South America (Supplementary Figure 30d). We found that grassland management intensification, caused by the increase in livestock numbers, contributed 35% of the overall simulated downward trend of burned area in northern Africa (latitude > 0°N; trend of -1.38% yr^{-2} for default simulation *E4* and for -0.89% yr^{-2} for simulation $E_{fixintensity}$). The effect is much larger than the contribution from cropland expansion (20% for the period 2001-2012 [106]). In South America, 60% of the overall simulated downward trend of burned area can be attributed to the grassland management intensification (trend of -0.97% yr^{-2} for default simulation *E4* and for -0.38% yr^{-2} for simulation $E_{fixintensity}$).

In summary, our model can effectively capture the impact of fire on the carbon balance of grasslands and the impact of grassland management on fuel loads and the trends of burned area. Biomass burning not only emits CO_2, but can also release various components into the atmosphere including CH_4 and N_2O [107]. Here, we do not account for the fire related CH_4 and N_2O emissions, but the error induced by such omission is small, given the fact that CH_4 and N_2O emission accounts for 2.9% and 3.1% of the warming potential of the GHG emissions from savanna fires [108]. In addition, fire may alter the soil CO_2, CH_4 and N_2O emissions (e.g., ref[109]). Although these soil effluxes of CH_4 and N_2O are not simulated in our model, the impacts of fire on soil CO_2 efflux have been implicitly included, mainly through fire impacts on vegetation and its further impacts on the land surface and soil energy and water balance.

Supplementary Discussion 7. Grassland area, managed grassland area and management intensity

In the historical land cover maps[96] used in this study, the global grassland area changed from 51.7 million km^2 in 1860 to 49.7 million km^2 in 2012 (Supplementary Figure 2). The current grassland area used in this study is within the wide range given by the recent IPCC SRCCL [110], which indicates grass dominated ecosystem covering 37% (30 – 47%) of the global ice-free land surface (i.e., 48.1 (39-61.6) million km^2). The global managed grassland area estimated here increased from 8.5 million km^2 in 1860 to 16.5 million km^2 in 2012 (Supplementary Figure 2). The largest expansion of managed grassland is found in sub-Saharan Africa (+2.6 million km^2) and Latin America and the Caribbean (+2.0 million km^2), followed by the Near East and North Africa (+1.7 million km^2) and East and Southeast Asia (+1.2 million km^2). Besides the expansion of managed grassland area, the average grazing intensity of managed grassland (GI) was simulated to increase 60% globally from 0.10 to 0.16 (Supplementary Figure 31). Here, GI is an indicator representing management intensity[77], which is defined as the total harvested and grazed carbon as a ratio of total aboveground NPP (ANPP) over managed grassland. The largest value of GI and also the largest GI increase was simulated to be in South Asia, due to the high grass biomass demand and low grassland area. Over European managed grassland, GI increased continually since the 1860s, but dropped rapidly in the past two decades. A similar drop in GI was simulated in Russia, due to the significant decrease in livestock numbers in these two regions. A slight decrease in GI was also simulated in North America in the past three decades. A fast increase in GI was simulated over managed grassland in East and Southeast Asia in the past three decades, and in Latin America and the Caribbean in the past five decades. Supplementary Figure 32 shows the spatial distribution of grassland fraction in 2010, and managed grassland fraction, grazing stocking rate and grazing intensity (GI) in the 2000s obtained in this study. High GI values were simulated in part of the western United States, the southern edge of Amazonia, southeast Argentina, Europe, northern and eastern Africa, India, Mongolia, and northern China.

Supplementary Discussion 8. Effect of rising atmospheric CO_2 concentration on the simulated productivity

The rising CO_2 concentration is the main driver of the simulated grassland CO_2 sink in all regions. It has the dual effect of increasing leaf photosynthesis and reducing stomatal

conductance, thus indirectly increasing soil moisture in unsaturated soils. These effects increase water-use efficiency [111] and reduce the consumption of soil moisture by plant transpiration [112]. Experiments like Free-Air CO_2 Enrichment (FACE) have tested the effects of elevated CO_2 on a number of terrestrial ecosystems (usually between ambient CO_2 concentration and an elevated level of 475-600 ppm), and provided valuable data on the responses of the carbon cycle. However, trends and variability in temperature and precipitation, as well as nitrogen limitation, will all interact with the effects of elevated CO_2 to determine actual changes in productivity in response to CO_2 [112-116]. In addition, the response to the increasing CO_2 may be non-linear, which means the response to further CO_2 increase may not be fully comparable with the response in history. The strong interactions of the CO_2 fertilization effect with local conditions and the non-linear response prevent a direct comparison with our simulation which has a spatial resolution of 0.5 by 0.5°.

Instead, we compare the simulated response of plant productivity to increasing CO_2 during the historical period (i.e., CO_2 fertilization effect E_{co2}) to observation-based estimates for C3 plants from historical change of deuterium isotopomers in leaf herbarium samples [117] and for global (C3 and C4) vegetation to indirect evidence from carbonyl sulfide (COS) atmospheric ice-core observations [118]. Here, the CO_2 fertilization effect (E_{co2}) is defined by the ratio of GPP (g C m^{-2} yr^{-1}) under the current CO_2 concentration of 396 ppm (GPP_{396}) to that under a CO_2 concentration of 296 ppm (GPP_{296}). The CO_2 concentrations of 296 ppm and 396 ppm correspond to the tropospheric mixing ratio of CO_2 in year ~1900 and 2012 respectively, and are similar to the values used for estimating the response of GPP to a ~100 ppm CO_2 increase in refs [117] and [118]. It should be noted that the comparison is not only for grassland, but for natural biomes. The simulated GPP from grid cells of natural biomes averaged for the periods 1901-1905 and 2008-2012 were used as GPP_{296} and GPP_{396}, respectively. Here, natural biomes indicate grid cells: 1) with less than 20% of cropland fraction in both periods (i.e., without significant land use); 2) where biome (forest, grassland, cropland) changes by less than 20% of the grid cell between the two periods (i.e., without significant land-use change); and 3) with mean annual GPP higher than 100 g C m^{-2} yr^{-1} (i.e., not too sparse vegetation).

The modelled E_{co2} by ORCHIDEE-GM for natural biomes ranges between 1.13 and 1.46 for most regions (16th and 84th quantile regression, respectively; Supplementary Figure 33a) with mean E_{co2} of 1.23, slightly lower than the global E_{co2} based on long-term atmospheric carbonyl sulfide (COS) records of 1.26-1.36[118]. The modelled E_{co2} for C3 plants ranges between 1.15 and 1.47 for most regions (16th and 84th quantile regression, respectively; Supplementary Figure

33b). The mean E_{co2} of 1.24 is consistent with that from herbarium samples (E_{co2} = 1.25[117]). In summary, our simulation shows a comparable productivity response to the rising CO_2 in the past century compared to observation-based estimates,

Supplementary Discussion 9. The climate effect of grassland greenhouse gas fluxes

The global radiative forcing (RF) from CH_4 and N_2O emissions is 70 ± 15 mW m^{-2} (14 ± 3% of the global radiative forcing from CH_4 emissions) and 30 ± 10 mW m^{-2} (17 ± 6% of the global radiative forcing induced by N_2O emissions) respectively. Thus, they have a warming effect on climate. The net CO_2 sink of grassland makes a negative contribution (a cooling effect) of –194 ± 99 mW m^{-2}, offsetting 11 ± 5% of the current global radiative forcing induced by total anthropogenic CO_2 emissions including fossil fuel and land-use change emissions. In contrast, the land-use change emission related to grassland, including deforestation to pasture and conversion of grassland to cropland, has a warming effect of 108 ± 35 mW m^{-2} (7 ± 2% of the global radiative forcing induced by total anthropogenic CO_2 emissions). The climate effect of the grassland GHG fluxes accounts for not only the instantaneous effect of individual gases, but also their atmospheric lifetime[119,120]. The long lifetime of CO_2[121,122] indicates that early 20th century CO_2 sinks of grassland can still make a negative contribution to the current radiative forcing. Grassland-related surface albedo change induced by land-use change has only a marginal effect on climate (–3 ± 8 mW m^{-2}).

Since 1901, the cooling effect of carbon sinks in grassland soils worldwide has outweighed the warming effect of CH_4 and N_2O emissions, and partly offset the warming effect from land-use change emission related to grassland (Fig. 4b). This offsetting makes the net climate effect of global grasslands nearly neutral across the 20th century. This is remarkable, since grassland anthropogenic CH_4 and N_2O emissions increased by factors of 2.6 and 2.2 during that period, and large-scale deforestation to pasture (as in South America) took place after the 1930s. The absolute contributions of grasslands to global RF through CH_4 emissions have progressively increased since 1901. The grassland RF contribution through N_2O emissions shows a small increase between 1901 and 1960, and a strong increase afterwards. CH_4 emissions made a larger contribution to RF than N_2O emissions across the 20th century.

Supplementary Methods 1. Model and methods used for grassland greenhouse gas assessment

Model description

ORCHIDEE is a process-based ecosystem model developed for simulating carbon, water and energy fluxes in ecosystems, from site level to global scale[123-125]. ORCHIDEE-GM[126] is a version specifically developed to integrate the management of grassland[126]. The equations describing management in ORCHIDEE are derived from PaSim[127-130]. Accounting for management practices, such as mowing, livestock grazing and fertilizer application, on a daily basis, ORCHIDEE-GM is capable of simulating the dynamics of leaf area index, biomass, and carbon fluxes of managed grasslands. ORCHIDEE-GM v1 was evaluated, and some of its parameters calibrated, using eddy-covariance net ecosystem exchange and biomass measurements from 11 European grassland sites representative of a range of management practices. The model successfully simulates the net carbon budget of these managed grasslands[126]. Ref[131] added a parameterization of adaptive management through which farmers react to a climate-driven change to previous-year productivity. Though a full nitrogen cycle is not included in ORCHIDEE-GM, the positive effect of nitrogen fertilizers on grass photosynthesis rates, and thus on subsequent ecosystem productivity and carbon storage, is parameterized with an empirical function calibrated from literature estimates (v2.1[131]). ORCHIDEE-GM v2.1 was applied over Europe to calculate the spatial pattern, inter-annual variability, and the trends of potential productivity, i.e. the productivity that maximizes simulated livestock densities assuming an optimal management system in each grid cell[131]. This version was further used to simulate the net carbon budget, budget trends, and the GHG balance of European grasslands during the last five decades at a spatial resolution of 25 km[7]. Ref[132] recently updated the model to version 2.2 with the general parameterizations from ORCHIDEE Trunk.rev3623 (https://forge.ipsl.jussieu.fr/orchidee/browser/trunk#ORCHIDEE), and a new parameterization limiting grazing practices under specific conditions such as frost, snow cover and wet soil. ORCHIDEE-GM v2.2 was driven by projected future climate change to provide a European-wide assessment of the future changes in productivity and phenology of grassland, and their consequences for management intensity and the carbon balance.

At the global scale, ORCHIDEE-GM v3.1 is a development of v2.1, and includes a parameter adjustment for the C4 grassland biome and implements a specific strategy for wild herbivores [133]. Combining livestock production information, ORCHIDEE-GM v3.1 was applied to

reconstruct a series of global gridded maps containing a time-dependent history of grassland management intensity. These maps are model-dependent, and provide a unique opportunity for models with explicit representation of grassland management to make a more accurate estimate of global carbon and GHG budgets of grassland [133] – as we have here. In this study, ORCHIDEE-GM v3.1 has been updated with i) the parameterizations of v2.2 and ii) the general parameterizations from ORCHIDEE-MICT[72].rev5308 (https://forge.ipsl.jussieu.fr/orchidee/wiki/Branches/ORCHIDEE-MICT-IMBALANCE-P) to a new version named ORCHIDEE-GM v3.2. ORCHIDEE-MICT[72] is a version of ORCHIDEE with improved interactions between soil carbon, soil temperature and hydrology, and a fire module.

Model input

ORCHIDEE-GM v3.2 was run on a global grid using the 6-hourly CRU-NCEP v8[134,135] reconstructed climate data at 0.5° × 0.5° spatial resolution for the period 1901–2012. The climate data are identical to those used to drive TRENDY v6 models. The fields used as the climate input of the model are temperature, precipitation, specific humidity, solar radiation, wind speed, pressure, and longwave radiation. Global atmospheric CO_2 concentration, used to force the model, is prescribed from the combination of ice core records and atmospheric observations for 1860–2010[136]. The ESA CCI Land Cover product[137] for the year 2010 was used to produce the Plant Functional Type (PFT) map used in the ORCHIDEE model (Supplementary Table 4), following the methodology presented by ref[138,139]. An updated release of the historical land-use forcing data set LUHv2h (http://luh.umd.edu/data.shtml; updated from LUHv1[140]) was applied to this reference PFT map to constrain the land-cover changes of forest, grassland (combining pasture and natural grassland), and cropland during the period 1860–2010 using the backward method (BM3) following ref[96]. It is noteworthy that the cropland area in LUHv2h is constrained based on the HYDE3.2 data set[141]. Another environmental input is the atmospheric nitrogen deposition maps from the IGAC/ SPARC Chemistry-Climate Model Initiative (CCMI [142]). Dominant soil texture in each 0.5° × 0.5° grid cell used as model input is based on the 12 USDA texture classes provided at the 0.08° resolution from ref [143].

To simulate the GHG balance of grassland, we must use the historical grassland management intensity as a model input. We followed the same methods as in ref [133] to reconstruct the history of grassland management intensity for the period 1860–2012 combining gridded and regional

livestock production information with productivity from ORCHIDEE-GM v3.2. We extended the reconstructed historical grassland management intensity back to 1860 instead of 1901. To do so, we hindcasted management intensity to 1860 following changes in the regional population, assuming that domestic livestock production experienced the same rate of change as population during the period 1860–1900. Another difference comes from the historical land-cover change maps. The comparison between the historical land-cover change maps used here (described above) and in ref [133] is listed in Supplementary Table 4. The reconstructed historical maps on grassland management intensity (1860–2012) are the managed grassland area (Supplementary Figure 32b), with the fraction that is mown or grazed, and yearly maps of domestic grazing (Supplementary Figure 32c) and ruminant stocking density, and yearly maps of manure and fertilizer application rate over managed grassland (Supplementary Figure 33a). The methodology for the reconstruction is detailed in Supplementary Methods 1, Section *"Reconstructing the history of grassland management intensity"*.

In addition to domestic livestock, wild grazers also consume grass biomass and contribute to the GHG balance of grassland. Here, we combined wild animal species information[64], diet information[65], modelled natural grassland productivity and an anthropogenic biome classification system[66,67] to reconstruct the gridded history of wild grazer density and the wild and semi-natural grasslands occupied by these wild grazers (see Supplementary Methods 1, Section *"Reconstructing the history of wild grazer density and grazed area"* for details).

The managed grassland area and the fraction that is mown or grazed, and the wild and semi-natural grasslands occupied by wild grazers were incorporated into the historical land-cover change maps, which defined an enhanced historical land-cover map delineating grassland management types and wild grazer occupation (mown, grazed, natural, and wild). Input data consisting of nitrogen fertilizer application maps, including manure- and mineral-based nitrogen fertilizers, is as described in ref [133]. Atmospheric nitrogen deposition maps covering 1860–2012 were from the IGAC/SPARC Chemistry-Climate Model Initiative (CCMI) deposition fields[142].

Reconstructing the history of grassland management intensity

1. Domestic grazing-ruminant stock density

Gridded domestic grazing-ruminant stock density was constructed by combining the domestic ruminant stocking density, historical land-cover change maps and protected fraction. Assuming that all the ruminants in each grid-cell were grazing on the grassland within the same grid, we define the grazing-ruminant stocking density in grid-cell k in year m ($D_{grazing,m,k}$, unit: LU per ha of grassland area) as:

$$D_{grazing,m,k} = \frac{D_{m,k}}{f_{grass,m,k} \times f_{protected,k}} \tag{1}$$

where $D_{m,k}$ is the total domestic ruminant stocking density (unit: LU per ha of land area); $f_{grass,m,k}$ is the grassland fraction in grid-cell k in year m from a set of historic land-cover change maps described in Supplementary Methods 1, Section *"Model input"*; and $f_{protected,k}$ is the fraction of land area formally protected in grid-cell k derived from ref[144](https://geospatial.tnc.org/pages/data). We assumed that the protected area is excluded from grazing by domestic livestock and remains constant across history because we do not have any better information on the protected area history. Median $f_{protected,k}$ from the range provided by ref[144] was applied. To avoid unrealistic densities of ruminant grazing over grassland (which might cause grasses to die during the growing season), a maximum value of 5 LU ha^{-1} was set for the density map. In addition, a minimum grazing-ruminant density of 0.2 LU ha^{-1} was set, to avoid economically implausible stocking rates.

2. Domestic ruminant stocking density

The domestic ruminant stocking density maps originate from the Gridded Livestock of the World v2.0 (GLW v2.0[86]). GLW v2.0 provides gridded livestock density (unit: head per km^2) of cattle, sheep and goats for the year 2006. To obtain a more consistent and realistic ruminant stocking density, livestock species in GLW v2.0 were converted to livestock unit (LU) based on the calculation of the metabolisable energy (ME) requirement for each country. ME requirement, the amount of energy (MJ day^{-1}) an animal needs for maintenance and for activities such as lactation, and pregnancy, can be calculated based on meat (carcass weight) and milk yield following the IPCC Tier 2 algorithms[145] (IPCC, 2006 Vol 4, Chapter 10, Eqs. 10.3 to 10.13). One LU is defined as an average adult dairy cow producing 3000 kg milk annually, with live body weight of 600 kg [146] (with ME requirement of ca. 85 MJ day^{-1}, and with dry matter intake of ca. 18 kg daily). The conversion factor (F) for livestock category i (i.e., cattle, sheep or goats) in country j is calculated as:

$$F_{i,j} = \frac{ME_{head,i,j}}{ME_{LU}} \quad (2)$$

where ME_{LU} (unit: MJ yr^{-1} LU^{-1}) is the ME requirement by one LU; and $ME_{head,i,j}$ (unit: MJ yr^{-1} head^{-1}) is the ME requirement per head of livestock category i in country j, given by:

$$ME_{head,i,j} = \frac{ME_{i,j}}{N_{animal,i,j}} \quad (3)$$

where $N_{animal,i,j}$ (unit: head) is the total number (in head from FAOSTAT) of animals in livestock category i in country j; $ME_{i,j}$ (unit: MJ yr^{-1}) is the total ME requirement of livestock category i in country j, which includes the ME of animals for different production types (i.e., animals producing milk, slaughtered for meat, or animals neither producing milk nor slaughtered for meat; see Supporting Information Text S1 of ref[7] for details). To be consistent with the country-level livestock data used by GLW v2.0, statistical data [62] for the reference year 2006 were used to calculate the conversion factors.

For each grid-cell k in country j, the ruminant density of category i for the reference year 2006 ($D_{ref,I,j\ k}$) can be calculated as:

$$D_{ref,i,j,k} = D_{GLW,i,k} \times F_{i,j} \quad (4)$$

where $D_{GLW,i,k}$ is the original density of livestock category i in grid-cell k from GLW v2.0 data set (with original unit head per square km of suitable areas in livestock production systems [147]). To be consistent with the spatial resolution of climate forcings used to drive global vegetation models, the category-specific ruminant stocking density ($D_{GLW,i,\ k}$) was aggregated from the original resolution (about 1 × 1 km at the Equator) to 0.5° × 0.5° (about 50 × 50 km at the Equator) considering suitable areas in livestock production systems[147], and was then converted to the unit of LU per hectare of land area in each grid-cell.

Domestic ruminant numbers, and therefore stocking density, are continually changing from year-to-year as reported in FAOSTAT [62]. However, GLW v2.0 only provides livestock density for the reference year (i.e., 2006). To establish the historic changes of ruminant density from 1860 to 2012, two assumptions were made: 1) the distribution of ruminant density did not change during the time-span of this study (1860 - 2012); and 2) the changes in the ruminant

density of each category in all grid cells of a country co-varied with the changes in category-specific ME requirement in that country. Thus the total ruminant density for grid-cell k in country j in year m ($D_{m,j,k}$, with the unit of LU per ha of land area) is calculated as:

$$D_{m,j,k} = \sum(D_{ref,i,j,k} \times \frac{ME_{m,i,j}}{ME_{ref,i,j}}) \qquad (5)$$

where $D_{ref,i,j,k}$ is the ruminant density of category i for grid-cell k in country j in the reference year (i.e., 2006); $ME_{m,i,j}$ and $ME_{ref,i,j}$ (units of both: MJ yr^{-1}) are the total ME requirement by ruminant category i for country j in year m and in the reference year, 2006, respectively . The method used to calculate the ME requirement is given in Supporting Information Text S1 of ref[7]. Here, the range of year m is from 1961 to 2012, since FAOSTAT [62] provides annual country-averaged statistical data for dairy cows, beef cattle, sheep and goats of livestock numbers (unit: head), and meat (carcass weight) or milk yield for the period from 1961 up to the present day.

For the period 1961-2012, the annual country-averaged statistical data for dairy cows, beef cattle, sheep and goats of livestock numbers (unit: head), and meat (carcass weight) or milk yield are available in FAOSTAT[62]. For the period 1890-1960, regional livestock numbers for 10-year intervals derived from ref[148-150] were scaled to match the 1961 FAOSTAT data (data processed by Dr. Kees Klein Goldewijk, and given for 17 world regions with the numbers of cattle, sheep and goats; available in the HYDE database: http://themasites.pbl.nl/tridion/en/themasites/hyde/landusedata/livestock/index-2.html). The 17 world regions were designated for global change research, as defined by ref[151]. Linear interpolation is applied to calculate the regional livestock numbers for each year. For the period 1860-1890, regional livestock numbers are assumed to experience the same rate of change as regional population during the period 1860–1890. Assuming the meat (carcass weight) and milk yields for the period of 1860-1960 are the same as those for 1961 from FAOSTAT [62], total ruminant stocking density for grid-cell k in region q in year m ($D_{m,p,k}$) is then simply extended to 1860-1960 through:

$$D_{m,q,k} = \sum(D_{ref,i,q,k} \times \frac{ME_{m,i,q}}{ME_{ref,i,q}}) \qquad (6)$$

where $D_{ref,i,q,k}$ is the ruminant density of category i for grid-cell k in region q in the reference

year (i.e., 2006); $ME_{m,i,q}$ and $ME_{ref,i,q}$ are the total ME requirement by ruminant category i for region q in year m and in the reference year, 2006, respectively. Regional livestock numbers were further hindcasted to 1750 using the same rate of change as that of the regional population during the period 1750-1860. These values were used to calculate CH_4 and N_2O emissions from domestic ruminants.

3. Manure and mineral fertilizer application over grassland

The nitrogen application rate of manure and mineral fertilizer over European grasslands (EU27) came from ref[152-154] (as used in ref[7]). For countries/regions other than the EU-27, the following data were used. The amount of manure-N fertilizer (excluding manure deposited during grazing) for 17 world regions in 1995 was derived from various sources (e.g., ref[155-157]): these are synthesized by ref [158,159]. For mineral-N fertilizers on grassland, country-scale data of fertilized area and mean fertilization rate for 1999/2000 are available in ref[160] with grassland/pasture being fertilized in 13 non-EU countries. The regional/country-scale data were downscaled to a 0.5° × 0.5° grid, and extended to cover the period 1860-2012.

To downscale the total amount of regional N fertilizer to grid-level (except for OECD Europe and Eastern Europe where gridded data are available), the rules suggested by ref[158] were used, namely: animal manure application to grasslands is assumed to occur in mixed farming systems that are defined as grasslands occurring in grid-cells where the arable land coverage exceeds 35% in developed countries and 15% in developing countries. Here, the grasslands that satisfy the above rules were cited as manure-suitable grassland ($A_{manure\text{-}suit,ref,q,k}$). In addition, assuming a higher ruminant density produces more manure, we calculate the manure-N application rate for grid-cell k in region q in the manure data reference year, 1995, ($N_{manure,ref,q,k}$) as:

$$N_{manure,ref,q,k} = \frac{Q_{manure,q}}{\sum(D_{ref,q,k} \times A_{manure-suit,ref,q,k})} \times D_{ref,q,k} \tag{7}$$

where $Q_{manure,q}$ is the total amount of manure-N fertilizer in region q from ref[158,159]; $A_{manure\text{-}suit,ref,q,k}$ is the manure-suitable grassland area for grid-cell k in region q; and $D_{ref,q,k}$ is the total domestic ruminant stocking density (including cattle, sheep and goats from the maps established in Supplementary Methods 1, Section *"Reconstructing the history of grassland management intensity"*) for grid-cell k in region q where manure-suitable grassland exists.

Given the assumption that a higher ruminant density produces more manure, the manure fertilizer application rate (N_{manure}) is assumed to change along with changes in the total ruminant stocking density (as calculated in Supplementary Methods 1, Section *"Reconstructing the history of grassland management intensity"*). It is calculated as:

$$N_{manure,m,k} = N_{manure,ref,k} \times \frac{D_{m,k}}{D_{ref,k}} \tag{8}$$

where $N_{manure,m,k}$ and $N_{manure,ref,k}$ is the manure N application rate for grid-cell k in year m and in the manure application data reference year, 1995, respectively; $D_{m,k}$ and $D_{ref,k}$ are the total domestic ruminant stocking density (from the maps established in Supplementary Methods 1, Section *"Reconstructing the history of grassland management intensity"*) in grid-cell k in year m and manure application data reference year 1995 respectively.

For mineral-N fertilizers on grassland, country-scale data of fertilized area and mean fertilization rate ($N_{mineral}$) for 1999/2000 are available in ref [160] with grassland/pasture being fertilized in 34 countries. Of these 34 countries, 21 of them are in the EU-27 for which gridded fertilizer application rate data is available. For the other 13 non-EU-27 countries, the fertilized areas are given, indicating that not all the grassland is fertilized. Thus the national mean application rates are applied on grid-cells with a total ruminant stocking density above a certain threshold. The value of this threshold is determined for each country, making the total grassland area of fertilized grids identical to the national fertilized grassland area reported by ref[160]. However, note that the regional total amount of mineral-N fertilizer aggregated from country-scale data in ref[160] is much lower than the values given in ref[159].

The temporal evolution of gridded mineral-N fertilization for the EU-27 has been described by ref[7]. For the other 13 countries, the country-scale total nitrogenous fertilizer consumption data ($Q_{mineral}$); derived from FAOSTAT[62]) were used to extrapolate the mineral-N application rate ($N_{mineral}$). For Azerbaijan and Belarus, where FAOSTAT only provide data for 1992-2002, the variation of nitrogenous fertilizer consumption by the former USSR is used for the period 1961-1991. The rate of mineral-N application for grid-cell k in year m in country j ($N_{mineral,m,j,k}$) changes along with the variation of country-scale $Q_{mineral}$, and is calculated as:

$$N_{mineral,m,j,k} = N_{mineral,ref,j,k} \times \frac{Q_{m,j}}{Q_{ref,j}} \tag{9}$$

where $N_{mineral,ref,k}$ is the mineral-N application rate for grid-cell k in the mineral application data reference year, 2000, which is given by ref[160]; $Q_{m,j}$ and $Q_{2000,j}$ are the country-scale total nitrogenous fertilizer consumption in country j in year m and in the mineral application data reference year, 2000, respectively. The mineral-N fertilization rate after 2002 is taken as a constant using the value for 2002. For the period 1901-1960, the same set of rules as applied for the EU-27 (see section 'Simulation set-up' in ref[7] for details) are used: 1) no mineral-N fertilizer was applied over grassland before 1950; and 2) for the period 1951-1961, the rate of application is assumed to increase linearly from zero to the level of 1961.

4. Managed grassland area with the fraction that is mown or grazed

We combined model output, grass biomass use data and grassland area data to reconstruct managed grassland area with the fraction that is mown or grazed. Ref[63] established a global livestock production data set containing a high-resolution (8 km × 8 km) gridded map of grass-biomass use for the year 2000. Here, this data set is extrapolated annually over the period 1860-2012 to constrain the grass-biomass consumption in ORCHIDEE-GM v3.2. Assuming that grass-biomass use for grid cell k in country j and year m ($GBU_{m,j,k}$, unit: kg dry matter (DM) per year) varies proportionally with the total ME requirement of domestic ruminants in each country, $GBU_{m,j,k}$ can be calculated from its value in the year 2000 given by Ref[63], according to :

$$GBU_{m,k} = GBU_{2000,k} \times \frac{D_{m,k}}{D_{2000,k}} \quad (10)$$

where $D_{m.k}$ and $D_{2000,k}$ are the total ruminant stocking density for grid-cell k in year m and in year 2000 calculated in Supplementary Methods 1, Section *"Reconstructing the history of grassland management intensity"*, which take into account the changes in category-specific ME requirement at country-scale (1961-2012) or regional-scale (1860-1960).

ORCHIDEE-GM simulates the annual potential (maximal) harvested biomass from mown grasslands (Y_{mown}, unit: kg DM m^{-2} yr^{-1} from mown grassland) and the annual potential biomass consumption per unit area of grazed grassland (Y_{grazed}, unit: kg DM m^{-2} yr^{-1} from grazed grassland) in each grid-cell. Under mowing, the frequency and magnitude of forage harvests in each grid cell is a function of grown biomass [130]. The effective yield on grazed grassland (i.e., Y_{grazed}) depends on the grazing stocking rate (here, $D_{grazing}$) and on the environmental conditions of the grid cell [7], and calculated as:

$$Y_{grazed,m,k} = IC \times T_{grazing,m,k} \times D_{grazing,m,k} \tag{11}$$

where IC is the daily intake capacity for 1 LU (~ 18 kg dry matter per day calculated in Supporting information Text S1 of ref[7]), $T_{grazing,m,k}$ is the number of grazing days in grid cell k at year m. Due to the impact of livestock on grass growth through trampling, defoliation (i.e., biomass intake) etc., thresholds of shoot biomass, depending on the grazing stocking rate, are set for starting, stopping and resuming grazing [87]. For each grid cell, we set the biomass threshold for starting grazing as B_{start}, and the threshold for stopping grazing as $0.45{\times}B_{start}$, and made the grass biomass between the two thresholds enough to be continually grazed for 60 days under the grazing stocking density in that grid cell. The thresholds then determine when grazing stops ($0.45{\times}B_{start}$), or when grazing can start again (dry biomass recovered to the thresholds B_{start} for at least 15 days). Y_{grazed} is usually lower than Y_{mown} in temperate grasslands, due to the lower herbage-use efficiency of grazing simulated by ORCHIDEE-GM[87]. However, in some arid regions, the grass biomass does not grow enough during the season to trigger harvest, i.e., it does not reach the threshold in the model at which farmers are assumed to decide to cut grass for feeding forage to animals (see ref[87]), so that Y_{grazed} can become larger than Y_{mown}. The following set of rules was used to reconstruct historical changes in grassland management intensity, based on Y_{grazed} and Y_{mown} simulated by ORCHIDEE-GM:

Rule-1: for each grid-cell and year, the total biomass removed by either grazing and cutting must be equal to the grass-biomass use, $GBU_{m,k}$;

Rule-2: grazing management has priority in fulfilling $GBU_{m,k}$;

Rule-3: if the potential biomass consumption from grazing (Y_{grazed}) is not high enough to fulfil $GBU_{m,j,k}$, a combination of grazing and mowing management is taken.

Thus, for grid-cell k in year m, the *minimum* fraction of grazed ($f_{grazed,m,k}$), the *minimum* fraction of mown ($f_{mown,m,k}$) and the *maximum* fraction of unmanaged grassland ($f_{unmanaged,m,k}$) are calculated with the following equations (definitions of *minimum* and *maximum* in this context are given below).

If $A_{grass,m,k} \times Y_{grazed,m,k} > GBU_{m,k}$, then:

$$f_{grazed,m,k} = \frac{GBU_{m,k}}{A_{grass,m,k} \times Y_{grazed,m,k}} \tag{12}$$

$$f_{mown,m,k} = 0 \tag{13}$$

$$f_{unmanaged,m,k} = 1 - f_{grazed,m,k} \tag{14}$$

where $A_{grass,m,k}$ (unit: m^2) is the grassland area for grid-cell k in year m of the series of historic land-cover change maps.

If $A_{grass,m,k} \times Y_{grazed,m,k} < GBU_{m,k}$, and $A_{grass,m,k} \times Y_{mown,m,k} > GBU_{m,k}$, then:

$$f_{grazed,m,k} \times A_{grass,m,k} \times Y_{grazed,m,k} + f_{mown,m,k} \times A_{grass,m,k} \times Y_{mown,m,k} = GBU_{m,k} \tag{15}$$

$$f_{grazed,m,k} + f_{mown,m,k} = 1 \tag{16}$$

$$f_{unmanaged,m,k} = 0 \tag{17}$$

If $GBU_{m,k}$ cannot be fulfilled by any combination of modelled Y_{grazed} and Y_{mown}, we diagnose a *modelled grass-biomass production deficit* and apply the following equations :

if $Y_{grazed} > Y_{mown}$, then $f_{grazed,m,k} = 1$, $f_{mown,m,k} = 0$, and $f_{unmanaged,m,k} = 0$ (18)

if $Y_{grazed} < Y_{mown}$, then $f_{mown,m,k} = 1$, $f_{grazed,m,k} = 0$, and $f_{unmanaged,m,k} = 0$ (19)

This set of equations is valid for a mosaic of different types of grasslands in each grid-cell, some managed (grazed and/or mown) and some remaining unmanaged. In reality, 1) farm owners could increase the mown fraction to produce more forage. A situation which corresponds approximately to the *mixed and landless* systems of ref[161]; and 2) animals could migrate a long way across grazed and unmanaged fractions (as they do in real rangelands) and only select the most digestible grass in pastoral systems, which corresponds to *extensively grazed* grasslands. Yet, given the approximations made in this study, $f_{grazed,m,k}$ and $f_{mown,m,k}$ represent the *minimum* fractions of grazed/mown grasslands rather than the actual fractions, and on the other hand $f_{unmanaged,m,k}$ corresponds to a *maximum* fraction of unmanaged grasslands since both *mixed and land less* and *extensive grazing* are not modelled.

Reconstructing the history of wild grazer density and grazed area

Human activities not only impacted grassland through changing management intensity by introducing domestic livestock, but also caused large reductions in wild grazer populations by land-use change, animal kill-off and spreading diseases. The result is the relatively small population of wild grazers left today. The reduction of wild grazers may significantly influence the historical grassland GHG fluxes (e.g., CH_4[64]). Thus, the GHG fluxes from wild grazers should be accounted for in the GHG balance of grassland. To our knowledge, however, detailed historical wild grazer distribution and density maps do not exist. We therefore combined wild animal species information[64], diet information[65], modelled natural grassland productivity and an anthropogenic biome classification system[67] to reconstruct the gridded history of wild grazer density, and the wild and semi-natural grasslands occupied by wild grazers.

Pre-industrial wild mammal (grazers, browsers and mixed feeders of each continent for the year 1800) data were derived from Table S1 of ref[64], including body mass, animal density, geographic range and CH_4 emission. Five continents were differentiated by ref[64], namely: Africa, Australia, Eurasia, North America and South America. Focusing on grassland, we extracted data on mammals that consume mainly grass biomass (i.e., mainly grazers) based on diet of genera from Appendix S1 of ref[65] (continent-specific diet for each genus). For mixed feeders, we assumed half of their diet coming from grass biomass. For genera without diet information given by ref[65] (mainly small mammals), we searched the Internet (e.g., Wikipedia) for the diet composition. Given that only an “average grazer” can be assumed in ORCHIDEE-GM v3.2, a variety of wild grazers were aggregated and unified to livestock unit (LU as for domestic livestock) based on their dry matter intake (DMI). DMI for wild mammal genus *i* in continent *j* ($DMI_{wild,i,j}$; unit: kg dry matter day^{-1}) was calculated following IPCC algorithms[145] (IPCC, 2006 Vol 4, Chapter 10, Eqn 10.18a):

$$DMI_{wild,i,j} = BM_{wild,i,j}^{0.75} \times \left(\frac{0.0119 \times NE_{ma}^2 + 0.1938}{NE_{ma}}\right) \quad (20)$$

where $BM_{wild,i,j}$ is the body mass of wild mammal genus *i* in continent *j*; NE_{ma} is the dietary net energy concentration, with an intermediate value of 6.0 MJ kg^{-1} dry matter, corresponding to moderate quality forage as used in ref [133]. Total wild grazer numbers ($N_{wild1800,j}$; unit: LU) for continent j in 1800 were calculated as:

$$N_{wild1800,j} = \sum_i \left(DMI_{wild,i,j} \times D_{wild,i,j} \times A_{wild,i,j}\right) / DMI_{LU} \quad (21)$$

where $D_{wild,i,j}$ and $A_{wild,i,j}$ are animal density (unit: number km^{-2}; originated from the equations

of ref[162]) and geographic range (unit: km^2; originated from ref[163], respectively, as defined by ref[64]; DMI_{LU} is the dry matter intake of one standard livestock unit, i.e., 18 kg dry matter per day as calculated in Supporting Information Text S1 of ref[7].

To spatialize the continental wild grazer numbers of 1800 on to a gridded map, we applied a similar strategy to that in the Supporting Information Text S4 of ref [133]. Assuming higher consumable NPP supports more wild grazers, the wild grazer density in 1800 for grid-cell k in continent j ($D_{wild1800,j,k}$; unit: LU km^{-2}) is calculated as:

$$D_{wild1800,j,k} = \frac{N_{wild,j}}{\sum_k (ANPP_{unmanaged,j,k} \times A_{wild1800,j,k})} \times ANPP_{unmanaged,j,k} \quad (22)$$

where $ANPP_{unmanaged,j,k}$ is the aboveground NPP of sparsely grazed grassland for grid-cell k simulated by ORCHIDEE-GM v3.2 for the 1800s (in this study forced by 1901–1910 climate and pre-industrial CO_2 concentration; simulation E2 in Supplementary Table 1); $A_{wild1800,j,k}$ is the grassland area occupied by wild grazers in 1800 for grid-cell k. To estimate the reduction of wild large grazers due to human land use, we followed the assumption of ref[164] and made use of the anthropogenic biome classification system from the Anthromes products[67], which separated three major categories: used, semi-natural, and wild. We assumed the remnant habitat for wild grazers to be the "wild and semi-natural" categories. Thus $A_{wild1800,j,k}$ is calculated as:

$$A_{wild1800,j,k} = A_{unmanaged1860,j,k} \times f_{w+s1800,j,k} \quad (23)$$

where $A_{unmanaged1860,j,k}$ is the area of sparsely grazed grassland in 1860 for grid-cell k from the reconstructed historical maps on grassland management intensity (see Supplementary Methods 2 *"Model input"* and Supplementary Figure 1), assuming there is no change in the area of sparsely grazed grassland between 1800 and 1860; $f_{w+s1800,j,k}$ is the remnant habitat fraction in 1800 assumed for wild grazers at the spatial resolution of 0.5° × 0.5°, which is aggregated from the 5'× 5' resolution of the Anthromes products. Decadal Anthromes products were obtained from the HYDE3.2.1 data set[141] (available at: ftp://ftp.pbl.nl/hyde/hyde3.2/anthromes/). Constant wild grazer density ($D_{wild1800,j,k}$) is assumed from 1800 to 2012, except for two major events where major reductions in wild grazers were caused by factors other than human land use. They are the North America Great Plains bison kill-off during the 1860s and 1870s, and the African rinderpest epizootic during the 1890s. After these two events (i.e., in 1880 for North America, and in 1900 for Africa), wild grazer density ($D_{wild,j,k}$) is assumed to decrease by the same fraction as the reduction of aggregated continental total wild grazer numbers:

$$D_{wild,event,j,k} = D_{wild1800,j,k} \times \frac{N_{wild,event,j}}{N_{wild1800,j}} \qquad (24)$$

where $D_{wild,event,j,k}$ is the wild grazer density at the end of each event for grid-cell k in the corresponding continent j; and $N_{wild,event,j}$ is the residual total wild grazer numbers at the end of each event for the corresponding continent j, which can be calculated from Supplementary Equations 21 and 22 using wild mammal reduction data from ref[64]. During the events (i.e., 1860-1880 for North America, and 1890–1900 for Africa), wild grazer density ($D_{wild,j,k}$) decreased linearly. Following the changes in the sparsely grazed grassland from the reconstructed historical maps on grassland management intensity ($A_{unmanaged}$), and the remnant habitat fraction for wild grazers (f_{w+s}) derived from the HYDE3.2.1 data set, we estimated an 83% decrease of global total wild grazer numbers since 1800, with regional differences (Supplementary Figure 2), similar to the extent estimated by ref[164]. To reflect more realistically the changes in wild grazers, two issues relating to this study should be noted. First, no wild grazers were distributed over high latitude Siberian grassland (mainly tundra), considering that few herbivore species exists there[165] (mainly moose and reindeer as mixed feeders). Second, wild grazer density changes in Australia following human population introduction starting around 1875 ref[64] were neglected, because population growth and the expansion of its geographic range was slow.

Simulation set-up

In the simulation of the grassland GHG balance, we assume that global grasslands were managed for a long time before 1750, and that the 1860 land cover and grassland management intensity applied equally before 1860. The series of simulations are shown in Supplementary Table 1. ORCHIDEE-GM v3.2 is first run for a spin-up period (simulation E1) by recycling the first 10 years of climate forcing (1901–1910) in a loop with land-cover, grassland management, wild grazer density, and CO_2 concentration fixed at the level for 1860 (286 ppm for CO_2) until an equilibrium is reached for all the carbon pools at each grid point (long-term net ecosystem exchange, NEE = 0 at each grid point). This spin-up usually takes 20,000 years. Starting from soil carbon pools in equilibrium for year 1860 (end of the spin-up), a second simulation (simulation E2) is then conducted for the period 1751–1860 with grassland management, wild-grazer grazing, manure application (no mineral fertilizer applied at that time), and atmospheric-nitrogen deposition of 1860 to reproduce the management history. For the period 1861–1900,

simulation E3 is carried out with the first 10 years of climate forcing (1901–1910) cycled, and other inputs variable. As a final simulation (simulation E4), ORCHIDEE-GM v3.2 is run for the period 1901–2012 forced by all input variables (Supplementary Table 1).

The calculation of the components of the grassland GHG balance

The grassland GHG balance at ecosystem and farm scale in this study is calculated using equation (1) of the Methods section. Each component of the grassland GHG balance is estimated as follows.

1. The CO_2 fluxes of grassland ecosystem and farm scale and land-use change emissions due to deforestation for pasture and conversion of grassland to cropland

We define the grassland net CO_2 fluxes ($F_{CO_2\text{-}C}$) as the net carbon balance of a grassland ecosystem assuming that there is no long-term storage of the harvested carbon. It is calculated as:

$$F_{CO_2-C} = NEE + F_{CH_4-C-eco} + F_{harvest} + F_{erosion} - F_{input} \tag{25}$$

where $F_{CH_4\text{-}C\text{-}eco}$ is the enteric fermentation CH_4 emission from grazing livestock; $F_{harvest}$ is the carbon exported as harvested biomass (grass forage); $F_{erosion}$ is the net carbon fluxes induced by water erosion including erosion removal fluxes, and the compensatory soil sink (see Supplementary Discussion 5); and F_{input} is the carbon input into the system through organic fertilizers; and *NEE* is net ecosystem exchange of grassland ecosystem calculated as:

$$NEE = R_h + R_a + R_{animal-eco} + F_{fire} - GPP \tag{26}$$

where R_h is the heterotrophic respiration from soil; R_a is the plant autotrophic respiration; $R_{animal\text{-}eco}$ is the respiration of grazing livestock; F_{fire} is the fire emissions (see Supplementary Discussion 6); and *GPP* is the plant gross primary productivity through photosynthesis. All the above fluxes are either simulated by the model (e.g., NEE and its components, $F_{CH_4\text{-}C\text{-}eco}$, $F_{harvest}$) or included as input data (F_{input}). Negative fluxes indicate a carbon sink, and positive fluxes indicate a carbon source.

To fully account for the grassland carbon balance, carbon losses due to deforestation to pasture and conversion of grassland to cropland (E_{LUC}) are additionally identified. The method of estimating E_{LUC} is described in the Methods section.

2. Grassland ecosystem and off-field CH_4 fluxes

The grassland CH_4 emission ($F_{CH_4\text{-}C}$) can be expressed as the combination of the CH_4 flux from the ecosystem ($F_{CH_4\text{-}C\text{-}eco}$) and that from the off-field farm system ($F_{CH_4\text{-}C\text{-}farm}$):

$$F_{CH_4-C} = F_{CH_4-C-eco} + F_{CH_4-C-farm} \tag{27}$$

where $F_{CH_4\text{-}C\text{-}eco}$ is the enteric fermentation CH_4 emission from grazing livestock that can be simulated by ORCHIDEE-GM[126,130] (with uncertainty of ±30 to ±50% from ref[145] IPCC, 2006 Vol 4, Chapter 10, pp10.33). The off-field CH_4 emissions (i.e., at farm scale; $F_{CH_4\text{-}C\text{-}farm}$) come from enteric fermentation of housed livestock ($F_{CH_4\text{-}C\text{-}EFfarm}$) and from manure management ($F_{CH_4\text{-}C\text{-}MM}$):

$$F_{CH_4-C-farm} = F_{CH_4-C-EFfarm} + F_{CH_4-C-MM} \tag{28}$$

Assuming the enteric fermentation CH_4 emission rate ($R_{CH_4\text{-}C\text{-}EF}$; unit: g CH_4-C per kg dry matter (DM) intake) is the same for grazing livestock as for housed livestock eating grass forage inside. $F_{CH_4\text{-}C\text{-}EFfarm}$ is calculated as:

$$F_{CH_4-C-EFfarm} = Y_{mown} \times R_{CH_4-C-EF} \tag{29}$$

where Y_{mown} (unit: kg DM) is the total amount of grass dry matter harvested from mown grassland of each grid cell; and $R_{CH_4\text{-}C\text{-}EF}$ is calculated as:

$$R_{CH_4-C-EF} = \frac{F_{CH_4-C-eco}}{Y_{grazed}} \tag{30}$$

where $F_{CH_4\text{-}C\text{-}eco}$ is the enteric fermentation CH_4 emission from grazing livestock, and Y_{grazed} (unit: kg DM) is the total amount of dry matter intake by grazing for each grid cell.

The CH_4 emission from manure management ($F_{CH4\text{-}MM}$) in each grid cell can be calculated from the livestock numbers fed by harvested biomass from grassland following the IPCC Tier 1

algorithms[145] (IPCC, 2006 Vol 4, Chapter 10, Eqn 10.22), but note that it depends on livestock types as well as on temperature:

$$F_{CH_4-C-MM} = \sum N_i \times EF_{5(i)} \tag{31}$$

where $EF_{5(i)}$ is the emission factor for CH_4 emitted from manure management (unit: kg CH_4 head^{-1} yr^{-1}) depending on livestock categories, regions, as well as temperature[145] (IPCC, 2006 Vol 4, Chapter 10, Table 10.14); for each grid cell, mean annual temperature derived from climate forcing is used to determine the $EF_{5(i)}$. The uncertainty of $EF_{5(i)}$ is ±30%[145] (IPCC, 2006 Vol 4, Chapter 10, pp10.39; uncertainty expressed as half the 95 per cent confidence interval divided by the mean and expressed as a percentage). N_i is livestock number (unit: head) of category i (i.e., beef cattle, dairy cows, sheep, and goats) that can be fed by Y_{mown} in each grid cell. N_i is calculated as:

$$N_i = N_{LU} \times f_{i,j} \times \frac{ME_{LU}}{ME_{head,i,j}} \tag{32}$$

where N_{LU} is the total livestock number (unit: LU) that can be fed by Y_{mown} in each grid cell; $f_{i,j}$ is the fraction of livestock category i in country j, and is calculated based on the total metabolisable energy (ME) requirement of livestock category i and of all livestock categories in country j; ME_{LU} (unit: MJ yr^{-1} LU^{-1}) is the ME requirement by one LU; and $ME_{head,i,j}$ (unit: MJ yr^{-1} head^{-1}) is the ME requirement per head of livestock category i in country j. The typical weight of each livestock category is given in ref[145] IPCC, 2006 Vol 4, Chapter 10, Table 10A-4, 10A-5, and 10A-9, and one LU is defined as an average adult dairy cow producing 3000 kg of milk annually, with live body weight of 600 kg[146]. The calculation of ME is described in the Supporting Information Text S1 of ref[7]. N_{LU} is calculated as:

$$N_{LU} = \frac{Y_{mown}}{DMI_{LU} \times 365} \tag{33}$$

Y_{mown} (unit: kg DM) is the total amount of grass dry matter harvested from mown grassland of each grid cell; and DMI_{LU} = 18 kg DM day^{-1} is daily dry matter intake by one LU, calculated by the ME requirement of ca. 85 MJ day^{-1} per LU divided by the energy density of the feed (with a default value of 18.45 MJ kg^{-1} of dry matter[145]).

CH_4 emission from wild grazers largely depends on animal body mass [133]. Thus to account for the body mass effect, we directly use the wild mammals' CH_4 emissions from ref[64]. Similar to

the continental total wild grazer numbers ($N_{wild1800,j}$) extracted in the Supplementary Methods 1 Section *"Reconstructing the history of wild grazer density and grazed area"*, we extracted and aggregated continental total CH_4 emissions from wild grazers ($F_{CH_4,wild1800,j}$) from ref[64], and calculated the CH_4 emission factor for wild grazers in continent j ($EF_{CH4,wild1800,j}$; unit: kg CH_4 LU^{-1} $year^{-1}$) as:

$$EF_{CH_4,wild} = \frac{F_{CH_4,wild1800,j}}{N_{wild1800,j}} \quad (34)$$

As for wild grazer density ($D_{wild1800,j,k}$), a constant $EF_{CH4,wild1800,j}$ is assumed from 1800 to 2012. Combining the wild animal CH_4 emissions[64] and diet information[65], we estimated wild grazer CH_4 emission of 11.9 Tg CH_4 yr^{-1} for the year 1800, which comprises 46% of total wild mammals' CH_4 emissions from ref[64].

3. Grassland ecosystem and off-field N_2O fluxes

The grassland N_2O emission ($F_{N_2O\text{-}N}$) can be expressed as the combination of N_2O fluxes from managed soil in the ecosystem ($F_{N_2O\text{-}N\text{-}eco}$) and those from manure management at the farm level ($F_{N_2O\text{-}N\text{-}farm}$):

$$F_{N_2O-N} = F_{N_2O-N-eco} + F_{N_2O-N-farm} \quad (35)$$

The N_2O fluxes are calculated as follows.

N_2O emission from managed soil

The N_2O emissions from managed soil are mainly due to the application of nitrogen to grassland as manure or mineral fertilizer, by excreta from grazing livestock including wild grazers, and through atmospheric deposition in the form of oxidized nitrogen (NO_y) and reduced nitrogen (NH_x). It includes direct N_2O emissions from managed soil ($F_{directN2O\text{-}N\text{-}eco}$), indirect N_2O emissions from atmospheric deposition including nitrogen volatilized from managed soils and transported from other sources ($F_{volN2O\text{-}N\text{-}eco}$), and indirect N_2O emissions from nitrogen leaching/runoff from managed soils in regions where leaching/runoff occurs ($F_{leachN2O\text{-}N\text{-}eco}$):

$$F_{N_2O-N-eco} = F_{directN_2O-N-eco} + F_{volN_2O-N-eco} + F_{leachN_2O-N-eco} \quad (36)$$

All the N_2O emissions were calculated following the IPCC Tier 1 algorithms[145] (IPCC, 2006 Vol 4, Chapter 11, Eqns 11.1, 11.9 and 11.10). Direct N_2O emissions from managed soil are calculated as:

$$F_{directN_2O-N-eco} = (F_{SN} + F_{ON}) \times EF_1 + F_{PRP} \times EF_2 \quad (37)$$

Indirect N_2O emissions from atmospheric deposition (including nitrogen volatilized from managed soils and transported from other sources) are calculated as:

$$F_{volN_2O-N-eco} = F_{dep} \times EF_3 \quad (38)$$

Indirect N_2O emissions from nitrogen leaching/runoff from managed soils in regions where leaching/runoff occurs are calculated as:

$$F_{leachN_2O-N-eco} = (F_{SN} + F_{ON} + F_{PRP} + F_{dep}) \times Frac_{LEACH-H} \times EF_4 \quad (39)$$

where:

F_{SN} = annual amount of mineral fertilizer nitrogen applied to soils and atmospheric nitrogen deposition;

F_{ON} = annual amount of animal manure, compost, sewage sludge and other organic nitrogen additions applied to soils;

F_{PRP} = annual amount of nitrogen deposited as urine and dung by grazing animals on grassland, including wild grazers;

F_{dep} = annual amount of atmospheric nitrogen deposition over grasslands;

$Frac_{LEACH-H}$ = fraction of all nitrogen added to, or mineralized in, managed soils in regions where leaching/runoff occurs that is lost through leaching and runoff;

EF_1 = 0.01 (uncertainty range of 0.003 to 0.030); EF_2 = 0.02 (uncertainty range of 0.007 to 0.060) for cattle, and 0.01 (uncertainty range of 0.003 to 0.030) for sheep; EF_3 = 0.01 (uncertainty range of 0.002 to 0.050); EF_4 = 0.0075 (uncertainty range of 0.0005 to 0.0250); $Frac_{LEACH-H}$ = 0.30 (uncertainty range of 0.10 to 0.80). The default values of these parameters and emission factors and their uncertainty ranges are from guidelines[145] (IPCC, 2006 Vol 4, Chapter 11, Table 11.1 and 11.3); F_{SN} and F_{ON} come from the gridded nitrogen addition maps (including nitrogen fertilizer application maps with manure-nitrogen and mineral-nitrogen

fertilizers, and atmospheric-nitrogen deposition maps; see Supplementary Methods 1, Section *"Model input"*. and ref[133] for detail); F_{PRP} is calculated in ORCHIDEE-GM v3.2. It is noteworthy that ref[166] reports a lower emission factor (0.0075 instead of 0.01 in ref[145]) of direct N_2O emission from managed soil; F_{dep} during 1860–2012 were from the IGAC/SPARC Chemistry-Climate Model Initiative (CCMI) deposition fields[142].

In this study, we assume leaching would not occur over regions with an arid climate in the Koppen-Geiger climate classification[167].

Off-field N_2O emissions from manure management

Following the IPCC Tier 1 algorithms[145] (IPCC, 2006 Vol 4, Chapter 10), the N_2O can be directly emitted from manure management ($F_{directN2O\text{-}N\text{-}MM}$), indirectly emitted due to volatilization of nitrogen from manure management ($F_{volN2O\text{-}N\text{-}MM}$), and indirectly emitted due to leaching/runoff from manure management in regions where leaching/runoff occurs ($F_{leachN2O\text{-}N\text{-}MM}$):

$$F_{N_2O-N-MM} = F_{directN_2O-N-MM} + F_{volN_2O-N-MM} + F_{leachN_2O-N-MM} \quad (40)$$

All the N_2O emissions are calculated following the IPCC Tier 1 algorithms[145] (IPCC, 2006 Vol 4, Chapter 10, Eqn 10.25). Direct N_2O emission from manure management is calculated as:

$$F_{directN_2O-N-MM} = \sum(Nex_i \times EF_6 \times N_i \times MS_i) \quad (41)$$

where N_i is livestock number (unit: head) of category i (i.e., beef cattle, dairy cows, sheep, and goats) that can be fed by Y_{mown} in each grid cell as calculated in Equation (14); MS_i is the fraction of total annual nitrogen excretion that is managed in the manure management system; EF_6 is the emission factor for direct N_2O emissions from the manure management system (unit: kg N_2O-N per kg N in manure management system). The manure management system information is derived from ref[145] (IPCC, 2006 Vol 4, Chapter 10, Table 10A.4 and 10A.5). EF_6 is derived from ref[145] (IPCC, 2006 Vol 4, Chapter 10, Table 10.21) for each system. Nex_i is annual average nitrogen excretion (unit: kg N head^{-1} yr^{-1}), which is calculated as ref[145] (IPCC, 2006 Vol 4, Chapter 10, Eqn 10.30):

$$Nex_i = (N_{rate(i)} \times TAM_i / 1000) \times 365 \quad (42)$$

where $N_{rate(i)}$ is daily nitrogen excretion rate (unit: kg N per 1000 kg animal mass), and TAM_i is typical animal mass for livestock category (unit: kg per animal). The default value and the uncertainty of $N_{rate(i)}$ (50%) and TAM_i are adopted from ref[145] (IPCC, 2006 Vol 4, Chapter 10, Table 10.19, and Table 10A-4 to Table 10A-9).

Indirect N_2O emission due to volatilization from manure management is calculated as ref[145] (IPCC, 2006 Vol 4, Chapter 10, Eqn 10.26):

$$F_{volN_2O-N-MM} = \sum (Nex_i \times Frac_{gas(i)} \times N_i \times EF_7) \quad (43)$$

where $Frac_{gas(i)}$ is the percentage of managed manure nitrogen for each livestock category that volatilizes as NH_3 and NO_x in the manure management system (default value and uncertainty range for each type of livestock is derived from ref[145] (IPCC, 2006 Vol 4, Chapter 10, Table 10.22); EF_7 is emission factor for N_2O emissions from atmospheric deposition of nitrogen on soil and water surfaces given as the same default value of EF_3 with 0.01 kg N_2O-N per kg of NH_3-N+NO_x-N volatilized (uncertainty range of 0.002 to 0.050).

Indirect N_2O emission due to leaching from manure management systems is calculated as ref[145] (IPCC, 2006 Vol 4, Chapter 10, Eqn 10.28):

$$F_{leachN_2O-N-MM} = \sum (Nex_i \times Frac_{leach(i)} \times N_i \times EF_8) \quad (44)$$

where $Frac_{leach(i)}$ is the percent of managed manure nitrogen losses for livestock category due to runoff and leaching during solid and liquid storage of manure (typical range 1%–20%; 5% is used in this study as the default value); EF_8 is emission factor for N_2O emissions from nitrogen leaching and runoff given as the same default value of EF_4 with 0.0075 kg N_2O-N per kg of N leaching/runoff (uncertainty range of 0.0005 to 0.0250).

Supplementary Methods 2. Model and methods used for radiative forcing attribution

Radiative forcing due to the changes in grassland GHG fluxes

1. OSCAR v3.1

OSCAR is a compact Earth system model calibrated to emulate the behaviour of more complex models. All components of the Earth system necessary to simulate future climate change are represented in the model. Notably, the model includes an ocean carbon cycle, a land carbon cycle, and atmospheric chemistry for CH_4 and N_2O. Radiative forcings from CH_4 reported in

this study include the direct effect of atmospheric CH_4 concentration change, but also the effect of ensuing changes in stratospheric H_2O and tropospheric O_3 concentrations. Similarly, the reported radiative forcings from N_2O include the direct effect of atmospheric N_2O concentration change, and that of the ensuing change in stratospheric O_3.

Compared to v2.2 (ref[119]), OSCAR v3.1 is a major update of the model. All successive updates are described on the GitHub repository where OSCAR can be freely downloaded (https://github.com/tgasser/OSCAR).

2. OSCAR v3.1 simulation setup

The simulation setup is briefly described in the Methods section. The detailed description of the OSCAR v3.1 simulation setup is as follows.

As preliminary simulations for this study, in addition to climate driving data, OSCAR is also forced with the observed time series of atmospheric concentrations of CO_2[168], CH_4 and N_2O[169], and reconstructed time series before direct observations were possible[170]. The model is then run for the first time to estimate compatible anthropogenic emissions of CO_2, CH_4 and N_2O: prescribed atmospheric concentrations are balanced with the natural fluxes simulated by OSCAR to deduce the compatible amount of yearly anthropogenic fluxes through simple mass conservation (see e.g., ref[171]). A second simulation is then realized, driven this time by the compatible emissions deduced from the first simulation, to check that the simulated atmospheric concentrations of CO_2, CH_4 and N_2O are the same as the observed ones. This second simulation is the control experiment.

The attribution is made to 9 regions and 8 sectors. Sectors are defined as emissions of CO_2, CH_4 and N_2O from managed versus sparsely grazed grasslands, and of CO_2 from land-use change emissions of deforestation to pasture and of conversion of grassland to cropland. The attribution protocol requires that, in addition to a control experiment, 73 simulations are made, one for each combination of region and sector, plus one for the rest (i.e. all the remaining emitting sectors unrelated to grasslands). In each of these simulations, the same emissions as in the control experiment are prescribed, except a small fraction of 1% of the region's sectoral emissions are removed. The difference between the control simulation and each of those factorial simulations, once normalized by the sum of the 73 differences, provides the contribution of each region and sector. This approach to attribution is called the "normalized marginal" method, and it is described further by ref[172] and references therein.

The uncertainty in the simulations was assessed through a Monte Carlo approach (n = 10,000) in which biophysical parameters of the model were randomly drawn (see ref[119]) and associated to every single set of anthropogenic grassland GHG fluxes of the first Monte Carlo ensemble.

Because OSCAR naturally produces a range of uncertainty larger than we considered reasonable for the three greenhouse gases simulated in this study, we weighted our Monte Carlo ensemble using existing emission inventory data sets as constraints.

For each ensemble member, the cumulative compatible emissions simulated by OSCAR in the second (i.e. control) experiment, in the case of fossil-fuel CO_2, CH_4, and N_2O are taken, and then compared to reference values. For fossil-fuel CO_2, we take the CDIAC data set over 1901–2010 and apply an uncertainty of 5%, for CH_4 and N_2O, we take the EDGAR data set over 1970–2008 and apply a 10% uncertainty.

Each ensemble member is then given three weights (one per GHG), calculated as the probability of drawing the ensemble member's cumulative emission value from a normal distribution whose average and standard deviation are the reference value and its associated uncertainty, respectively. This approach was previously used by ref[1] section 2.7.3. The three weights are then multiplied together to obtain the final weights used to provide the weighted averages and standard deviations reported in the text and figures.

Radiative forcing from albedo change due to grassland-related land-cover change

The same method as used by OSCAR v3.1[173] is applied to assess the radiative forcing induced by changes in grassland-related land cover ($\mathrm{RF}_{\mathrm{grass}}^{\mathrm{LCC}}$). Here, grassland-related land-cover change is defined as both grassland converted from (grassland increase) and to (grassland decrease) other land-cover groups (i.e., biomes). Five biomes are differentiated in this study including bare soil, evergreen forest, deciduous forest, grassland and cropland. $\mathrm{RF}_{\mathrm{grass}}^{\mathrm{LCC}}$ is modelled following the first-order equation of ref[174], and is calculated as:

$$RF_{grass}^{LCC} = \frac{1}{2} \times (-\pi_{trans} \sum_{i,j} \varphi_{rsds}^{i,j} \sum_{b} \Delta\alpha_{alb}^{i,b,j} \frac{\Delta A^{i,b}}{A_{Earth}}) \qquad (45)$$

where the upward transmittance is set to $\pi_{\mathrm{trans}} = 0.854$ [175], A_{Earth} designates the surface area of the Earth (510.1×10^6 km^2), $\Delta\alpha_{alb}^{i,b,j}$ is the monthly averaged (month *j*) albedo difference between biome *b* and grassland at region *i*, $\Delta A^{i,b}$ is the area change between biome *b* and

grassland in region i, and $\varphi_{rsds}^{i,j}$ is the surface radiative shortwave-downward flux over the area with grassland-related land-cover change in region i. To ensure that the sum of biome-specific radiative forcing (i.e.,$\sum_b \mathrm{RF}_b^{\mathrm{LCC}}$) is equal to the radiative forcing induced by all land-cover changes (RF^{LCC} as in section 2.11 of ref[119]) and to avoid double counting the land-cover change, we divided the final $\mathrm{RF}_{\mathrm{grass}}^{\mathrm{LCC}}$ by two as in Equation (45).

The radiation fluxes φ_{rsds} are taken from one of three monthly climatologies: GEWEX[176] over the 1984–2007 period, CERES[177] over 2000–2014, or MERRA[178] over 1979–2014. The albedos α_{alb} are based on one of two monthly climatologies: either GlobAlbedo[179] over the 1998–2011 period, or MODIS (MCD43C3[180]) over 2001–2012. We calculate the monthly-averaged biome-specific albedos ($\alpha_{alb}^{i,b,j}$) by weighting the monthly albedo climatology by one of three land-cover maps – MODIS (MCD12Q1[181]), ESA-CCI[137] (https://www.esa-landcover-cci.org/) or GLC2000[182]– and by the monthly radiation climatology used for φ_{rsds}, in a similar fashion as ref[183]. This approach ensures that the monthly-averaged albedo accounts for the local seasonality, especially that of snow cover. The changes in grassland-related land cover ($\Delta A^{i,b}$) are derived from the historic land-cover change maps used in this study (see Supplementary Methods 1, Section *"Model input"*. and Supplementary Table 4 for detail), but is traced back to 1750 for simulating the $\mathrm{RF}_{\mathrm{grass}}^{\mathrm{LCC}}$.

Regarding the deduction of biome-specific albedos, we make the same assumptions as ref[119]. First, we weight the biome-aggregated albedos by their biome area fraction map, taken to the power 3. This approach is used to give more importance – in a given region – to the grid cells in which biomes are purer, without taking the risk of having too few of those grid cells if we were to set a threshold of biome area fraction instead. Second, we remove the grid cells that see less than 1% of their area changing over the historical period according to our land cover change maps (see Supplementary Methods 1, Section *"Model input"* and Supplementary Table 1 for detail).

The uncertainty of $\mathrm{RF}_{\mathrm{grass}}^{\mathrm{LCC}}$ is assessed as the standard deviation of the 18 $\mathrm{RF}_{\mathrm{grass}}^{\mathrm{LCC}}$ derived from the different data sets' combinations with three land-cover maps, three radiation fluxes climatologies, and two albedo climatologies (see above).

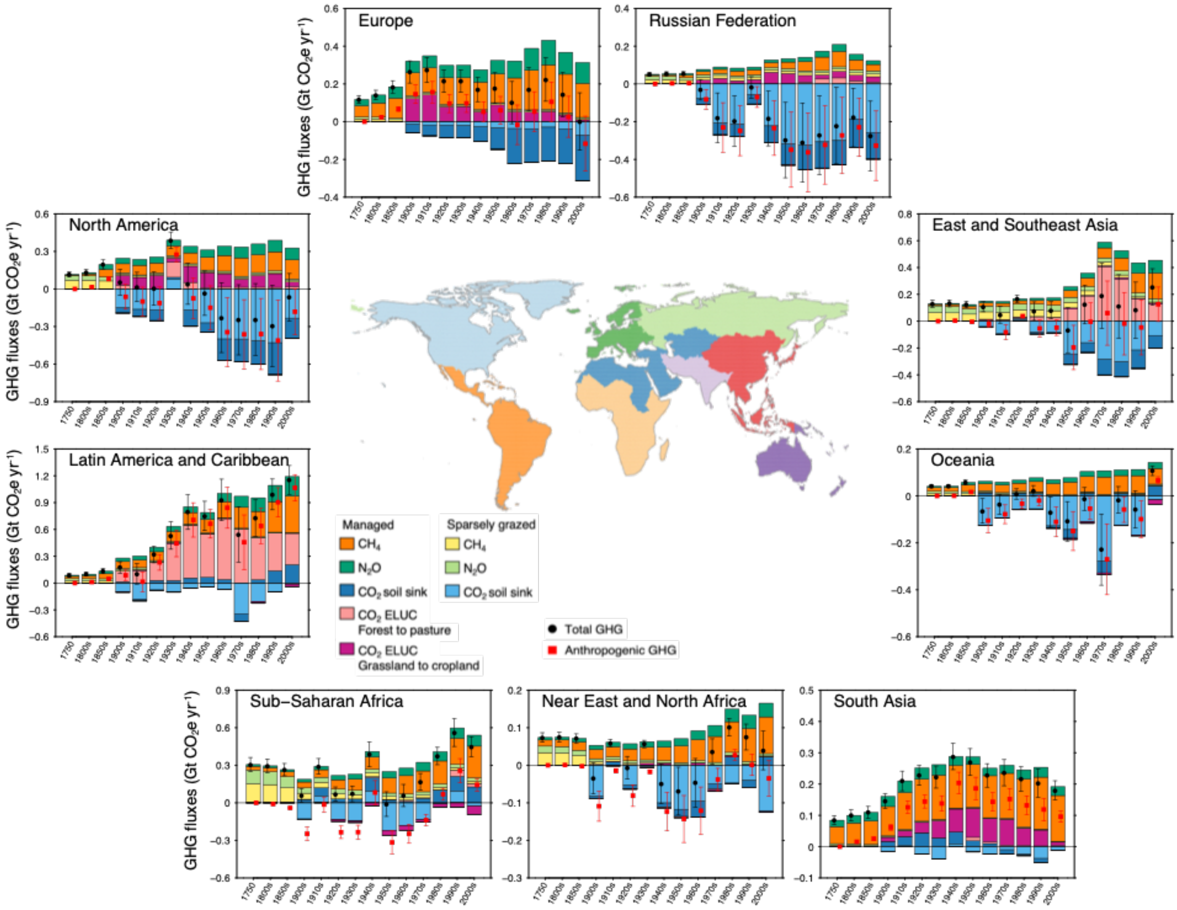

Supplementary Figure 1. The decadal greenhouse gas (GHG) fluxes for grassland in different regions since 1750. Light and dark blue bars represent CO_2 fluxes from managed and sparsely grazed grassland, respectively; orange and yellow represent CH_4 fluxes from managed (domestic livestock) and sparsely-grazed (wild grazers) grassland, respectively; light green and dark green represent N_2O fluxes from managed and sparsely grazed grassland, respectively; and pink and purple represents land-use change emissions related to grassland from deforestation to pasture and from conversion of grassland to cropland respectively. Black dots and their error bars indicate net total GHG balance and its 1-sigma uncertainty. Red squares and their error bars indicate the anthropogenic GHG balance after subtracting pre-industrial GHG fluxes. Negative values indicate GHG sinks and positive values indicate GHG sources. Regions are classified following the definitions in the FAO Global Livestock Environmental Assessment Model (GLEAM; http://www.fao.org/gleam/en/). We combined western and eastern Europe as "Europe" to avoid the individual regions being too small.

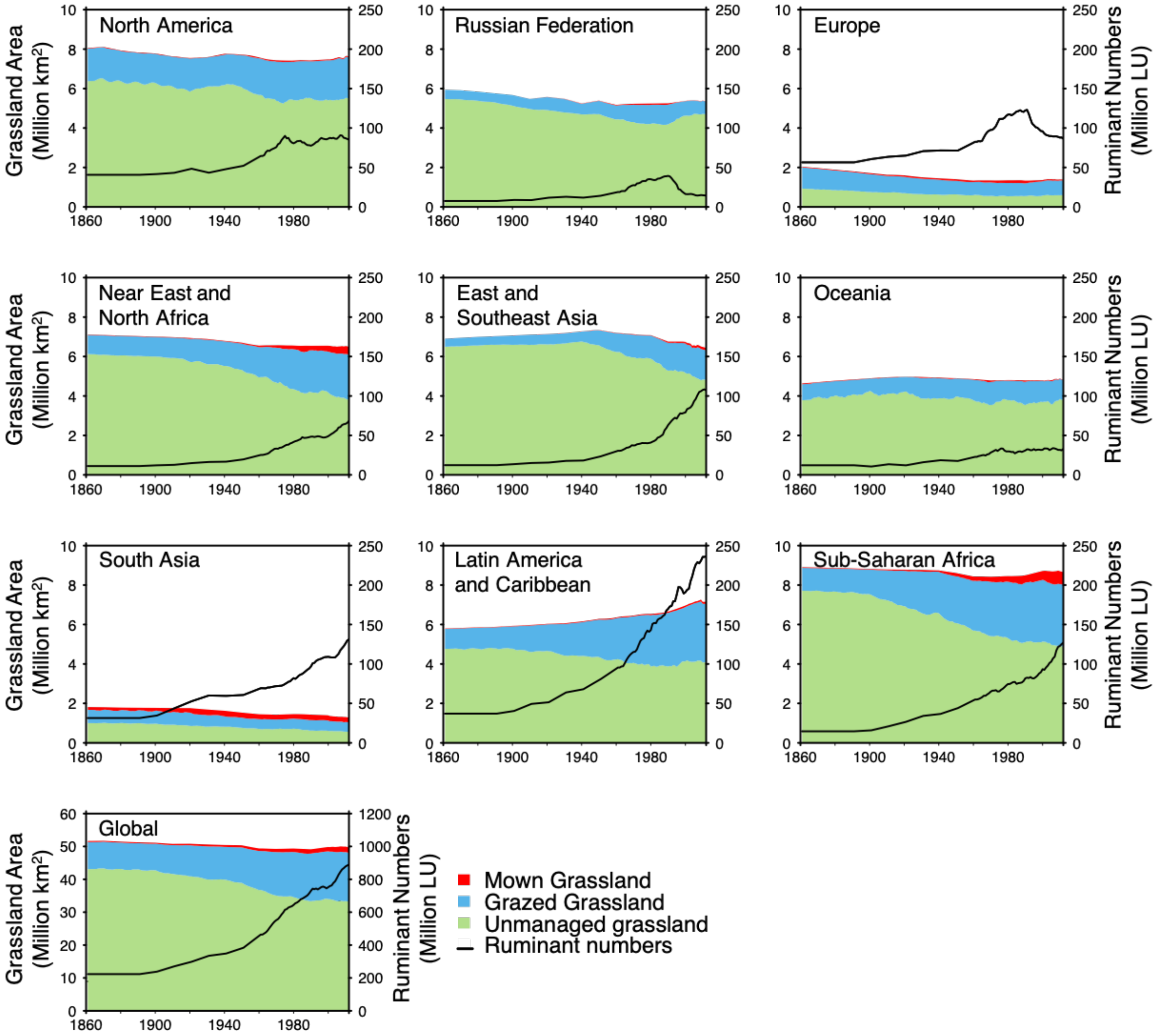

Supplementary Figure 2. Historical changes in the area of managed (mown and intensively grazed) and sparsely grazed grassland and in ruminant numbers for 1860 and 2012 by region, together with the global total. Data were reconstructed following the same procedures as in ref [133], but using different land-cover change maps (see Methods for detail). Regions are classified following the definitions in the FAO Global Livestock Environmental Assessment Model (GLEAM; http://www.fao.org/gleam/en/). We combined western and eastern Europe as "Europe" to avoid any individual region being too small.

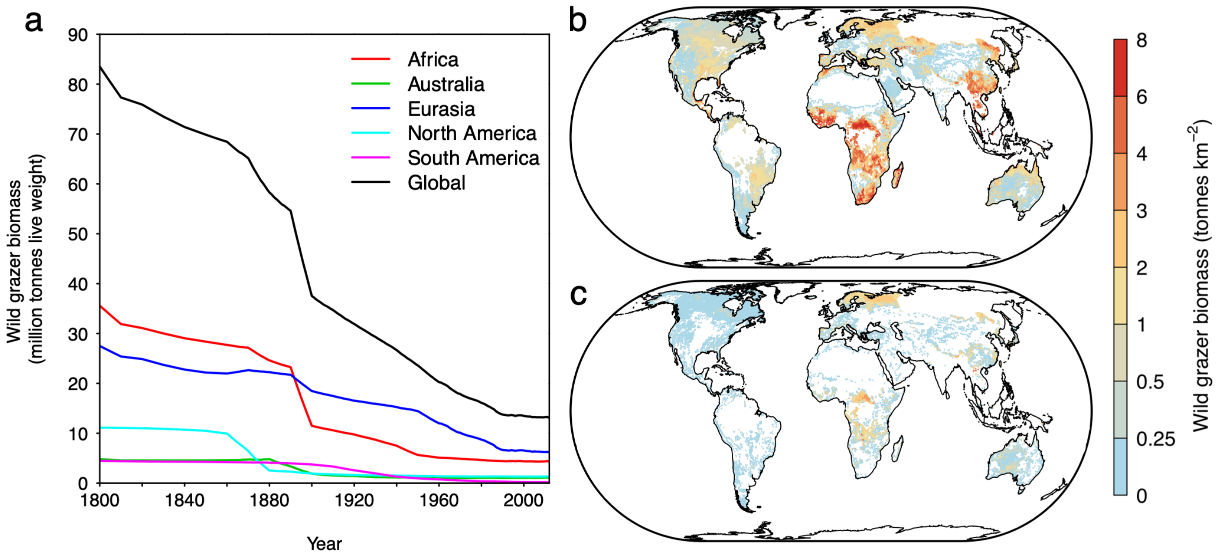

Supplementary Figure 3. The distribution of wild grazer biomass in time and space. (a) Temporal evolution of global and regional total wild grazer biomass (unit: million tonnes live weight), and wild grazer biomass density in (b) 1800 and (c) 2000 calculated in this study. To present the total wild grazer biomass of each grid cell, the wild grazer biomass density in (b) 1800 and (c) 2000 is expressed as tonnes of wild grazer live weight biomass per km^2 of total land in each grid cell (including grassland and all other ecosystems), which is mainly caused by human land use. Constant wild grazer density over grassland is assumed from 1800 to 2012, except for two major events where major reductions in wild grazers were caused by factors other than human land use (Supplementary Methods 1, Section *"Reconstructing the history of wild grazer density and grazed area"*.).

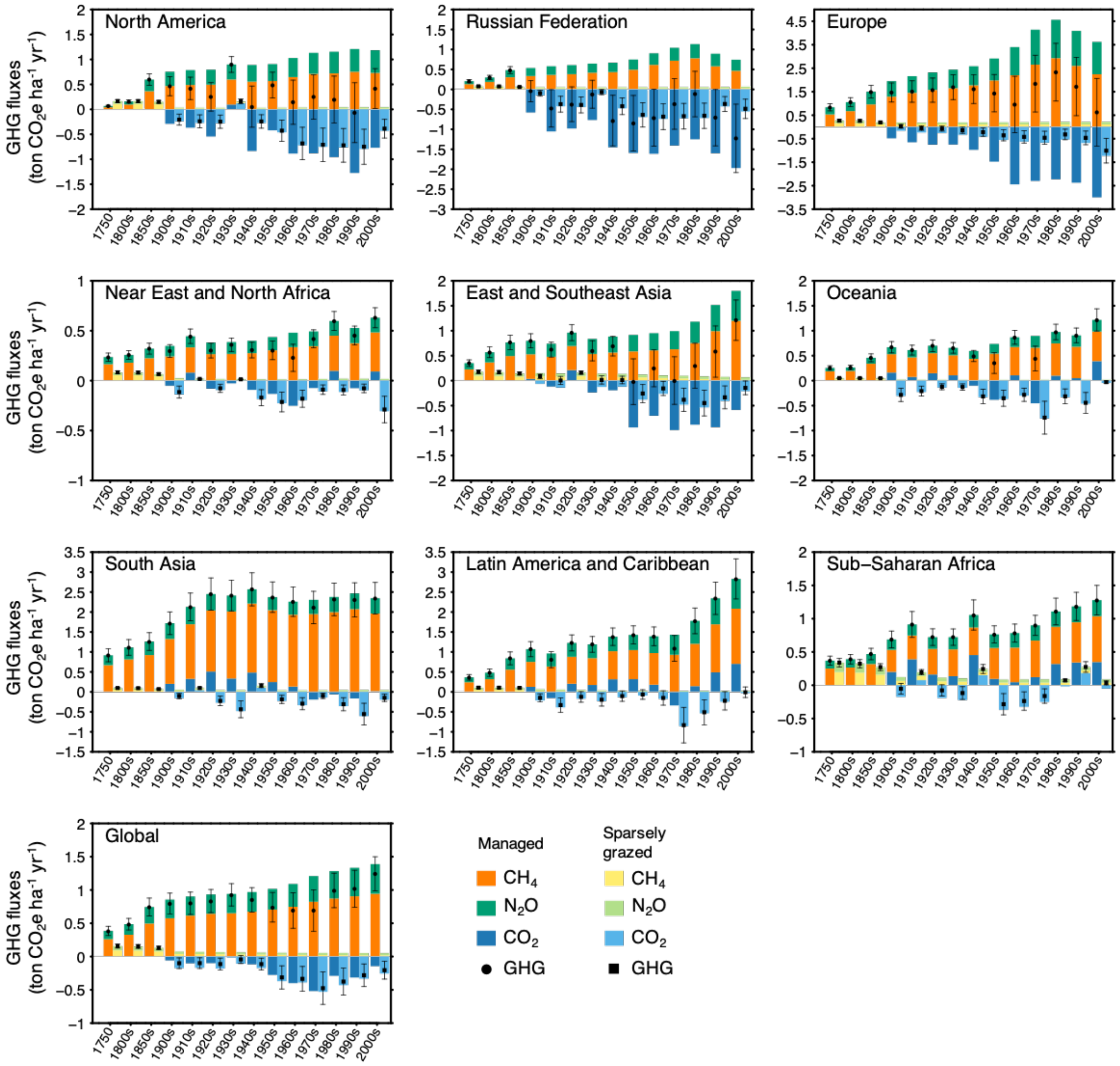

Supplementary Figure 4. The intensity of the decadal greenhouse gas (GHG) fluxes over intensively managed and sparsely grazed grassland in different regions since 1750. The intensity (in units of tonne CO_2e ha^{-1} yr^{-1}) is calculated as the regional GHG fluxes divided by the regional area of intensively managed and sparsely grazed grassland respectively. Land-use change emissions are not included in this intensity calculation. Light and dark blue bars represent CO_2 fluxes from managed and sparsely grazed grassland, respectively; orange and yellow represent CH_4 fluxes from managed (domestic livestock) and sparsely-grazed (wild grazers) grassland, respectively; light green and dark green represent N_2O fluxes from managed and sparsely grazed grassland, respectively; and pink and purple represents land-use change emissions related to grassland from deforestation to pasture and from conversion of grassland to cropland respectively. Black dots and squares (and their error bars) indicate net GHG balance (and its 1-sigma uncertainty; excluding land-use change emissions) over intensively managed

and sparsely grazed grassland respectively. Regions are classified following the definitions in the FAO Global Livestock Environmental Assessment Model (GLEAM; http://www.fao.org/gleam/en/). We combined western and eastern Europe as “Europe” to avoid any individual region being too small.

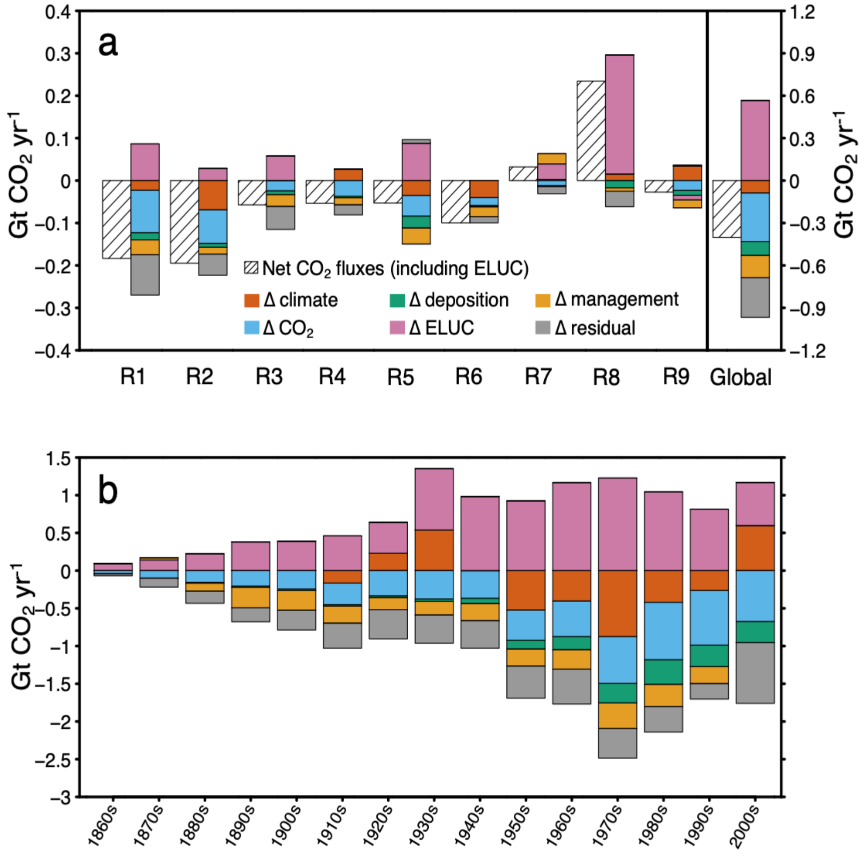

Supplementary Figure 5. (a) The effect of each driver on the net CO_2 fluxes of grassland in each region and the global average for the period 1860-2012, and (b) the global effects for each decade since the 1860s. Positive values indicate net CO_2 source or the driver(s) contribute to CO_2 emission, and negative values indicate net CO_2 sink or the driver(s) contribute to CO_2 sink. R1-R9 indicate the nine regions used in this study, which are North America (R1), Russian Federation (R2), Europe (R3), Near East and North Africa (R4), East and Southeast Asia (R5), Oceania (R6), South Asia (R7), Latin America and Caribbean (R8), and Sub-Saharan Africa (R9) respectively. Bars with hatching indicate the net regional/global CO_2 fluxes of grassland averaged for the period 1860-2012; Δclimate, ΔCO_2, Δdeposition, ΔELUC, Δmanagement are the individual effects of climate change, rising CO2 concentration, changes in atmospheric nitrogen deposition, land-use changes related to grassland, and grassland management together with the extirpation of wild grazers, respectively, to the net CO_2 fluxes for grassland. The sum of individual effects can be less than, or more than, the effect of all the factors taken together, due to nonlinear interactions, and the residual is defined as Δresidual.

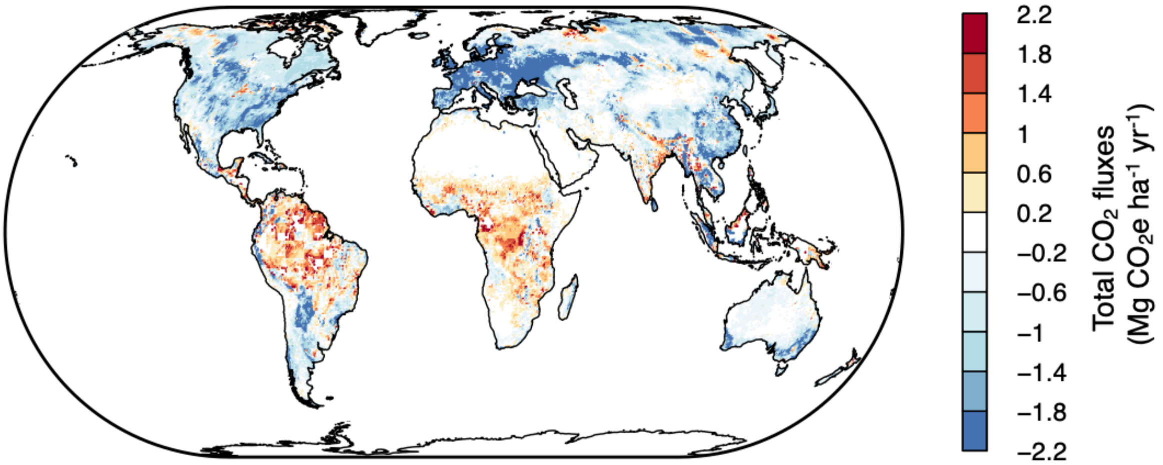

Supplementary Figure 6. Global distribution of grassland carbon balance for the period 1981-2010. Negative values indicate carbon sinks and positive values indicate carbon sources. Land-use change emission related to grassland (CO_2 E_{LUC}) is not included here.

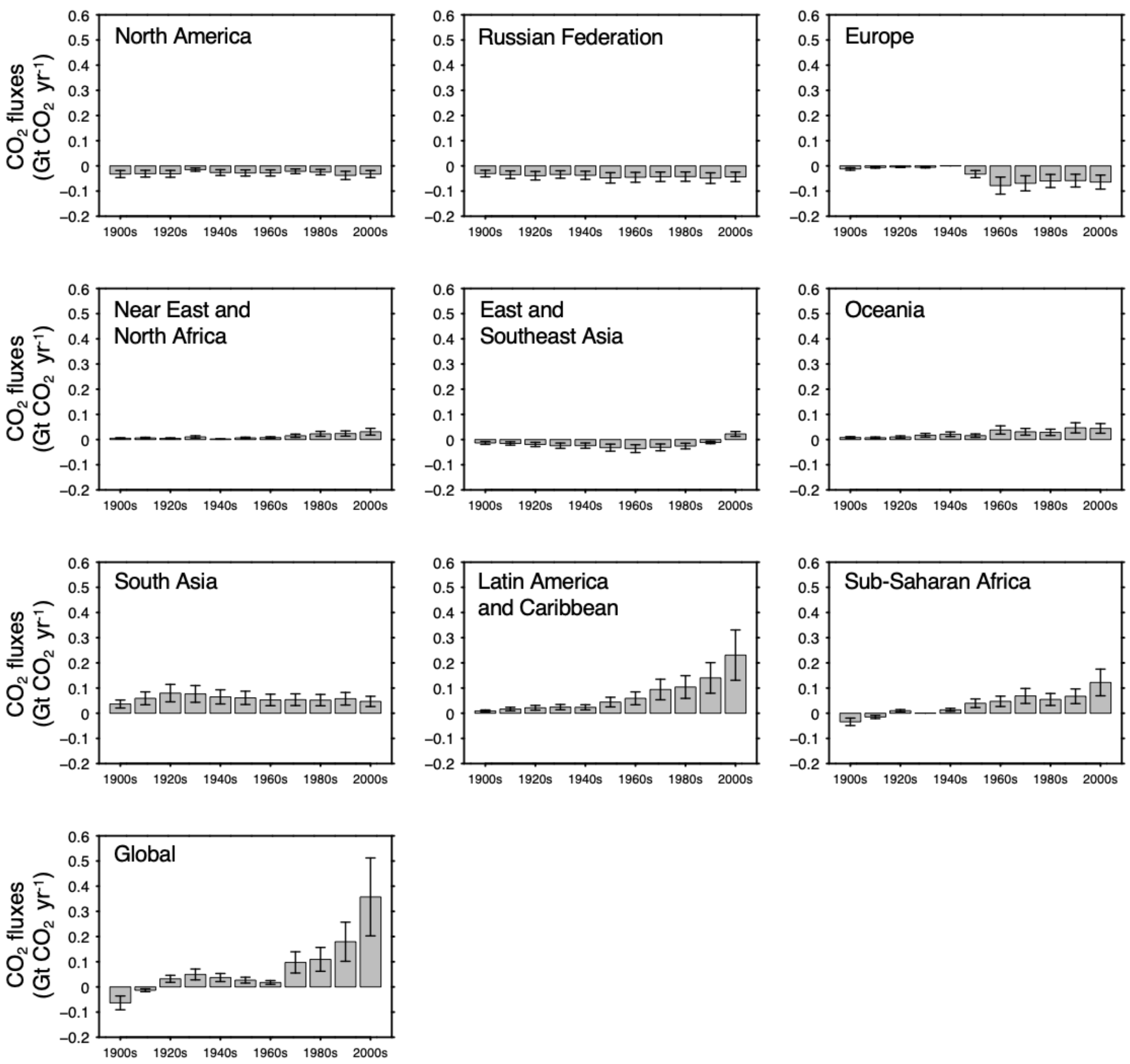

Supplementary Figure 7. The decadal differences between the regional grassland CO_2 fluxes from simulations with (*E4*) and without ($E_{nomanage}$) accounting for wild grazers and grassland management. Positive values indicate that accounting for grassland management reduces net CO_2 sink or enhances net CO_2 source. Black error bars indicate 1-sigma uncertainty of the CO_2 fluxes. Regions are classified following the definitions in the FAO Global Livestock Environmental Assessment Model (GLEAM; http://www.fao.org/gleam/en/). We combined western and eastern Europe as "Europe" to avoid any individual region being too small. $E_{nomanage}$ is a simulation with the same set-up as E1 to E4 (Supplementary Table 1), but does not account for wild grazers and grassland management practices such as grazing, mowing, and nitrogen fertilization.

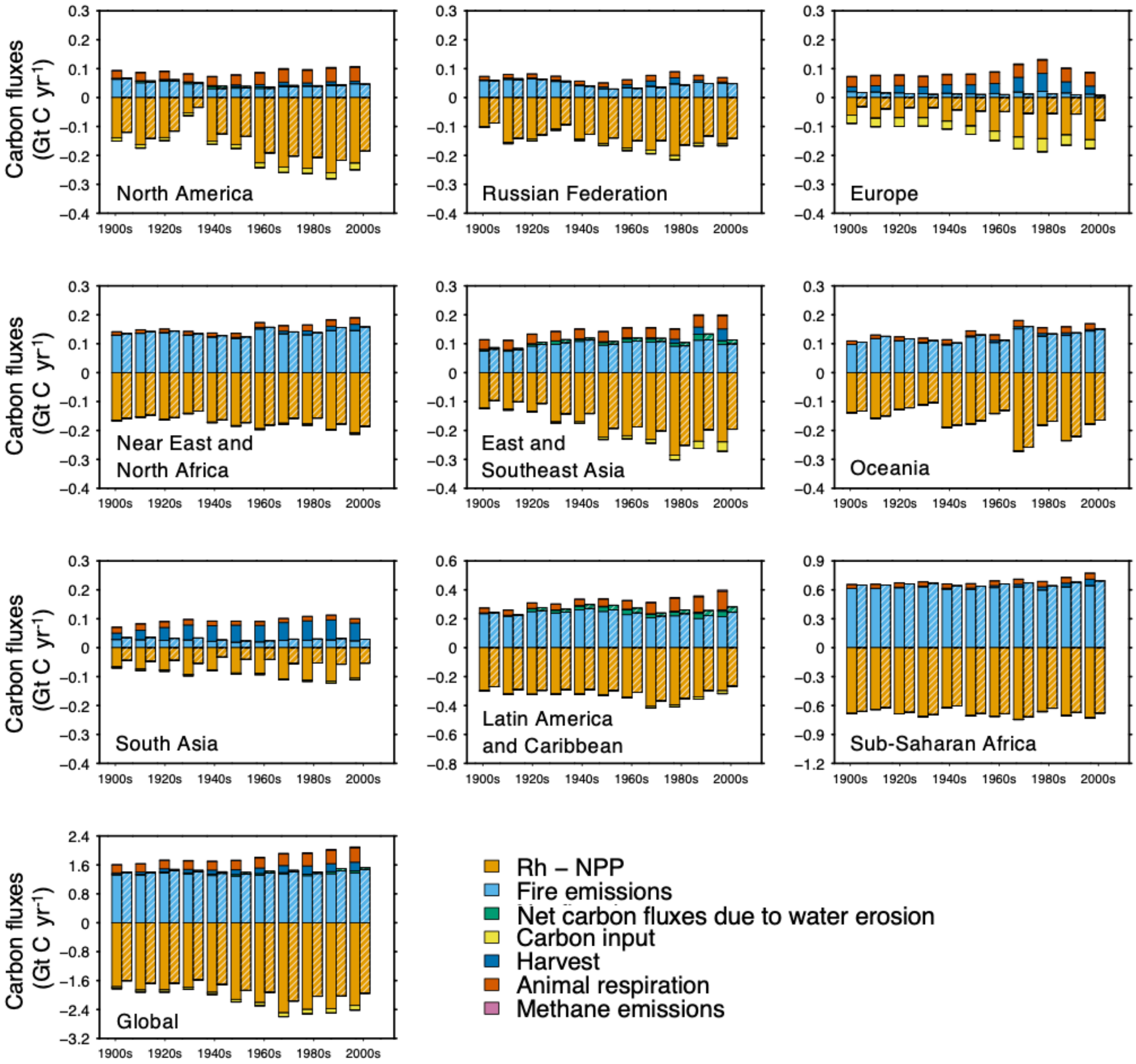

Supplementary Figure 8. The decadal carbon budget and its components for regional grassland from simulations with (*E4; solid bars*) and without (*$E_{nomanage}$; bars with hatching*) accounting for wild grazers and grassland management. Solid bars indicate the carbon budget from simulations with wild grazers and grassland management, which include fluxes like carbon input, harvest, animal respiration and methane emissions caused by wild grazers and grassland management; while bars with hatching indicate the carbon budget from simulations without accounting for wild grazers and grassland management. Negative values indicate a C sink, and positive values indicate a C source. NEE is net ecosystem exchange of grassland ecosystem calculated by Supplementary Equation 26. Rh is the heterotrophic respiration, NPP is the net primary productivity, carbon input is the carbon fluxes into the system through organic fertilizers. Regions are classified following the definitions in the FAO Global Livestock Environmental Assessment Model (GLEAM; http://www.fao.org/gleam/en/). We combined western and eastern Europe as "Europe" to avoid any individual region being too

small. $E_{nomanage}$ is a simulation with the same set-up as E1 to E4 (Supplementary Table 1), but does not account for wild grazers and grassland management practices such as grazing, mowing, and nitrogen fertilization.

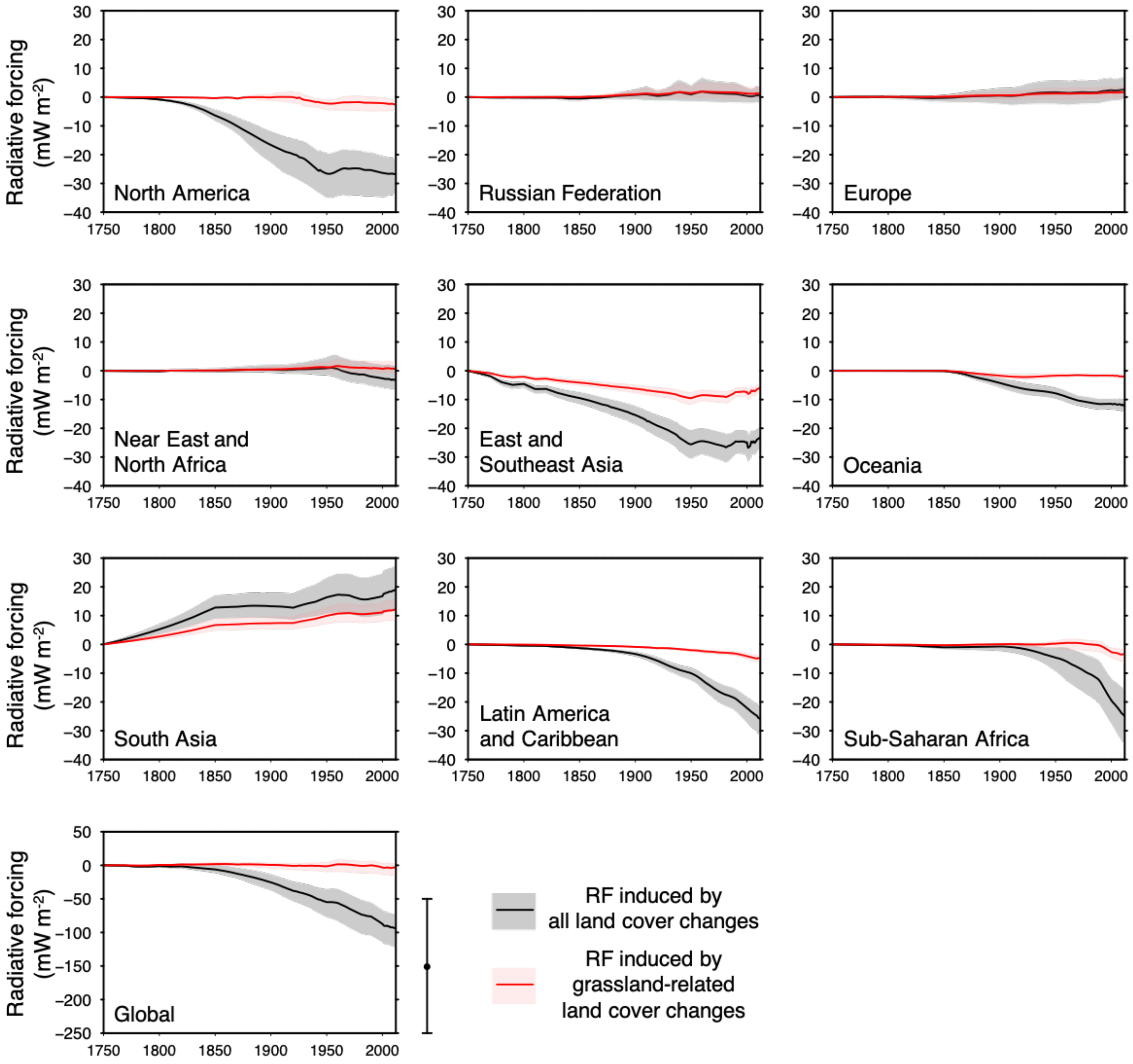

Supplementary Figure 9. The radiative forcing (RF) from albedo change due to grassland-related (red lines and pink-coloured area) and all land-cover change (black lines and grey-coloured area) since 1750. The thick coloured lines indicate the mean of the ensemble of simulations (see Supplementary Methods 2, Section *"Radiative forcing from albedo change due to grassland-related land-cover change"*), and the coloured area its 5th to 95th percentiles. The black dot and its uncertainty (-0.15 ± 0.10 W m-2) on the right hand side of the "Global" estimates are the IPCC estimates of radiative forcing from albedo change due to all land use change and its 90% uncertainty range[120].

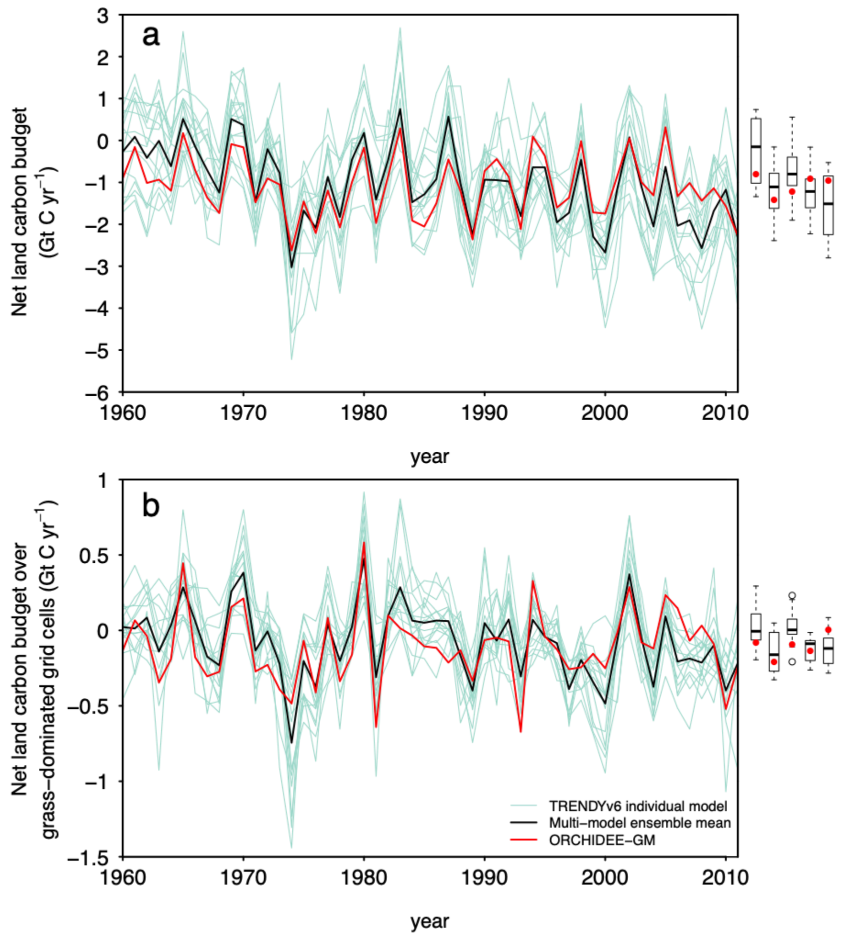

Supplementary Figure 10. Comparison of (a) net land carbon budget, and (b) budget over grass-dominated grid cells derived from the TRENDY models (green, individual model output; black, their multi-model ensemble mean), and that estimated by ORCHIDEE-GM v3.2 in this study (red). Net land carbon budget is the total land CO_2 exchange between the atmosphere and the terrestrial biosphere. The individual TRENDY model simulations with both estimates of residual land sink and land-use change emissions (i.e., S3 experiments including CO_2, climate and land-use forcings) are used here, including CABLE, CLASS-CTEM, CLM4.5 (BGC), DLEM, ISAM, JSBACH, JULES, LPJ-GUESS, LPJ, LPX-Bern, OCN, ORCHIDEE, ORCHIDEE-MICT, VEGAS, VISIT and SURFEX. Decadal budgets from TRENDY model simulations from the 1960s to the 2000s are presented in the box-and-whisker plot, with the red points showing the decadal budgets simulated by ORCHIDEE-GM. Grass-dominated grid cells are those with grassland covering over 60% of the land area in 2000 according to the land-cover change map used in this study (Supplementary Table 4). Here, negative values indicate a net land carbon sink, and positive values indicate a net land carbon source.

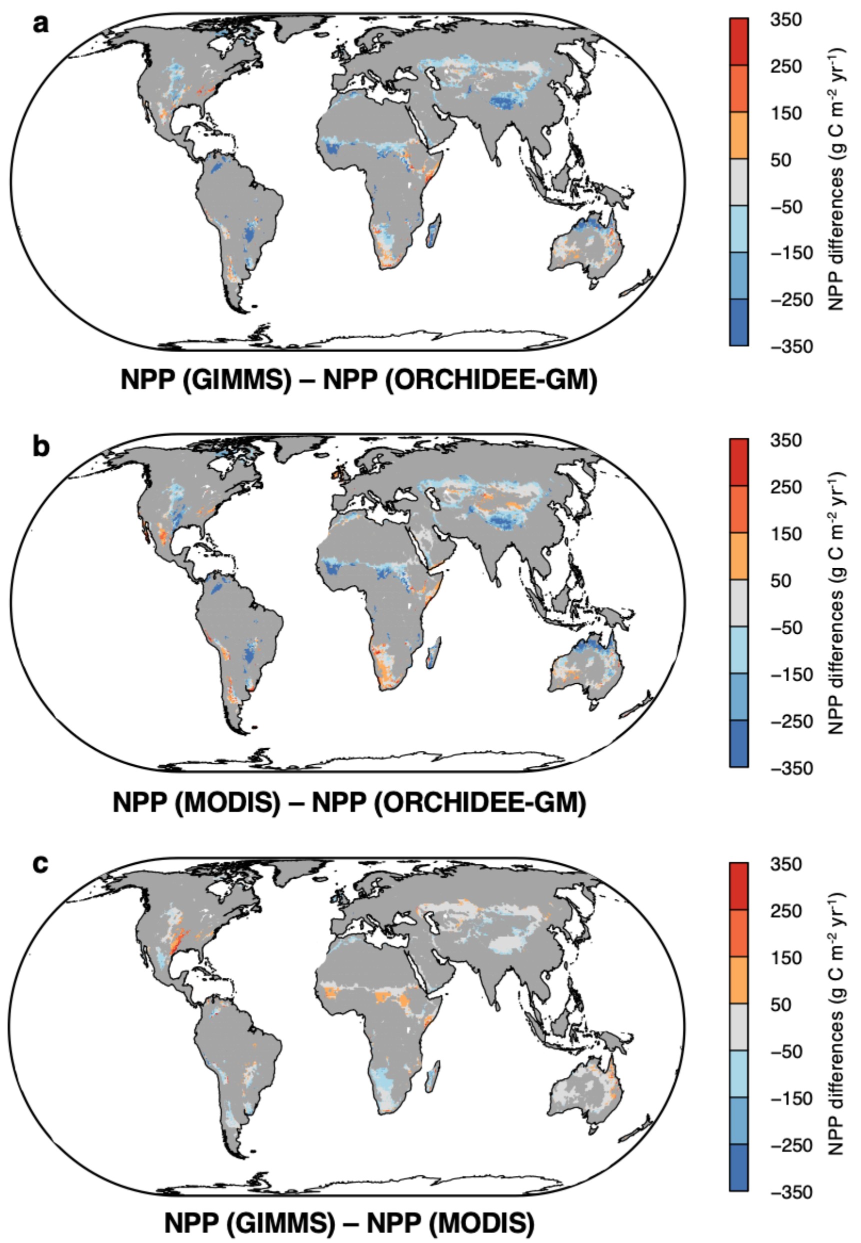

Supplementary Figure 11. Differences between terrestrial net primary productivity (NPP) simulated by the ORCHIDEE-GM v3.2 model and those from two satellite-based models (GIMMS-NPP and MODIS-NPP) over grass-dominated grid cells. MODIS-NPP is derived from the MOD17A3 data set of the Moderate Resolution Imaging Spectroradiometer (version 55 [5,6]) for the period 2000–2013. The extracted annual MODIS-NPP is aggregated to $0.5^{o}\times 0.5^{o}$ spatial resolution to be comparable with model output. GIMMS-NPP is a 30-year global data set of satellite-derived NPP (1982-2011) derived from ref[4]. GIMMS-NPP is constructed using the MODIS NPP algorithm, and driven by long-term Global Inventory Modeling and Mapping Studies (GIMMS) FPAR and LAI data[184]. Grass-dominated grid cells are those with grassland

covering over 60% of land area in 2000 according to the land-cover change map used in this study (Supplementary Table 3). All data were averaged for the common period 2000–2011.

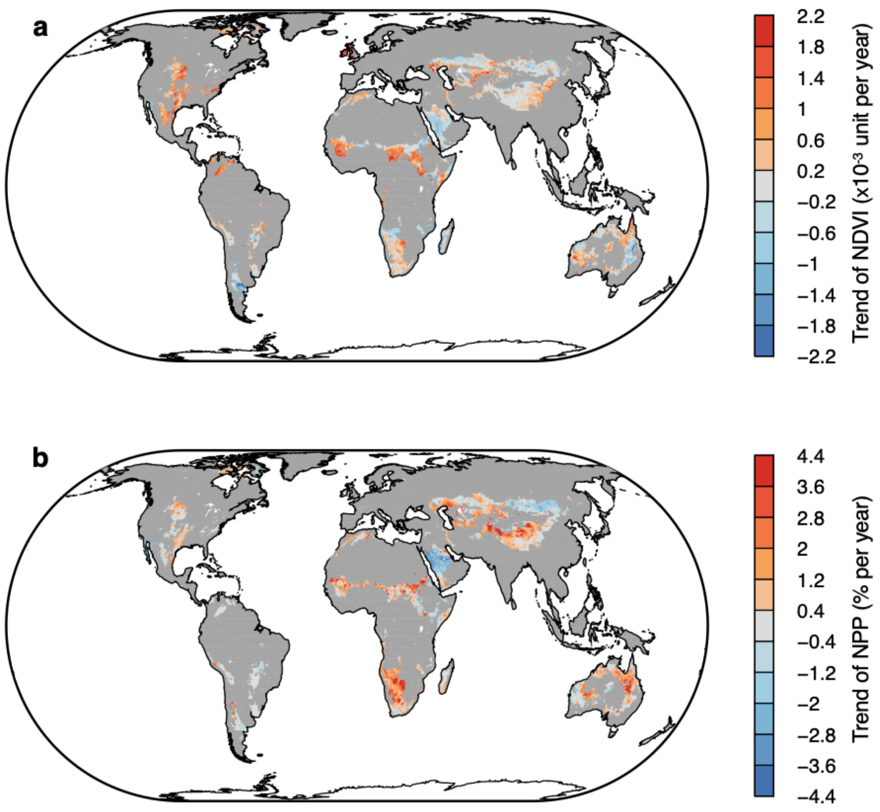

Supplementary Figure 12. Comparison of the spatial pattern between the trend of annual mean GIMMS-NDVI and the trend of annual net primary productivity (NPP) simulated by ORCHIDEE-GM v3.2 during the period 1982–2010 over grass-dominated grid cells. Grass-dominated grid cells are those with grassland covering over 60% of land area in 2000 according to the land-cover change map used in this study (Supplementary Table 4). The NOAA/AVHRR composite GIMMS-NDVI data[9] were used here.

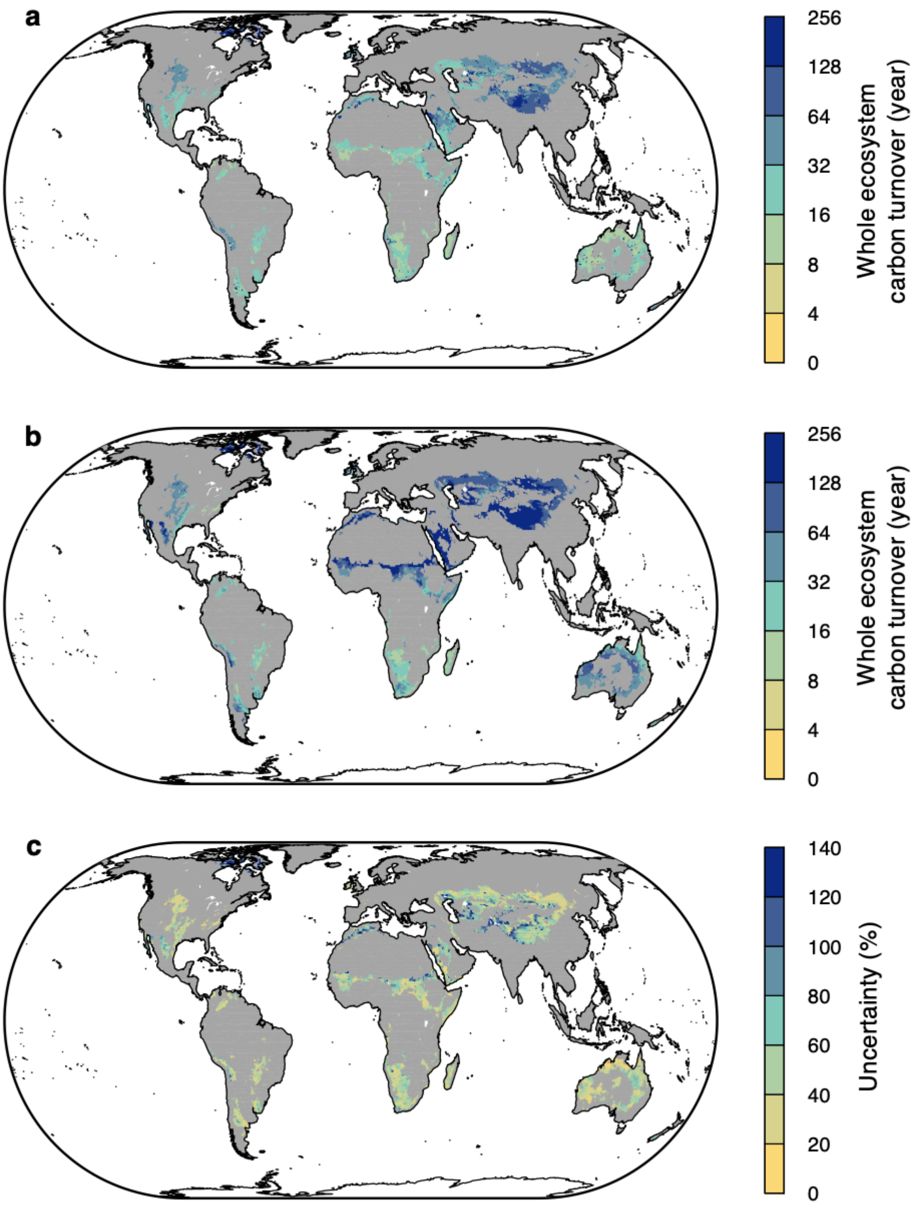

Supplementary Figure 13. Comparison of whole ecosystem carbon turnover times over grass-dominated grid cells. Here, whole ecosystem carbon turnover time is calculated as total soil carbon/NPP (in units of years). (a) Whole ecosystem carbon turnover times derived from total soil carbon and NPP simulated by ORCHIDEE-GM. For comparison, we used six sets of whole ecosystem carbon turnover times derived from the combinations of two satellite-based models of NPP (MODIS-NPP[5,6]; and GIMMS-NPP[4], and three soil carbon stock data sets at 1 metre depth[13] (one is given by SoilGrids[14,15], and two are given by the Harmonized World Soil Database (HWSD[16]) using SOTWIS bulk densities and Saxton bulk densities[17]). Mean whole ecosystem carbon turnover time of the six sets, and their uncertainty (standard deviation; expressed as percentage of the mean value) are shown in subplots (b) and (c) respectively.

Grass-dominated grid cells are those with grassland covering over 60% of land area in 2000 according to the land-cover change map used in this study (Supplementary Table 4).

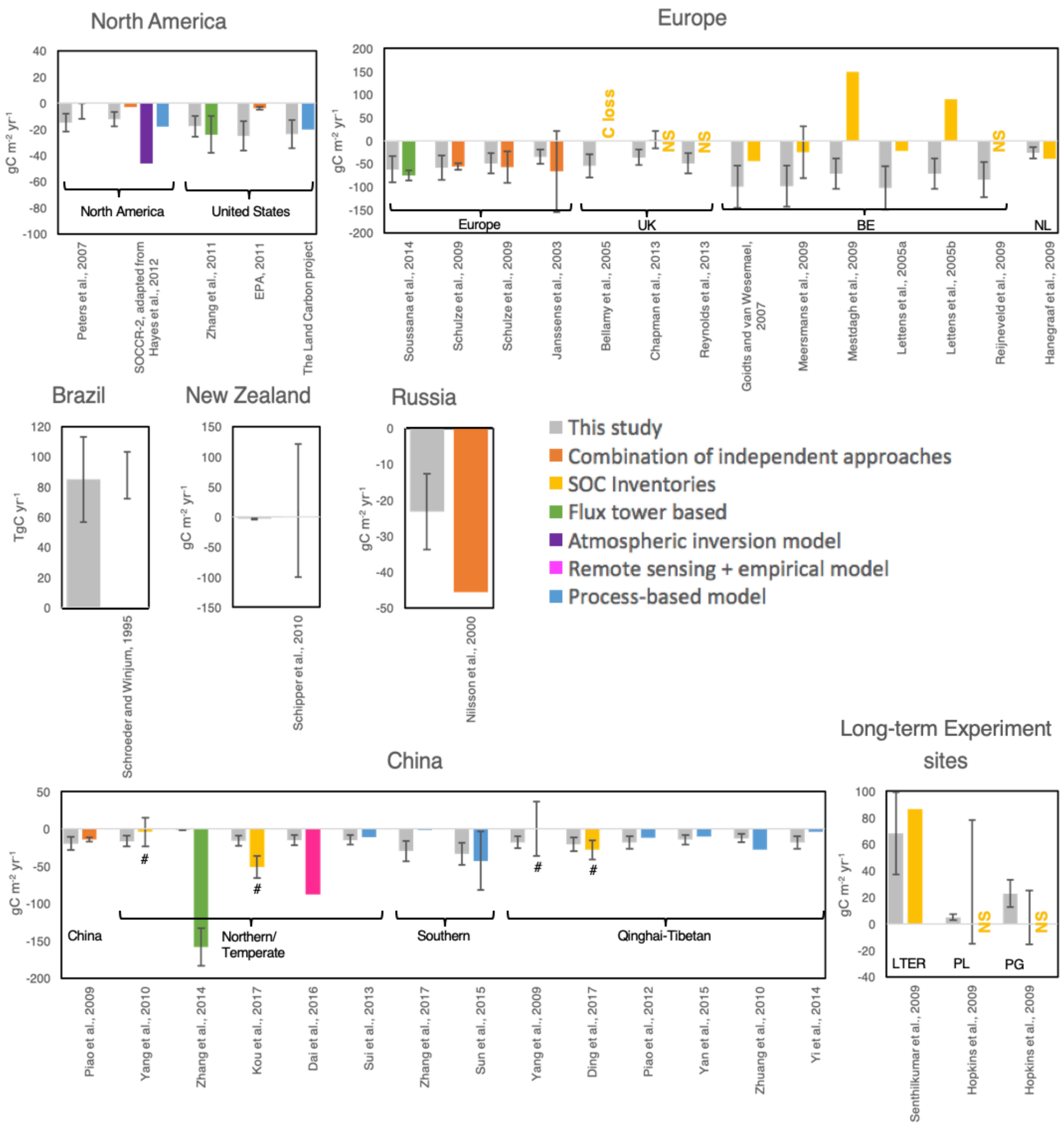

Supplementary Figure 14. Comparison of the grassland carbon budgets estimated in this study against estimates from previous studies. Detailed information on the data used in this figure can be found in Supplementary Table 1. For comparability, all budgets are expressed in units of g C m^{-2} yr^{-1}. A negative value indicates a carbon sink, and a positive value indicates a carbon source. ‘C loss’ indicates a loss of soil carbon is observed; ‘NS’ indicates no significant change in SOC is observed. Error bars indicate the uncertainty of the estimates (expressed as standard deviation), while an error bar with a ‘#’ symbol below indicates that the uncertainty of the estimates is given as 95% confidence interval. UK, United Kingdom; BE, Belgium; NL, Netherlands. LTER, the virgin grassland site at the Kellogg Biological Station’s Long-Term Ecological Research (LTER) site, southwest Michigan, United States; PL, the Palace Leas (PL)

meadow hay plots located in northeast England, United Kingdom; PG, Park Grass (PG) continuous hay experiment located in southeast of England, United Kingdom.

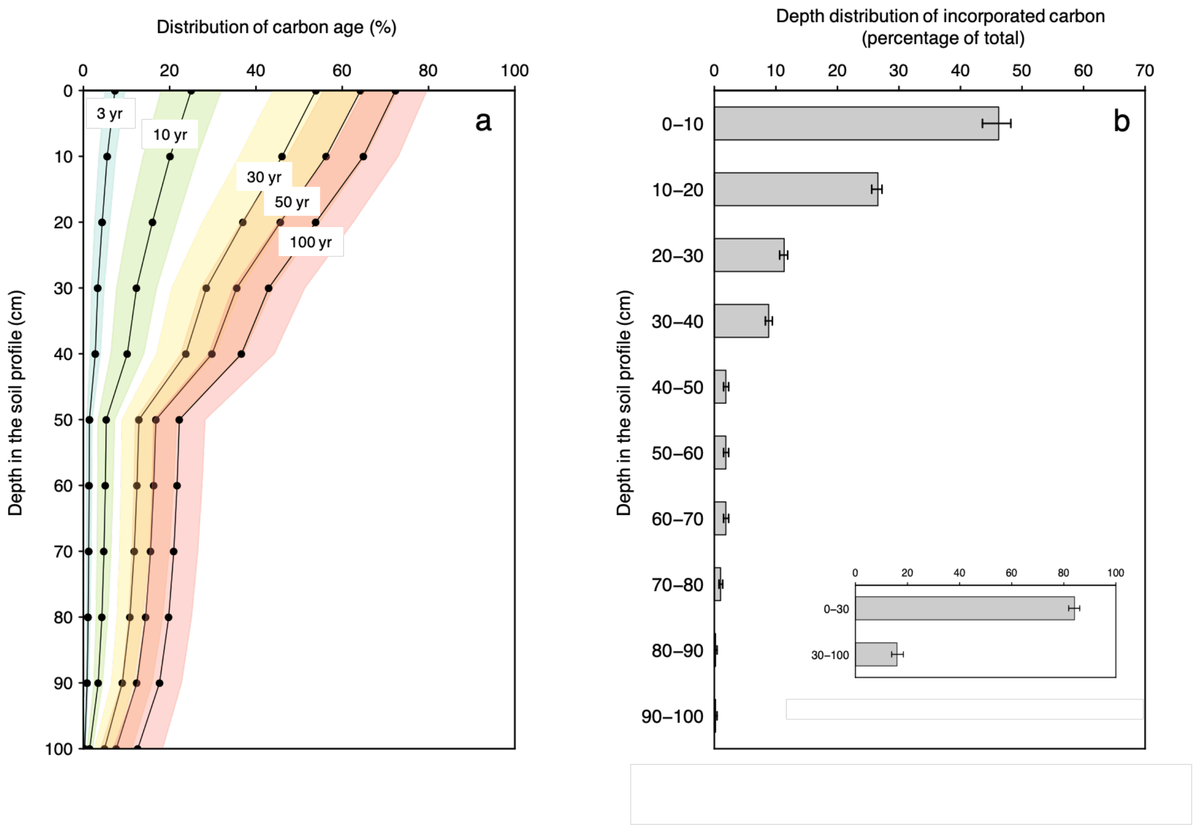

Supplementary Figure 15. (a) carbon age distribution over 32 tropical grassland soil profiles, and (b) depth distribution of the carbon that has been incorporated in soil organic matter between 1965-2012 simulated by ORCHIDEE-GM v3.2. The value in (b) is expressed as a proportion of the total carbon incorporated in the top 100 cm. Error bars indicate the 1-sigma uncertainty from model outputs over the 32 tropical grassland soil profiles.

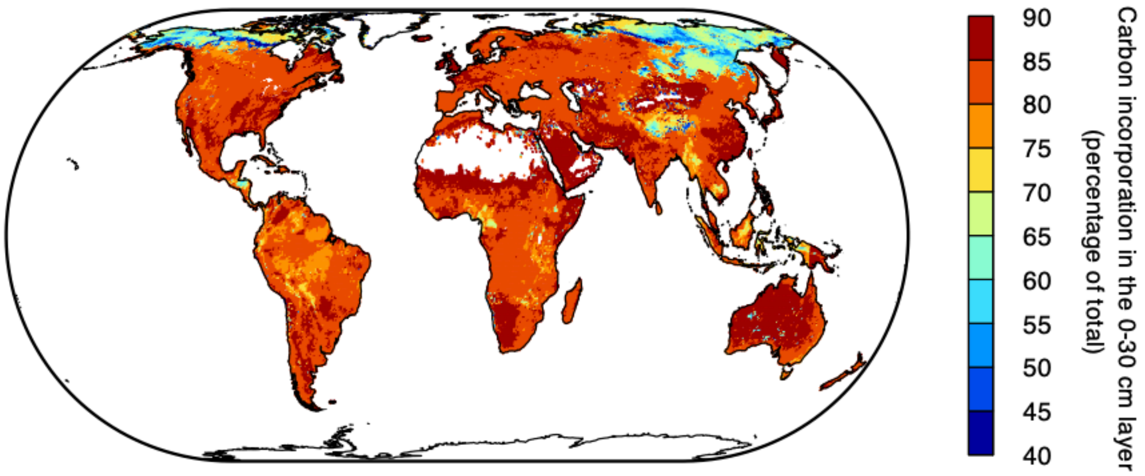

Supplementary Figure 16. Spatial pattern of "recent" carbon that has been incorporated in topsoil (0-30 cm) in grassland between 1965 and 2012 simulated by ORCHIDEE-GM v3.2. The value is expressed as a proportion of the total carbon incorporated in the top 100 cm.

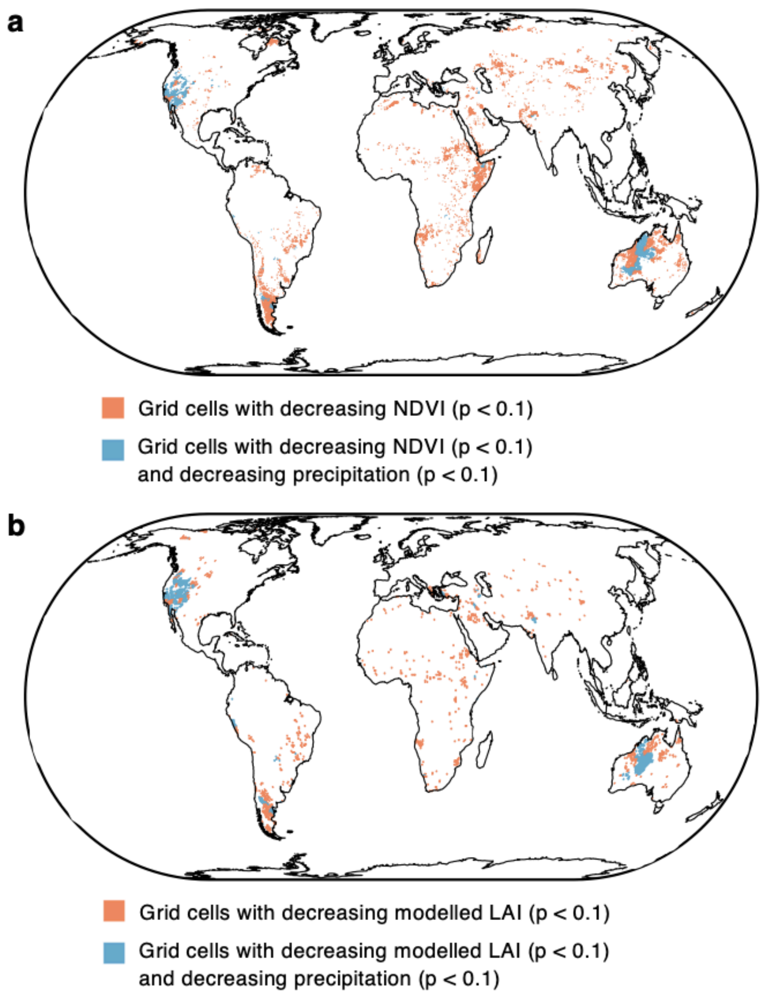

Supplementary Figure 17. Grid cells with significant ($p < 0.1$) decrease in (a) the GIMMS3g NDVI, and (b) the modelled leaf area index (LAI) during the period 1982-1991. Grid cells with decreasing NDVI or modelled LAI and decreasing precipitation are colored blue.

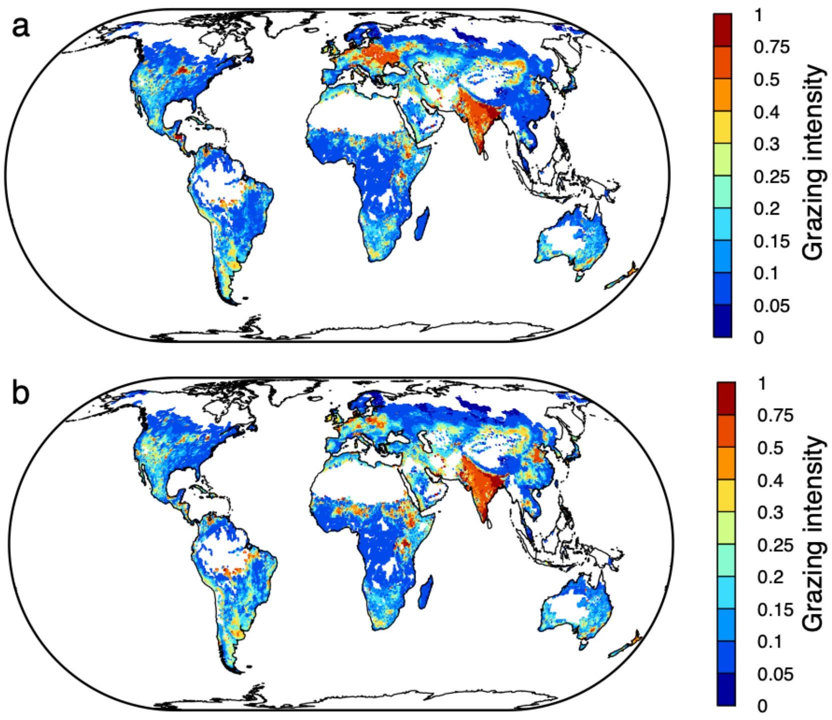

Supplementary Figure 18. The spatial pattern of grazing intensity over managed grassland in (a) 1980s and (b) 2000s from simulation *E4*.

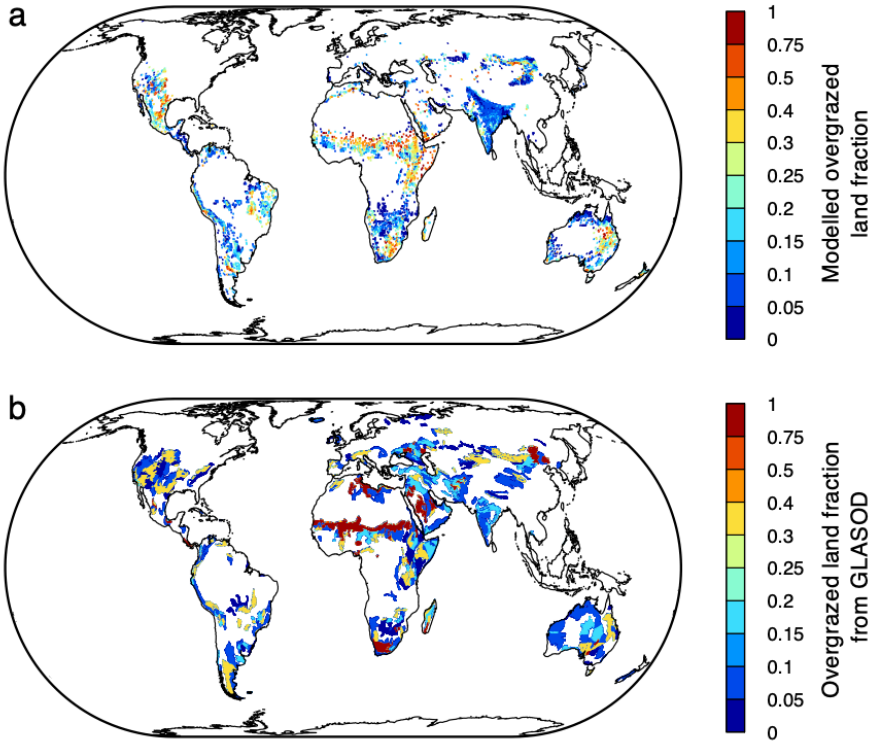

Supplementary Figure 19. Overgrazed land fraction in the 1980s derived from (a) model simulation and from (b) the Global Assessment of Soil Degradation (GLASOD**) database.** Overgrazed grassland in the model simulation is defined as when the modelled NPP of managed grassland ($NPP_{managed}$) is less than 90% of the modelled NPP of natural grassland ($NPP_{natural}$) in the same grid cell.

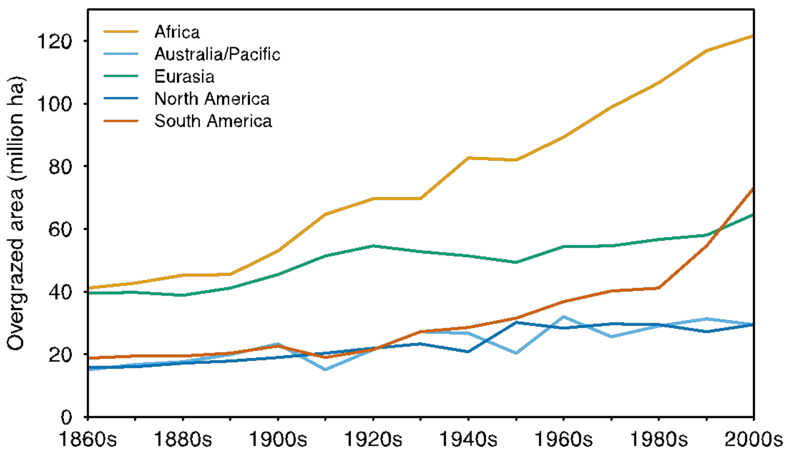

Supplementary Figure 20. Decadal changes in the overgrazed grassland area from model simulation from the 1860s to the 2000s.

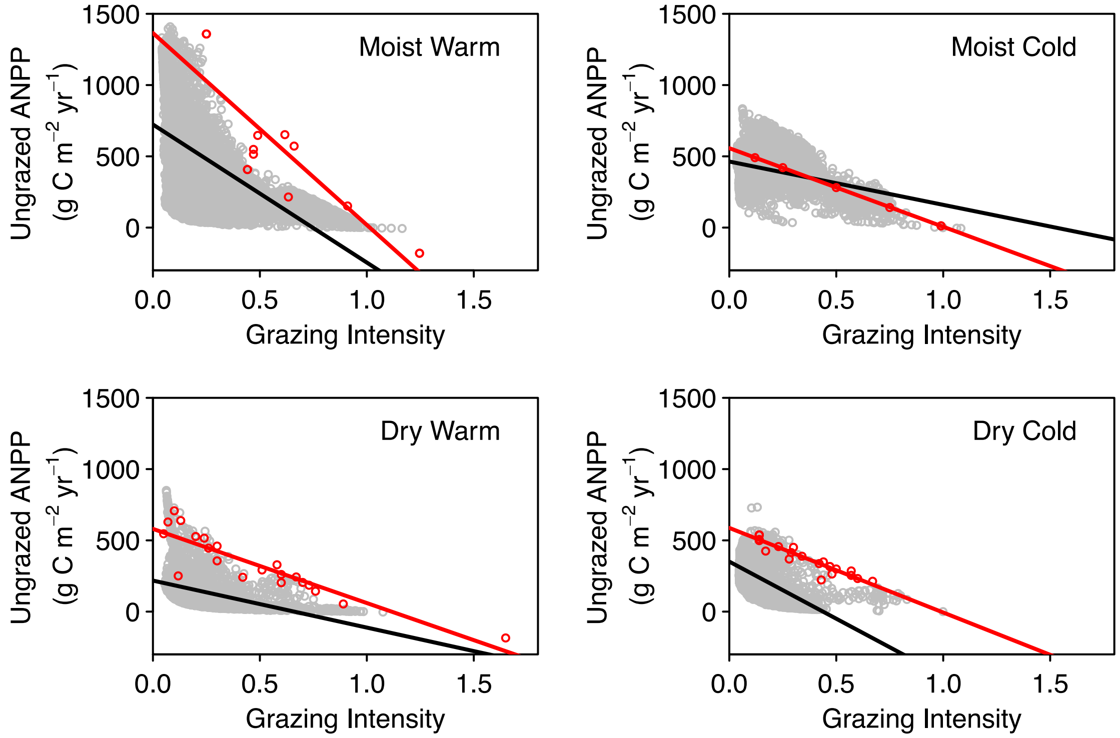

Supplementary Figure 21. Regression of remaining (un-grazed) aboveground net primary productivity (ANPP) to grazing intensity for each climatic zone. Both remaining ANPP and grazing intensity from the simulation *E4* are averaged for the period 1991-2010. Only grid cells with significant annual removal of biomass (grazed or harvested) greater than 10 g C m^{-2} yr^{-1} were used. Red dots are adapted from Figure 4 of ref [77].

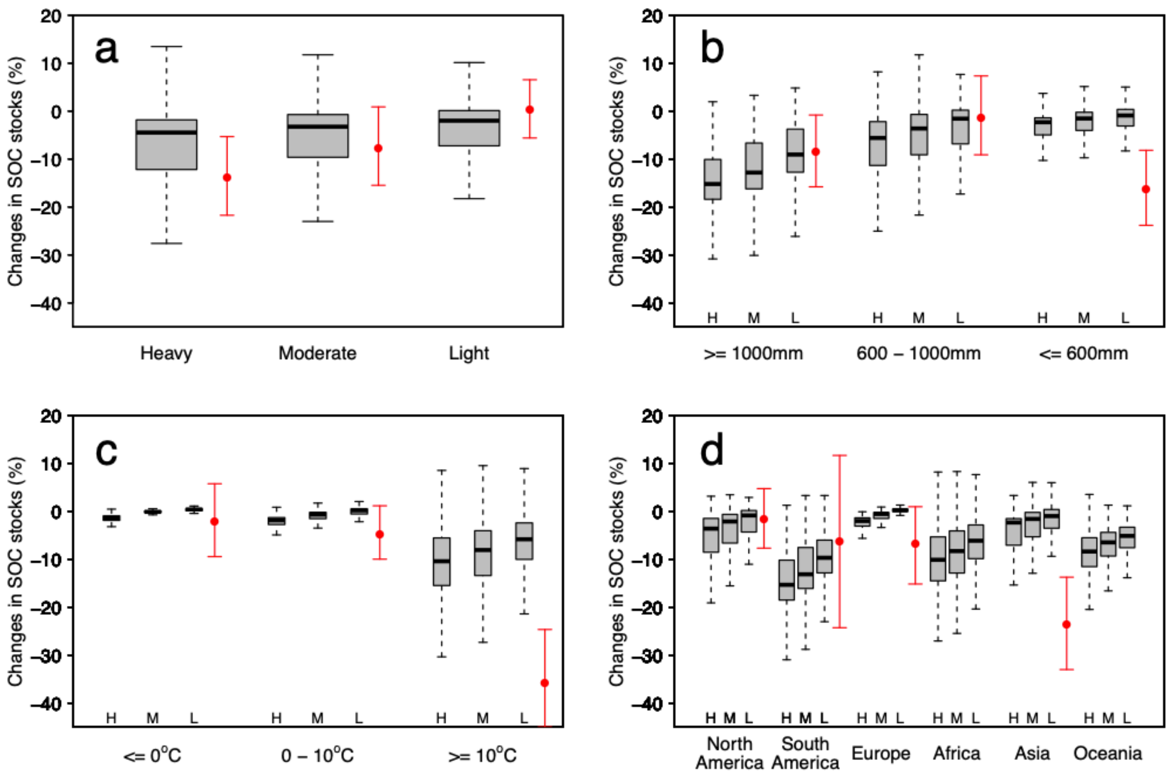

Supplementary Figure 22. Influences of (a) grazing intensity, (b) mean annual precipitation, (c) mean annual temperature on the changes in soil organic carbon (SOC) stocks (0-30 cm soil depth), and (d) the SOC changes for the different continents. The changes in SOC stocks are calculated as the differences between SOC stocks under different grazing intensity after 20 years and those without grazing. The box plots give the 25th (Q1) and 75th (Q3) quantile, the middle of the box is the 50th quantile, and the lower and upper whiskers represent Q1-1.5×IQR and Q3+1.5×IQR respectively with IQR = Q3-Q1. Red dots and error bars are from observations (mean effect sizes with 95% confidence intervals) digitized from Figures 5, 6, 7, 11 of ref [79].

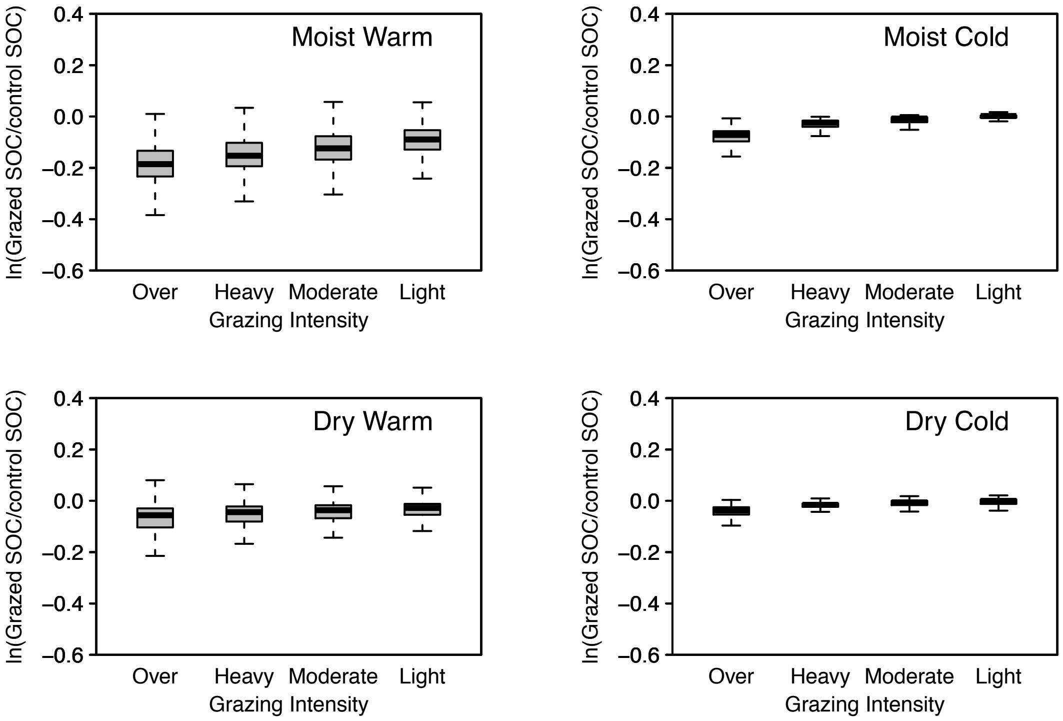

Supplementary Figure 23. Impacts of grazing intensity on soil organic carbon (SOC) stocks (0-30 cm soil depth) under the different climate zones. Impacts are measured using the response ratio ln(RR), which is calculated as the natural logarithm of the ratio of SOC of grazed grassland to SOC of un-grazed grassland. The box plots give the 25th (Q1) and 75th (Q3) quantile, the middle of the box is the 50th quantile, and the lower and upper whiskers represent Q1-1.5×IQR and Q3+1.5×IQR respectively with IQR = Q3-Q1.

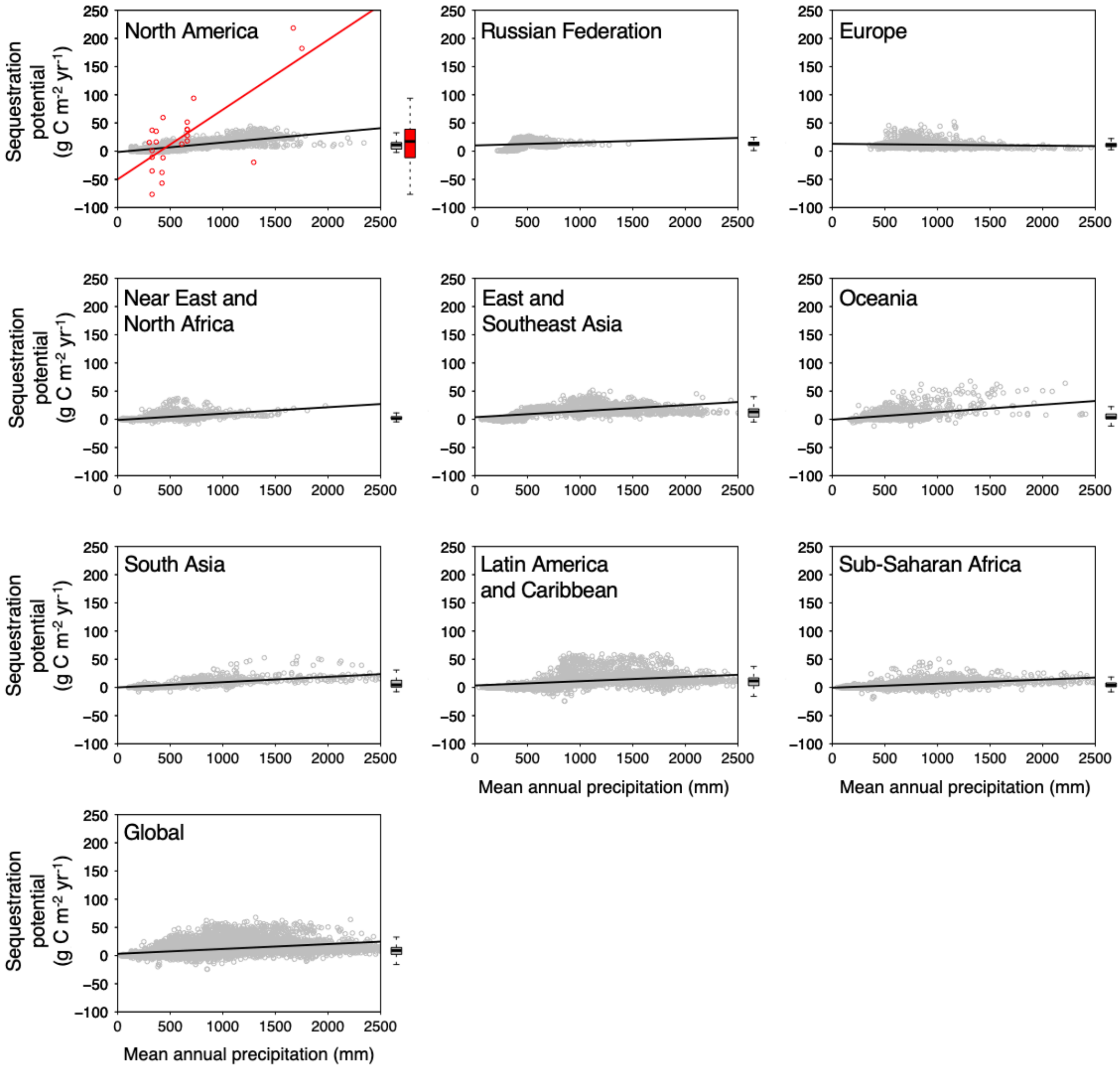

Supplementary Figure 24. Modelled carbon sequestration potential when turning grazing intensity from heavy (grazing intensity GI = 50%) to moderate grazing (GI = 37.5%) and its relation to mean annual precipitation. The box plots give the 25th (Q1) and 75th (Q3) quantile, the middle of the box is the 50th quantile, and the lower and upper whiskers represent Q1-1.5×IQR and Q3+1.5×IQR respectively with IQR = Q3-Q1. Red dots and box are observations digitized from Fig. 3 of ref[78].

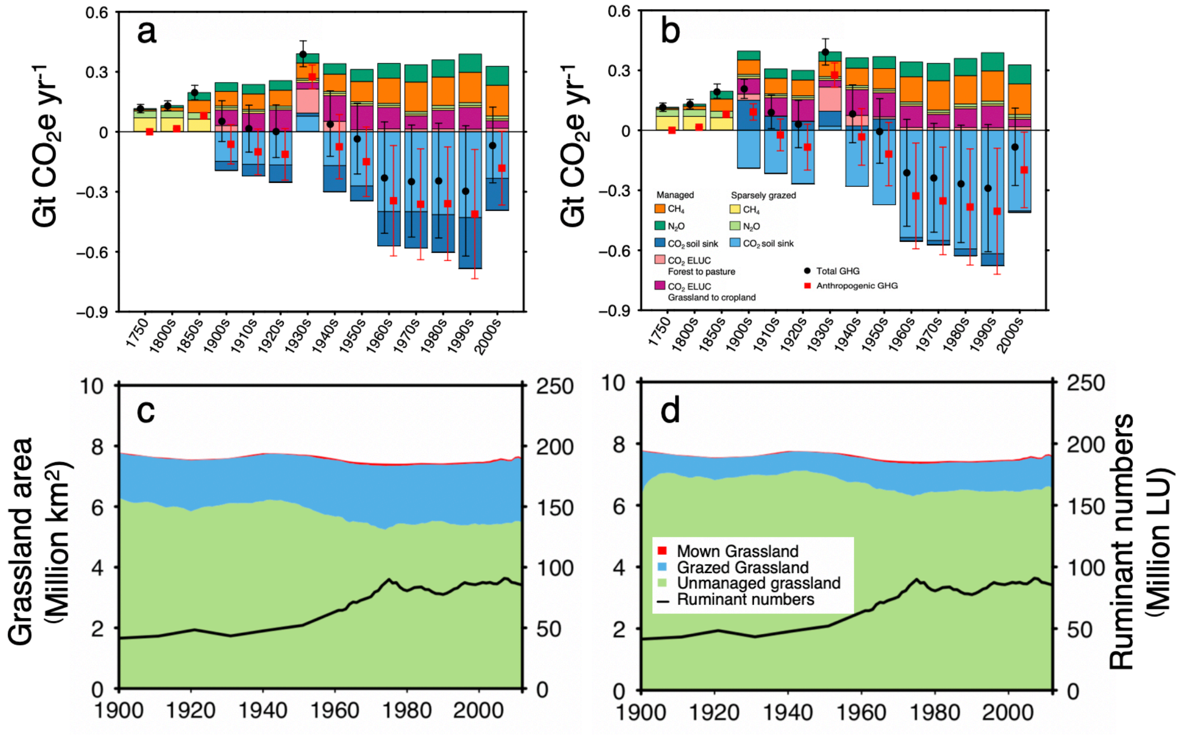

Supplementary Figure 25. The decadal greenhouse gas (GHG) fluxes of grassland (a and b) and historical changes in the area of managed (mown and grazed) and sparsely grazed grassland (c and d) in North America with (a and c) reconstructed grazing intensity and (b and d) test simulations with a higher prescribed grazing intensity of 50% (*heavy grazing*) during the period 1900-1960 and 37.5% (*moderate grazing*) after 1960. Little difference in the current CO_2 fluxes is seen from the legacy of a more intensive grazing in the early 20th century by comparing Fig. a with Fig. b.

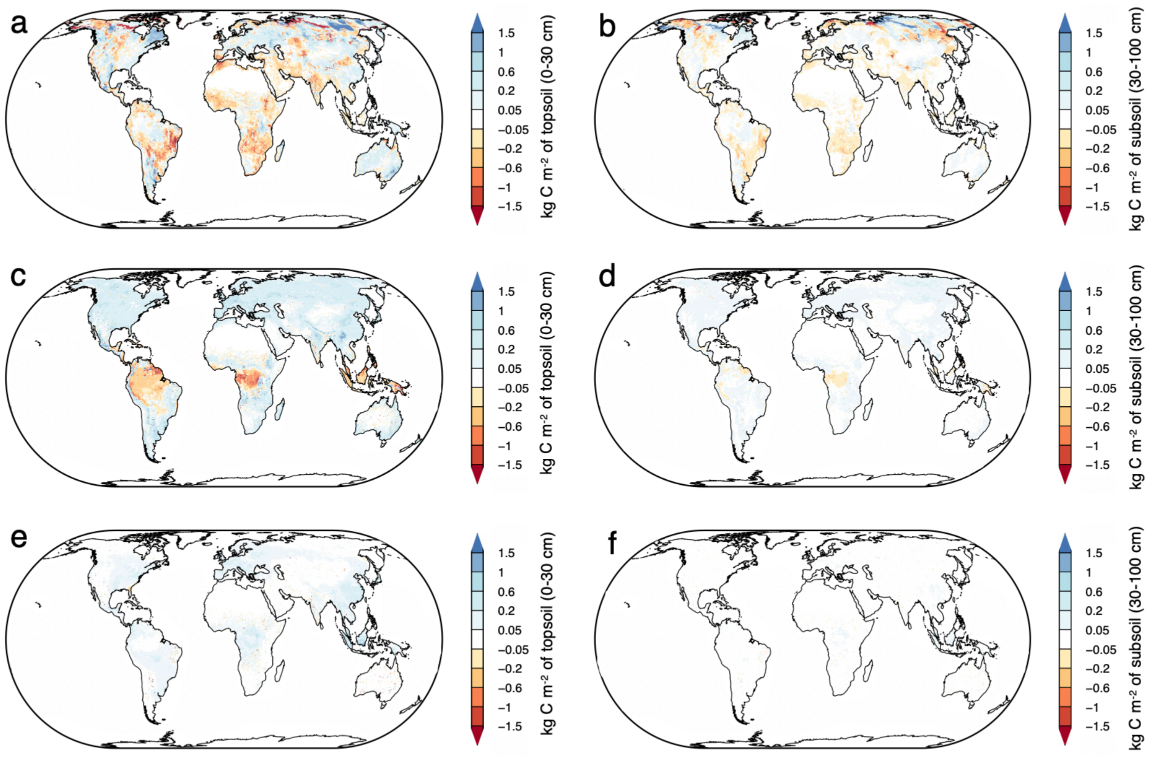

Supplementary Figure 26. Spatial distribution of the effects of climate change (a,b), rising CO_2 (c, d), and atmospheric nitrogen deposition (e, f) since 1860 on soil organic carbon content in topsoil (0-30 cm; a, c, e) and subsoil (30-100 cm; b, d, f), respectively. The effects are expressed as kg C per square metre of grassland integrated over topsoil (0-30 cm) and subsoil (30-100 cm).

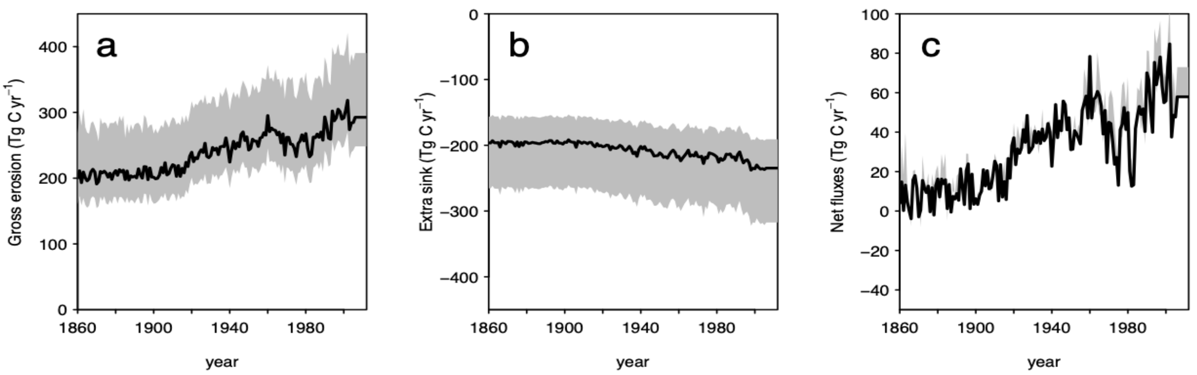

Supplementary Figure 27. Water erosion induced (a) gross soil carbon losses, (b) extra carbon sink due to the reduced soil respiration, and (c) the net carbon fluxes over grassland for the period 1860-2012. Positive values indicate carbon losses, and negative values indicate carbon sinks.

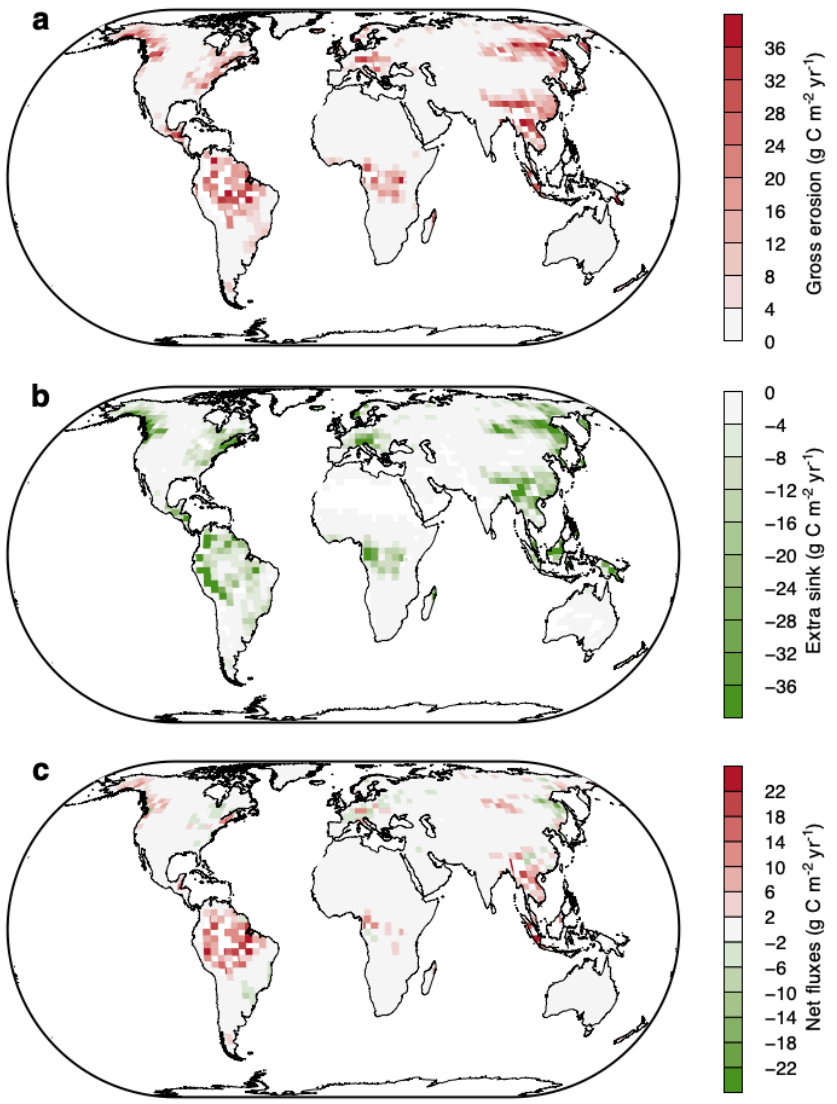

Supplementary Figure 28. The spatial patterns of water erosion induced (a) gross soil carbon losses, (b) extra carbon sink due to the reduced soil respiration, and (c) the net carbon fluxes over grassland for the period 1990-1999. Positive values indicate carbon losses, and negative values indicate carbon sinks.

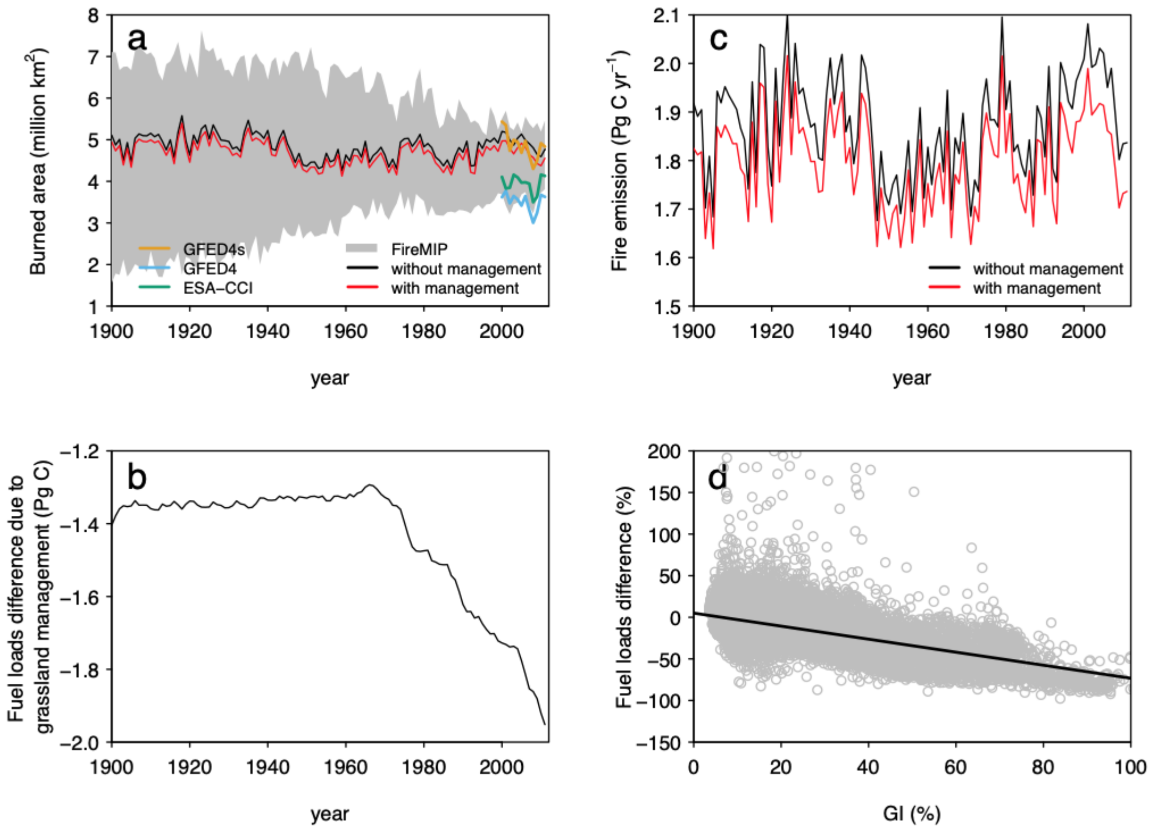

Supplementary Figure 29. (a) Global burned area and (b) fire emissions for the period 1900-2012 simulated by ORCHIDEE-GM v3.2 with and without accounting for grassland management, (c) the simulated changes in fuel loads caused by grassland management for the period 1900-2012, and (d) the simulated response of fuel loads to grazing intensity in the past 20 years (1991-2010).

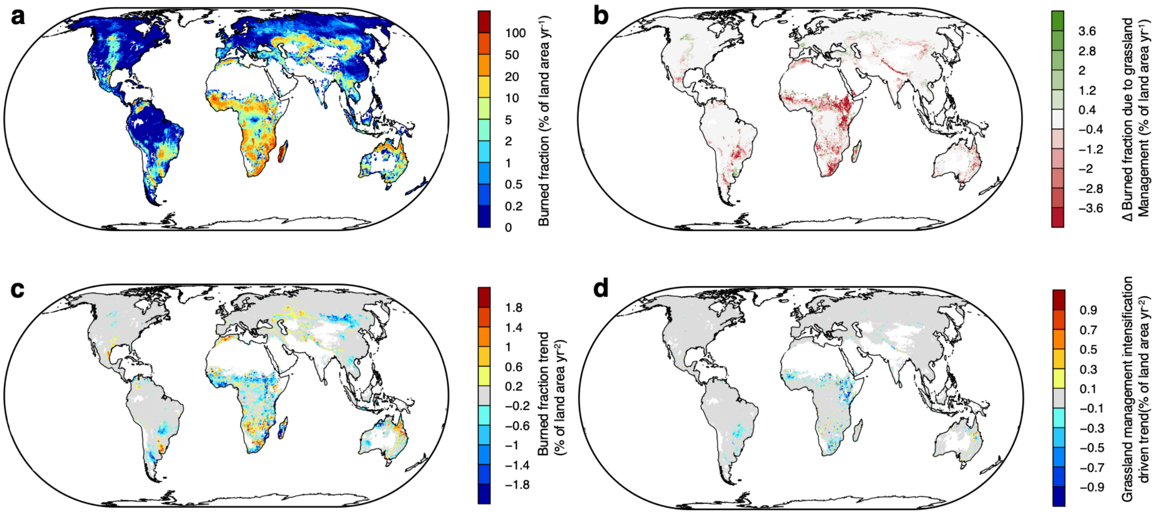

Supplementary Figure 30. (a) Spatial pattern of mean burned fraction during the period 1997-2012 simulated by ORCHIDEE-GM v3.2 accounting for grassland management, (b) the differences in burned fraction (mean of 1997-2012) caused by grassland management, (c) the trend of burned fraction during the period 2000-2012, and (d) the contribution of grassland management intensity change (increase or decrease of livestock numbers) on the trend of burned fraction during the period 2000-2012. Positive differences in (b) indicate that grassland management increased burned area, while negative differences indicate that grassland management decreased burned area.

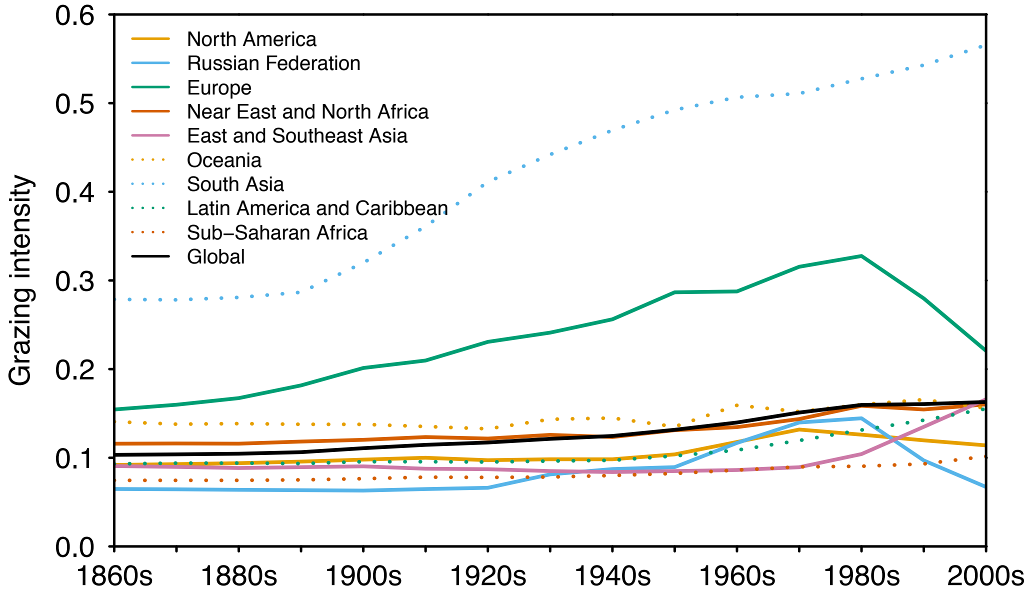

Supplementary Figure 31. Historical changes in the simulated grazing intensity over managed grassland for 1860 and 2012 by region, together with the global total.

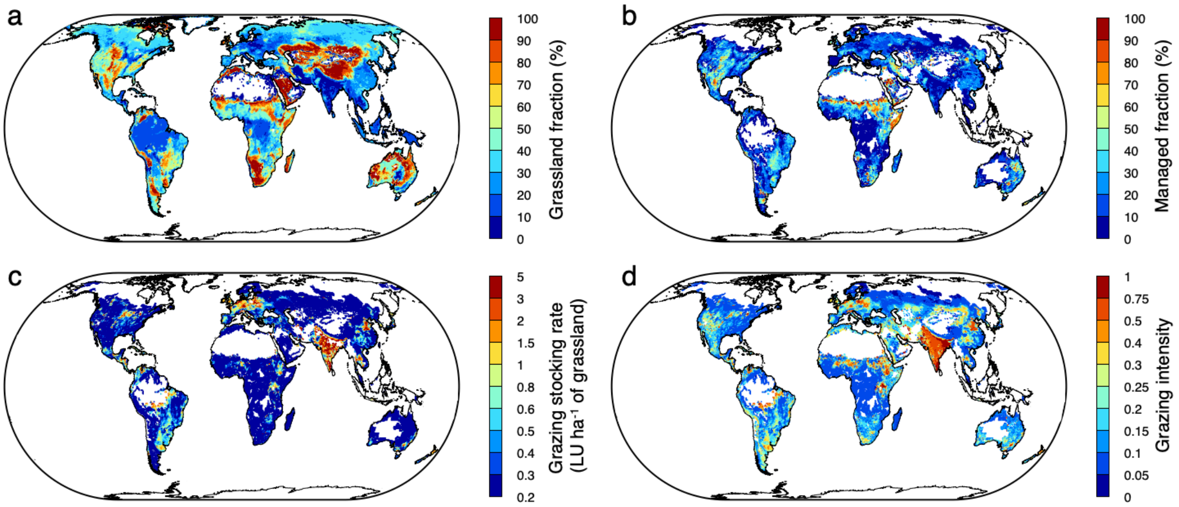

Supplementary Figure 32. The spatial distribution of the grassland fraction in 2010, and the average managed grassland fraction, the grazing stocking rate and the grazing intensity (GI) in 2000s obtained in this study. The grassland fraction in 2010 is derived from the ESA CCI Land Cover product[137] for the year 2010, and is converted to the Plant Functional Type (PFT) map used in ORCHIDEE following the methodology presented by ref[138,139]. The managed grassland fraction, grazing stocking rate is reconstructed as presented in Supplementary Discussion 7. The grazing intensity is simulated by ORCHIDEE-GM v3.2, and is calculated as total harvested and grazed carbon as a ratio of total aboveground NPP (ANPP) over managed grassland following ref[77].

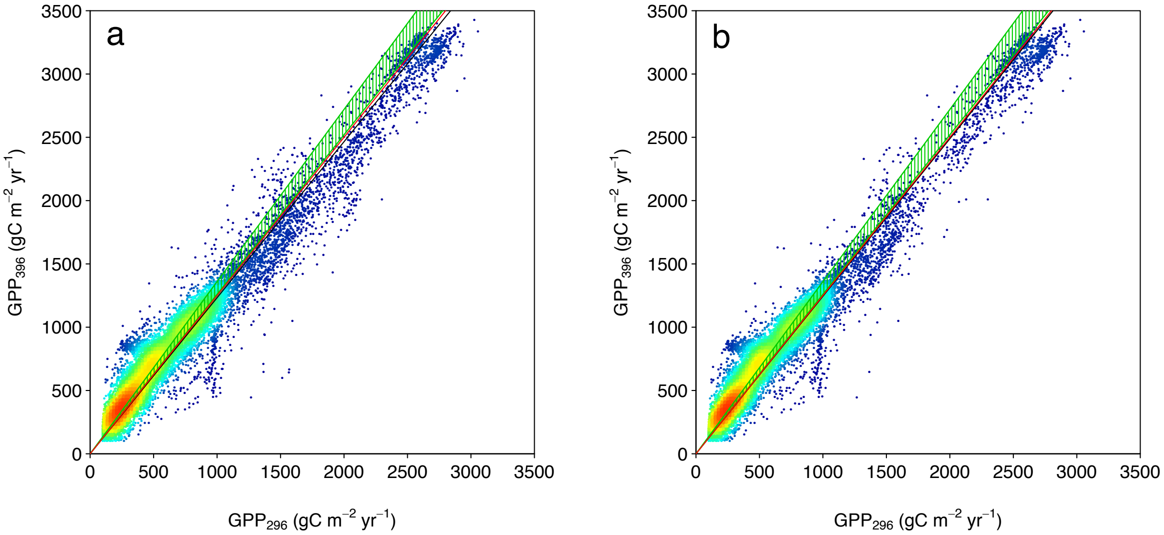

Supplementary Figure 33. Comparisons between pre-industrial gross primary productivity (GPP) with atmospheric CO_2 concentration of 296 ppm (*GPP_{296}*) and current GPP with atmospheric CO_2 concentration of 396 ppm (*GPP_{396}*) for all natural plants (a) and natural C3 plants (b) by ORCHIDEE-GM v3.2. The color scale shows the point density. The ratio between GPP_{396} and GPP_{296} indicates the CO_2 fertilization effects (E_{CO2}). Green hatched areas indicate the observed E_{CO2} from based on long-term atmospheric carbonyl sulfide (COS) records of ref[118]. Red lines indicate the observed E_{CO2} from ref [117]. Black lines indicate the modelled E_{CO2} from ORCHIDEE-GM.

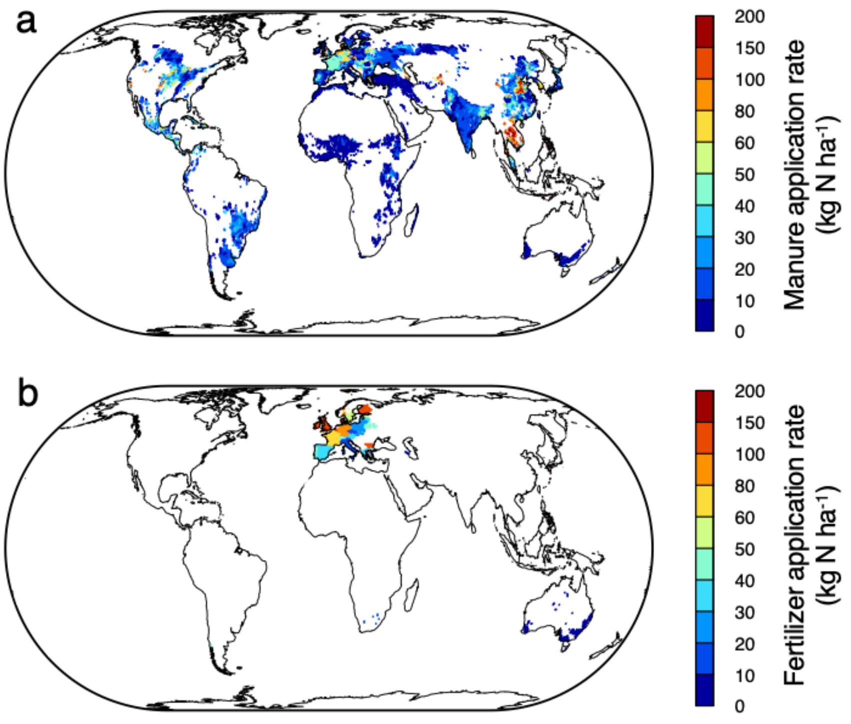

Supplementary Figure 34. The spatial distribution of the manure and fertilizer application rate over managed grassland in 2010 obtained in this study.

Supplementary Table 1. Simulation protocol and input data.

	Simulation E1	Simulation E2	Simulation E3	Simulation E4	Simulation $E_{nomanage}$
Simulation period	Spin-up (20000 years)	1751–1860	1861–1900	1901–2012	Spin-up + 1751–2010
Input data					
Climate forcing[a]	1901-1910 cycled	1901–1910 cycled	1901–1910 cycled	1901–2012	Same as E1 to E4
Atmospheric CO_2 concentration[b]	1860	1860	1861–1900	1901–2012	Same as E1 to E4
Enhanced historic Land-cover change[c]	1860	1860	1861–1900	1901–2012	Same as E1 to E4
Grazing-ruminant stocking density[d]	1860	1860	1861–1900	1901–2012	Not applied
Nitrogen fertilizer application[d]	Not applied	1860	1861–1900	1901–2012	Not applied
Atmospheric-nitrogen deposition[e]	Not applied	1860	1861–1900	1901–2012	Same as E1 to E4
Wild-grazers population density[f]	1860	1860	1861–1900	1901–2012	Not applied

a, climate forcing: the 6-hourly CRU-NCEP[134,135] reconstructed climate data at 0.5° × 0.5° spatial resolution for the period 1901– 2012. Variables used by ORCHIDEE include temperature, rainfall, snowfall, short-wave and long-wave downward radiation, air humidity, and air pressure.

b, atmospheric CO_2 concentration comes from the combination of ice core records and atmospheric observations for 1860–2012 (ref[136] and update)

c, the enhanced historic land-cover change data set is the PFT maps produced in this study incorporating specific historic area changes under different grassland management types and wild grazer occupation (mown, grazed, natural and wild) reconstructed adapted from ref [133] with some improvement described in Supplementary Discussion 7.

d, these input data were described in detail in ref [133].

e, atmospheric-N deposition maps during 1860–2012 were from the IGAC/SPARC Chemistry-Climate Model Initiative (CCMI) N deposition fields[142].

f, wild-grazers population density is reconstructed in this study using the method described in Supplementary Methods 1, Section *“Reconstructing the history of wild grazer density and grazed area”*.

Supplementary Table 2. Comparison of the grassland greenhouse gas fluxes estimated in this study with those from previous studies. A negative value indicates a GHG sink, and a positive value indicates a GHG source. Values are expressed as mean ± standard deviation, while values in brackets indicate 95% confidence interval of the measurements. The grassland carbon budget estimates from previous studies focus on fluxes from grassland without accounting for deforestation for pasture. Thus, the grassland net CO_2 fluxes ($F_{CO_2\text{-}C}$ as in Supplementary Equation 25) excluding land-use change emissions from this study are used here for comparison of CO_2 fluxes. The only exception is the estimate for Brazil. We combined the grassland net CO_2 fluxes and the emissions from deforestation for pasture to be comparable with Schroeder and Winjum (1995). The studies used for estimating the relative uncertainty of the simulated grassland CO_2 fluxes are denoted with a '#' symbol in the "Reference" column.

GHG Fluxes	Globe/Region/Country/Site	Period	This study	Grassland area from this study	GHG estimates from literature	Grassland area from literature	Methods used in literature	Reference
**CO_2**								
	World	Around 1990[a]	–491 ± 223 Tg C yr^{-1}	49 × 10^6 km^2	–500 Tg C yr^{-1}		Combination of sparse observations and model results	Schurlock and Hall, 1998[185]
	North America	2000-2005	–124 ± 56 Tg C yr^{-1}	8.2 × 10^6 km^2	–100 [–100 to 0] Tg C yr^{-1} [b]		Atmospheric inversion	Peters et al., 2007[19]
	North America	2000-2006	–101 ± 44 Tg C yr^{-1} (–12 ± 5 g C m^{-2} yr^{-1})	8.2 × 10^6 km^2	–332.5 ± 301.3 Tg C yr^{-1} (–46 ± 42 g C m^{-2} yr^{-1}) [c]		Atmospheric Inversion Models	SOCCR-2, adapted from Hayes et al., 2012[18] #
					–25.2 Tg C yr^{-1} (–3 g C m^{-2} yr^{-1}) [c]		Inventory Analysis	SOCCR-2, adapted from Hayes et al., 2012[18] #
					–130.5 ± 151.8 Tg C yr^{-1} (–18 ± 21 g C m^{-2} yr^{-1}) [c]	7.3 × 10^6 km^2	Process-based model	SOCCR-2, adapted from Hayes et al., 2012[18]

United States (Great Plains)	2000-2008	–18 ± 8 g C m^{-2} yr^{-1}		–24 ± 14 g C m^{-2} yr^{-1}		Flux tower observations up-scaling	Zhang et al., 2011[21] #
United States (Managed grassland)	1990-2009	-106 ± 49 Tg C yr^{-1} (-25 ±12 g C m^{-2} yr^{-1})	4.2 × 10^{6} km^{2}	–11.5 ± 2 Tg C yr^{-1} (–4 ± 1 g C m^{-2} yr^{-1})[d]	< 2.6 × 10^{6} km^{2}	Process-based model + IPCC Tier 2 method	U.S. EPA, 2011 [20] #
United States	2001-2005	–101 ± 46 Tg C yr^{-1} (–24 ± 10 g C m^{-2} yr^{-1})	4.2 × 10^{6} km^{2}	–54.7 Tg C yr^{-1} (–20 g C m^{-2} yr^{-1})	2.7 × 10^{6} km^{2}	Process-based model	The Land Carbon project[22-24]; Liu et al., 2014; Liu et al., 2012; Zhu et al., 2011
Brazil	1980s	85 ± 28 Tg C yr^{-1}	2.3 × 10^{6} km^{2}	72 to 103 Tg C yr^{-1} [e]	1.1 × 10^{6} km^{2}	Combination of independent approaches	Schroeder and Winjum, 1995[37]
New Zealand	between 1960-1992 to 2002-2010	–3 ± 1 g C m^{-2} yr^{-1}		–100 ± 37 to 121 ± 25 g C m^{-2} yr^{-1} [f]		SOC change inventories with repeated soil sampling	Schipper et al., 2010[38]
Russia	around 1990a	–122 ± 56 TgC yr^{-1} (–24 ± 11 g C m^{-2} yr^{-1})	5.3 × 10^{6} km^{2}	–197 Tg C yr^{-1} (–45.6 g C m^{-2} yr^{-1})	4.3 × 10^{6} km^{2}	Combination of independent approaches	Nilsson et al., 2000[39] #
Europe (located at 15 countries)	Since 2002	–62 ± 28 g C m^{-2} yr^{-1}		–76 ± 11 g C m^{-2} yr^{-1} [g]		Flux tower observations + observation of input/output at 39 sites	Soussana et al., 2014[25] #
EU25	2000-2005	–59 ± 27 Tg C yr^{-1} (–59 ± 27 g C m^{-2} yr^{-1})	1.0 × 10^{6} km^{2}	–32 ± 4 Tg C yr^{-1} (–56 ± 7 g C m^{-2} yr^{-1})	0.6 × 10^{6} km^{2}	Combination of independent approaches	Schulze et al., 2009[27] #
Continental Europe	2000-2005	–121 ± 55 Tg C yr^{-1} (–49 ± 22 g C m^{-2} yr^{-1})	2.5 × 10^{6} km^{2}	–85 ± 12 Tg C yr^{-1} (–57 ± 34 g C m^{-2} yr^{-1})	1.5 × 10^{6} km^{2}	Combination of independent approaches	Schulze et al., 2009[27] #
Continental Europe	1990s	–83 ± 38 Tg C yr^{-1} (–35 ± 16 g C m^{-2} yr^{-1})	2.4 × 10^{6} km^{2}	–101 ± 133 Tg C yr^{-1} (–67 ± 88 g C m^{-2} yr^{-1})	1.5 × 10^{6} km^{2}	Combination of independent approaches	Janssens et al., 2003[26] #

England and Wales	1978-2003	–55 ± 25 g C m^{-2} yr^{-1}	A loss of soil carbon [h]		SOC change inventories with repeated soil sampling	Bellamy et al., 2005[28]
Scotland	between 1978-1988 to 2007-2009	–36 ± 16 g C m^{-2} yr^{-1}	No significant change (–17 to 21 g C m^{-2} yr^{-1}) [i]		SOC change inventories with repeated soil sampling	Chapman et al., 2013[29]
UK	1978-2007	–49 ± 22 g C m^{-2} yr^{-1}	No significant change [j]		SOC change inventories with repeated soil sampling	Reynolds et al., 2013[30]
Belgium	1955-2005	–100 ± 46 g C m^{-2} yr^{-1}	–43.8 g C m^{-2} yr^{-1}		SOC change inventories with repeated soil sampling	Goidts and van Wesemael, 2007[31] #
Belgium	1960-2006	–99 ± 45 g C m^{-2} yr^{-1}	–25.4 ± 56.2 g C m^{-2} yr^{-1}		SOC change inventories with regional comparison	Meersmans et al., 2009[33] #
Flanders (Northern Belgium)	1990-2000	–71 ± 33 g C m^{-2} yr^{-1}	150 g C m^{-2} yr^{-1}		SOC change inventories with regional comparison	Mestdagh et al., 2009[35] #
Belgium	1960-2000	–103 ± 47 g C m^{-2} yr^{-1}	–22.5 g C m^{-2} yr^{-1}	3684 km^2	SOC change inventories with regional comparison	Lettens et al., 2005a[32] #
Belgium	1990-2000	–72 ± 33 g C m^{-2} yr^{-1}	90 g C m^{-2} yr^{-1}		SOC change inventories with regional comparison	Lettens et al., 2005b[34] #
Belgium	1984-2000	–85 ±38 g C m^{-2} yr^{-1}	Non-significant increase in SOC [k]		SOC change inventories with regional comparison	Reijneveld et al., 2009[36]
Netherlands	1985-2004	–26 ± 12 g C m^{-2} yr^{-1}	–39 g C m^{-2} yr^{-1}	3350 km^2	SOC change inventories with regional comparison	Hanegraaf et al., 2009[186] #

China	1982-1999	−94 ± 43 Tg C yr^{-1} (−19 ± 9 g C m^{-2} yr^{-1})	4.8×10^6 km^2	−74 ± 14 Tg C yr^{-1} (−14 ± 3 g C m^{-2} yr^{-1})	5.5×10^6 km^2	Combination of independent approaches	Piao et al., 2009[40] #
China (northern)	Between 1980s to 2001-2005	−16 ± 7 g C m^{-2} yr^{-1}		−4 [−23 to 15] g C m^{-2} yr^{-1}	2.0×10^6 km^2	SOC change inventories with regional comparison	Yang et al., 2010[41] #
China (temperate)	2000-2010	−1 ± 0 g C m^{-2} yr^{-1}		−158 ± 25 g C m^{-2} yr^{-1}		Flux tower observations up-scaling	Zhang et al., 2014[45] #
China (temperate)	between 2002-2004 to 2015	−14 ± 6 Tg C yr^{-1} (−16 ± 7 g C m^{-2} yr^{-1})	0.6×10^6 km^2	−22 [−16 to −29] Tg C yr^{-1} (−51 [−35 to −66] g C m^{-2} yr^{-1}) [l]	0.4×10^6 km^2	SOC change inventories with repeated soil sampling	Kou et al., 2018[43] #
China (temperate)	2001-2012	−15 ± 7 g C m^{-2} yr^{-1}		−88 g C m^{-2} yr^{-1}		Remote sensing + empirical model	Dai et al., 2016[42] #
China (temperate)	1951-2007	−14 ± 6 g C m^{-2} yr^{-1}	0.9×10^6 km^2	−11 g C m^{-2} yr^{-1}	0.7×10^6 km^2	Process-based model	Sui et al., 2013[44]
China (southern)	1961-2013	−30 ± 13 g C m^{-2} yr^{-1}	0.7×10^6 km^2	−1.5 g C m^{-2} yr^{-1}	0.3×10^6 km^2	Process-based model	Zhang et al., 2017[47]
China (southern)	2001-2010	−33 ± 15 g C m^{-2} yr^{-1}		−82 to −3 g C m^{-2} yr^{-1}		Process-based model	Sun et al., 2015[46]
China (Qinghai-Tibetan plateau)	Between 1980s and 2001-2004	−18 ± 8 g C m^{-2} yr^{-1}	1.6×10^6 km^2	0.6 [−35.8 to 36.5] gC m^{-2} yr^{-1}	1.1×10^6 km^2	SOC change inventories with regional comparison	Yang et al., 2009[48] #
China (Qinghai-Tibetan plateau)	between 2001-2004 to 2013-2014	−33 ± 15 Tg C yr^{-1} (−21 ± 9 g C m^{-2} yr^{-1})	1.6×10^6 km^2	−32 [−46.7 to −17.4] Tg C yr^{-1} (−28 [−41 to −15] g C m^{-2} yr^{-1}) [m]	1.1×10^6 km^2	SOC change inventories with repeated soil sampling	Ding et al., 2017[49] #
China (Qinghai-Tibetan plateau)	1980-2009	−29 ± 13 Tg C yr^{-1} (−18 ± 8 g C m^{-2} yr^{-1})		−18 Tg C yr^{-1}		Process-based model	Piao et al., 2012[50]

	China (Qinghai-Tibetan plateau)	1961-2010	–14 ± 7 g C m^{-2} yr^{-1}	–10 g C m^{-2} yr^{-1}	Process-based model	Yan et al., 2015[51]
	China (Qinghai-Tibetan plateau)	1990s	–12 ± 6 g C m^{-2} yr^{-1}	–28 g C m^{-2} yr^{-1}	Process-based model	Zhuang et al., 2010[53]
	China (Qinghai-Tibetan plateau)	1981-2012	–18 ± 8 g C m^{-2} yr^{-1}	–4 g C m^{-2} yr^{-1}	Process-based model	Yi et al., 2014[52]
	United States (LETR)	1988-2006	68 ± 31 g C m^{-2} yr^{-1}	A loss of soil carbon (86.5 g C m^{-2} yr^{-1}) [n]	SOC change inventories over a long-term experiment site	Senthilkumar et al., 2009[54] #
	UK (Palace Leas)	1982-2006	5 ± 2 g C m^{-2} yr^{-1}	No significant change (–15 to 78 g C m^{-2} yr^{-1}) [o]	SOC change inventories over a long-term experiment site	Hopkins et al., 2009[55]
	UK (Park Grass)	1959-2002	23 ± 10 g C m^{-2} yr^{-1}	No significant change (–16 to 25 g C m^{-2} yr^{-1}) [o]	SOC change inventories over a long-term experiment site	Hopkins et al., 2009[55]
**CH_4**						
Domestic livestock	World	2003	43 ± 8 Tg CH_4 yr^{-1}	44 Tg CH_4 yr^{-1} [p]	Emission factors and feed statistics	Clark et al., 2005[56]
Domestic livestock	World	2010	49 ± 10 Tg CH_4 yr^{-1}	104 Tg CH_4 yr^{-1} (livestock sector)	Emission factors and livestock statistics	FAOSTAT, 2017[62]
				115 Tg CH_4 yr^{-1} (livestock sector)	Emission factors and livestock statistics	EDGAR v4.3.2 [59]

				103 Tg CH_4 yr^{-1} (livestock sector)	Emission factors and livestock statistics	EPA, 2012[58]
				96 Tg CH_4 yr^{-1} (livestock sector)	Emission factors and livestock statistics	Dangal et al., 2017[60]
				118 ± 16 Tg CH_4 yr^{-1} (livestock sector	Improved emission factors and livestock statistics	Wolf et al., 2017 [57]
				98 ± 11 Tg CH_4 yr^{-1} (livestock sector, enteric fermentation only)	Emissions based on energy content of feed consumption	Chang et al., 2019 [61]
Domestic livestock	World	2000	42 ± 8 Tg CH_4 yr^{-1}	52 Tg CH_4 yr^{-1} (livestock sector)	Emission factors and livestock statistics	Herrero et al., 2013[63]
Wild grazers	World	2000-2009	1.9 ± 0.4 Tg CH_4 yr^{-1}	10 [2 to 15] Tg CH_4 yr^{-1} (grazers + browsers) [q]	Combination of independent approaches	Saunois et al., 2016[68]
Wild grazers	World	2006	1.8 ± 0.4 Tg CH_4 yr^{-1}	13 Tg CH_4 yr^{-1} (grazers + browsers) [r]	Emission factor related to mammal body mass, and wild herbivores dataset	Smith et al., 2016[64]
Wild grazers	World	Contemporary	1.8 ± 0.4 Tg CH_4 yr^{-1}	1.1 to 2.7 CH_4 yr^{-1} (grazers + browsers)	Emission factor related to body mass, and collected world population size	Pérez-Barbería, 2017 [69]
**N_2O**						
Domestic livestock	World	1961	1.5 ± 0.5 Tg N_2O yr^{-1}	1.3 Tg N_2O yr^{-1} [s]	Emission factors and livestock statistics	FAOSTAT, 2017[62]

Domestic livestock	World	2010	2.7 ± 1.0 Tg N_2O yr^{-1}	2.5 Tg N_2O yr^{-1} [s]	Emission factors and livestock statistics	FAOSTAT, 2017[62]
Domestic livestock	World	2000	2.5 ± 0.9 Tg N_2O yr^{-1}	1.8 Tg N_2O yr^{-1}	Emission factors and livestock statistics	Herrero et al., 2013[63]
Domestic livestock	World	1990	2.4 ± 0.8 Tg N_2O yr^{-1}	2.4 Tg N_2O yr^{-1}	Emission factors and livestock statistics	Oenema et al., 1997[70]
Domestic livestock	World	1970-2012	2.4 ± 0.8 Tg N_2O yr^{-1}	2.0 Tg N_2O yr^{-1} [t]	Emission factors and livestock statistics	EDGAR v4.3.2 [59];
Domestic livestock	World	1961-2012	2.2 ± 0.8 Tg N_2O yr^{-1}	2.7 Tg N_2O yr^{-1} [u]	Process-based model	Dangal et al., 2019 [71]

a Schurlock and Hall, 1998: No time span is indicated for this 'best guess' of the global grassland carbon balance. Based on the literature used by Schurlock and Hall (1998), we compare it with the mean value of 1986-1995.

b Peters et al., 2007: The estimate is derived from CarbonTracker data assimilation system for grassland and shrubland; the range indicates the values from a set of sensitivity experiments.

c The values for grasslands in North America were derived from Table 10.1 of SOCCR-2 Chapter 10 (Pendall et al., 2018), which were adapted from Hayes et al. (2012). For estimates using atmospheric inversion models and land-surface models, they were directly derived from the estimates for "other" land in Table 4 of Hayes et al. (2012), given the fact that most of the land "other" than forestland and cropland are grassland, which is also the definition in most land-surface models. For inventory-based estimates, Pendall et al. (2018) used the grassland net ecosystem exchange (NEE_G) for Canada (-3.06 Tg C yr^{-1}) and the United States (-13.16 Tg C yr^{-1}), and the

NEP value for "Others" for Mexico (-9.06 Tg C yr^{-1}; Table S7-S9 of Hayes et al., 2012). In fact, for NEE_G of Canada, Hayes et al. (2012) used the average grassland sink per area from the U.S. data (2.1 gC m^{-2} yr^{-1}); and the NEE_G for the U.S. was derived from the Environmental Protection Agency Greenhouse Gas Inventory (EPA, 2011).

d EPA (2011): U.S. EPA estimated grassland soil carbon stock changes for the period 1990-2009 for "grassland remaining grassland" (sink of -5.2 Tg C yr^{-1} with uncertainty of [-32%, +25%]; Table 7.1 of EPA, 2011) and "land converted to grassland" (sink of -6.3 Tg C yr^{-1} with uncertainty of ±15%; Table 7.1 of EPA, 2011) respectively. The mineral soil carbon stock changes were estimated using the Century biogeochemical model, while the organic soil carbon stock changes were estimated using the Tier 2 method provided in IPCC (2003, 2006), which utilizes U.S.-specific C loss rates (Ogle et al. 2003) rather than default IPCC rates. In total, EPA (2011) estimated a sink of -11 ± 2 Tg C yr^{-1} over "grassland remaining grassland" and "land converted to grassland". It should be noted that the above two grassland categories only include all private-owned grassland but not federal land, thus they are less than the 2.6×10^6 km^2 grassland area shown in Table 7.5 of EPA (2011). This value is not much lower than the one in Table S8 of Hayes et al. (2012). Here, using the grassland area of 2.6×10^6 km^2, we derived the average grassland C sink of -4 ± 1 gC m^{-2} yr^{-1}.

e Schroeder and Winjum, 1995: The estimate is derived by combining grassland area with land use/management induced soil carbon loss derived from literature or by assumption: i.e., 62 to 93 Tg C yr^{-1} carbon loss over 0.151×10^6 km^2 pasture; carbon neutral for 0.74×10^6 km^2 savanna/grasslands; 10 Tg C yr^{-1} carbon loss over 0.179×10^6 km^2 degraded grassland. It did not

consider inter-annual variation from climate impacts. Thus we compare it with the mean value of 1980–1989 including land-use change emissions due to deforestation for pasture.

f Schipper et al., 2010: The estimate is derived from soil re-sampling of 83 profiles under pasture in New Zealand with initial sampling between 1960 and 1992, and re-sampling between 2002 and 2010. On average, soils were resampled 27 years after the first sampling which generally took place in the early 1980s. The values of the soil depth of 0-90 cm were used here. Original literature gives values for four land forms/land uses: 31 ± 27 gC m^{-2} yr^{-1} for flat drystock pastures; 121 ± 25 gC m^{-2} yr^{-1} for flat dairy pastures; -100 ± 37 gC m^{-2} yr^{-1} for north island hill; and 35 ± 8 gC m^{-2} yr^{-1} for south island tussock. We compare it with the mean value of 1980-2006.

g Soussana et al., 2014: The estimate is derived from 213 site-year flux observations + carbon input/output observations from 39 sites located in 15 European countries. We compare the mean value of the 15 countries for the period 2002–2012.

h Bellamy et al., 2005: No exact value is available from literature. From Fig. 2 of Bellamy et al., 2005, the values for grassland SOC change for England and Wales between 1978–2003 are just below 0 g kg^{-1} yr^{-1} for rotational grass and permanent grass, ca. -0.5 g kg^{-1} yr^{-1} for rough grazing, and just greater than -2 g kg^{-1} yr^{-1} for upland grass. All values show losses of soil organic carbon over grasslands of England and Wales.

i Chapman et al., 2013: The measurement shows a decrease in mean total carbon stock of 450gC m^{-2} over improved grassland, and an increase of 540 gC m^{-2} over semi-natural grassland between the two inventories (the first one in 1978–1988, the second one in 2007–2009). But both changes are not significant.

j Reynolds et al., 2013: The measurements were presented as topsoil carbon concentration. No significant overall changes in topsoil carbon concentration were observed between 1978 and 2007.

k Reijneveld et al., 2009: A non-significant increase in SOC of 0.1 g kg^{-1} of soil over grassland was observed.

l Kou et al., 2018: The estimate is derived from soil re-sampling of 76 profiles (0.5 m depth) under Inner Mongolian grasslands with initial sampling between 2002 and 2004, and re-sampling in 2015. Values in brackets indicate 95% confidence interval. We compare it with the mean value of 2003–2012.

m Ding et al., 2017: The estimate is derived from soil re-sampling of 103 profiles (30 cm depth) under Tibetan grasslands with initial sampling between 2001 and 2004, and re-sampling between 2013 and 2014. Values in brackets indicate 95% confidence interval. We compare it with the mean value of 2002–2012.

n Senthilkumar et al., 2009: A soil carbon loss of 7.7 g kg of soil was measured. The average bulk density measured in 2007 was 1.12 g cm^{-3}; this value was assumed to be applicable to the 1986 data as well. As a result, a carbon loss of 1730 g C m^{-2} was estimated between 1987 and 2006. The lack of bulk density measurements from the 1980s is a drawback for the C stock calculations of this study that reduces accuracy in the estimates of C stocks; however, it does not affect C concentration results. For estimates with SOC change inventories over long-term experimental sites, we compare them with model output of the $0.5^{o} \times 0.5^{o}$ grid cell in which the experiments were located or of the surrounding grid cell (using the grid cell with the mean annual temperature and annual total precipitation closest to the values reported by literature).

o Hopkins et al., 2009: The plots in these two experiment sites did not show consistent changes in SOC. For the PL site, four plots showed no significant changes in SOC (SOC increase can be as high as 3.42 t C ha^{-1} using plot 6 data from Table 2 of Hopkins et al., 2009), while the other two plots showed significant losses of carbon of 18 and 15 t C ha^{-1}. For the PG site, SOC change ranges from carbon losses of 10.5 t C ha^{-1} to carbon gain of 6.6 t C ha^{-1}. Authors found no significant SOC differences between the two inventories for these two sites. We compare them with model output of the $0.5^{o} \times 0.5^{o}$ grid cell in which the experiments were located or of the surrounding grid cell (using the grid cell with the mean annual temperature and annual total precipitation closest to the values reported by literature).

p Clark et al., 2005: The estimate is calculated using grassland-derived feed and emission factors.

q Saunois et al., 2016: This estimate accounted for all wild animals including grazers and browsers with large uncertainty (Bouwman et al., 1997). Original data from Leng (1993; 2-6 Tg CH_4 yr^{-1}) and Houweling et al. (2000; 15 Tg CH_4 yr^{-1}).

r Smith et al., 2016: This estimate accounted for all wild animals including grazers and browsers; the contemporary wild animal CH_4 emissions are derived from emissions of 1800 and consider the reduction by regional percentage urbanization of natural habitats.

s FAOSTAT, 2017: This estimate is derived from manure left on pasture, but does not account for the N_2O emissions due to mineral fertilizer application over grassland in some regions (e.g., Bouwman et al., 2002).

t EDGAR v4.3.2; This estimate includes N_2O emissions from “Manure in pasture/range/paddock” and “Manure management”.

u Dangal et al., 2019: This N_2O estimate is derived from DLEM model simulation with representation of soil N dynamics associated with N_2O emissions

Supplementary Table 3. The absolute and relative contributions of grassland to the various components of global radiative forcing in 2012.

	radiative forcing ($mW\ m^{-2}$)	Relative contribution (%)
CO_2 soil sink	-194 ± 99	-11 ± 5%
Managed	-41 ± 22	
Sparsely grazed	-152 ± 78	
CO_2 E_{LUC}	108 ± 35	7 ± 2%
Deforestation to pasture	81 ± 26	
Conversion of grassland to cropland	27 ± 9	
CH_4 (including O_3 and H_2O)	70 ± 15	14 ± 3%
Managed	95 ± 20	
Sparsely grazed	-25 ± 5	
N_2O (including O_3)	30 ± 10	17 ± 6%
Managed	52 ± 18	
Sparsely grazed	-22 ± 9	
Net GHGs	15 ± 105	1 ± 4%
Albedo	-3 ± 8	1 ± 7%
Grassland Total	12 ± 105	1 ± 4%
Managed	204 ±48	
Sparsely grazed	-193 ± 80	

Supplementary Table 4. Comparison of the historic land-cover change maps used in ref [133] and in this study.

	Chang et al., 2016 ref [133]	This study
Reference map	GLC2000 for year 2000[182,187]	ESA CCI for year 2010[137]
Methodology used to produce PFT map	Poulter et al., 2011 (ref[138])	Poulter et al., 2011; 2015 (ref[138,139])
Historical land-use forcing data set	LUH1[140]	LUH2v2 (http://luh.umd.edu/data.shtml)
Constraint of Cropland area	HYDE3.1 [188]	HYDE3.2[141]
Method used to define the land-cover transitions between natural vegetation and agriculture	BM3 method[96]	BM3 method[96]

Supplementary Table 5. Uncertainty estimates for the grassland carbon sink and land-use change emission related to grassland in this study. A negative value indicates a carbon sink, and a positive value indicates a carbon source. Relative uncertainties in bold are used as the uncertainty of grassland carbon sink (46%) and land-use change emission related to grassland (31%) in this study.

	Standard error of the mean distance (g C m^{-2} yr^{-1})	Mean of the simulated carbon budgets (g C m^{-2} yr^{-1})	Relative uncertainty
Grassland carbon fluxes	16	-35	46%
	Standard deviation from 16 TRENDYv6 models (Gt C yr^{-1})	Net land carbon fluxes from 16 TRENDYv6 models (Gt C yr^{-1})	Relative uncertainty
Net land carbon fluxes (average of 1960-2009)	-0.61	-1.44	42%
	Standard deviation from 12 TRENDYv6 models (Gt C yr^{-1})	Multi-model ensemble mean of 12 TRENDYv6 models (Gt C yr^{-1})	Relative uncertainty
Land-use change emissions	0.39	1.22	31%

Supplementary Table 6. Comparison of overgrazed grassland and portion of overgrazed land within each severity class in the 1980s from model simulation and from the Global Assessment of Soil Degradation **(GLASOD) Database.** The data from GLASOD are adapted from ref[78], which originated from the Global Assessment of Soil Degradation[76].

	Overgrazed grassland from model simulation (million ha)				Overgrazed grassland from GLASOD Database (million ha)			
Continent	Total	Light	Moderate	Strong	Total	Light	Moderate	Strong and Extreme
Africa	106.6	78.5%	14.3%	7.3%	87.7	34.6%	35.2%	30.2%
Australia/Pacific	28.8	68.6%	14.5%	16.9%	49.1	97.5%	2.3%	0.2%
Eurasia	56.5	68.0%	24.9%	7.1%	85.6	54.8%	41.8%	3.4%
North America	29.5	76.9%	11.5%	11.5%	14	14.5%	73.4%	12.1%
South America	41.1	71.6%	19.7%	8.7%	26.2	35.8%	57.6%	6.6%
Global	262.5	73.9%	17.1%	9.0%	262.5	52.0%	35.5%	12.5%

Supplementary Table 7. The simulation protocol and the drivers used for the factorial simulations. The contribution of climate change, rising CO_2, atmospheric nitrogen deposition, and grassland management (including manure and fertilizer application) together with the extirpation of wild grazing is calculated as the differences between the net CO_2 fluxes between $E_{reference}$ and $E_{climate}$, E_{co2}, $E_{deposition}$, and $E_{management}$ respectively. The contribution of land-use change is the land-use change emissions (CO_2 E_{LUC}) related to grassland as described in the methods section of the main text. The CO_2 E_{LUC} of deforestation to pasture is calculated as the difference in the net CO_2 fluxes between $E_{ELUC\_reference}$ and E_{ELUC_FtoG}, and the CO_2 E_{LUC} of conversion of grassland to cropland is calculated as the difference in the net CO_2 fluxes between $E_{ELUC\_reference}$, and E_{ELUC_GtoC}.

	Simulation $E_{reference}$	Simulation $E_{climate}$	Simulation E_{co2}	Simulation $E_{deposition}$	Simulation $E_{management}$	Simulation $E_{ELUC\_reference}$	Simulation E_{ELUC_FtoG}	Simulation E_{ELUC_FtoC}
Simulation period	1861-2012	1861-2012	1861-2012	1861-2012	1861-2012	1861-2012	1861-2012	1861-2012
Input data								
Climate forcing	1901–1910 cycled for 1861-1900, and historical climate for 1901–2012	1901–1910 cycled	Same as E3 to E4	Same as $E_{reference}$	Same as $E_{reference}$	Same as $E_{reference}$	Same as $E_{reference}$	Same as $E_{reference}$
Atmospheric CO_2 concentration	1861-2012	1861-2012	1860	1861-2012	1861-2012	1861-2012	1861-2012	1861-2012
Atmospheric-nitrogen deposition	1861-2012	1861-2012	1861-2012	1860	1861-2012	1861-2012	1861-2012	1861-2012
Historic Land-cover change	1861-2012	1861-2012	1861-2012	1861-2012	1861-2012	Time invariant land cover of 1860 for all biomes	Time invariant cropland of 1860, and historical land use transitions from forest to grassland	Time invariant forest of 1860, and historical land use transitions from grassland to cropland

Grassland management	1861-2012	1861-2012	1861-2012	1861-2012	1860	Not applied	Not applied	Not applied
Wild-grazers population density	1861-2012	1861-2012	1861-2012	1861-2012	1860	Not applied	Not applied	Not applied

Supplementary References

1 Le Quéré, C. *et al.* Global Carbon Budget 2017. *Earth System Science Data* **10**, 405-448 (2018).

2 Pan, Y. *et al.* A large and persistent carbon sink in the world's forests. *Science* **333**, 988-993 (2011).

3 Carlson, K. M. *et al.* Greenhouse gas emissions intensity of global croplands. *Nature Climate Change* **7**, 63-+, doi:10.1038/Nclimate3158 (2017).

4 Smith, W. K. *et al.* Large divergence of satellite and Earth system model estimates of global terrestrial CO2 fertilization. *Nature Climate Change* **6**, 306-310 (2016).

5 Zhao, M. & Running, S. W. Drought-induced reduction in global terrestrial net primary production from 2000 through 2009. *Science* **329**, 940-943 (2010).

6 Zhao, M. S., Heinsch, F. A., Nemani, R. R. & Running, S. W. Improvements of the MODIS terrestrial gross and net primary production global data set. *Remote Sensing of Environment* **95**, 164-176, doi:10.1016/j.rse.2004.12.011 (2005).

7 Chang, J. *et al.* The greenhouse gas balance of European grasslands. *Global Change Biology* **21**, 3748-3761 (2015).

8 Chang, J. F. *et al.* Effect of climate change, CO2 trends, nitrogen addition, and land-cover and management intensity changes on the carbon balance of European grasslands. *Global Change Biology* **22**, 338-350, doi:10.1111/gcb.13050 (2016).

9 Tucker, C. J. *et al.* An extended AVHRR 8-km NDVI dataset compatible with MODIS and SPOT vegetation NDVI data. *International Journal of Remote Sensing* **26**, 4485-4498, doi:10.1080/01431160500168686 (2005).

10 Piao, S., Mohammat, A., Fang, J., Cai, Q. & Feng, J. NDVI-based increase in growth of temperate grasslands and its responses to climate changes in China. *Global Environmental Change* **16**, 340-348 (2006).

11 Beck, H. E. *et al.* Global evaluation of four AVHRR–NDVI data sets: Intercomparison and assessment against Landsat imagery. *Remote Sensing of Environment* **115**, 2547-2563 (2011).

12 Carvalhais, N. *et al.* Global covariation of carbon turnover times with climate in terrestrial ecosystems. *Nature* **514**, 213 (2014).

13 Tifafi, M., Guenet, B. & Hatté, C. Large differences in global and regional total soil carbon stock estimates based on SoilGrids, HWSD, and NCSCD: Intercomparison and evaluation based on field data from USA, England, Wales, and France. *Global Biogeochemical Cycles* **32**, 42-56 (2018).

14 Hengl, T. *et al.* SoilGrids250m: Global gridded soil information based on machine learning. *PLoS one* **12**, e0169748 (2017).

15 Hengl, T. *et al.* SoilGrids1km—global soil information based on automated mapping. *PloS one* **9**, e105992 (2014).

16 Batjes, N. Harmonized soil property values for broad-scale modelling (WISE30sec) with estimates of global soil carbon stocks. *Geoderma* **269**, 61-68 (2016).

17 Saxton, K., Rawls, W. J., Romberger, J. & Papendick, R. Estimating generalized soil-water characteristics from texture 1. *Soil Science Society of America Journal* **50**, 1031-1036 (1986).

18 Hayes, D. J. *et al.* Reconciling estimates of the contemporary North American carbon balance among terrestrial biosphere models, atmospheric inversions, and a new approach for estimating net ecosystem exchange from inventory‐based data. *Global Change Biology* **18**, 1282-1299 (2012).

19 Peters, W. *et al.* An atmospheric perspective on North American carbon dioxide exchange: CarbonTracker. *Proceedings of the National Academy of Sciences* **104**, 18925-18930 (2007).

20 EPA. Inventory of US greenhouse gas emissions and sinks: 1990-2009. *US Environmental Protection Agency, Washington, DC* (2011).
21 Zhang, L. *et al.* Upscaling carbon fluxes over the Great Plains grasslands: Sinks and sources. *Journal of Geophysical Research: Biogeosciences* **116,** doi:10.1029/2011JG001832(2011).
22 Liu, S. *et al.* Baseline and projected future carbon storage, carbon sequestration, and greenhouse-gas fluxes in terrestrial ecosystems of the eastern United States. *Baseline and Projected Future Carbon Storage and Greenhouse-Gas Fluxes in Ecosystems of the Eastern United States*, 115-156 (2014).
23 Liu, S. *et al.* Baseline carbon storage, carbon sequestration, and greenhouse-gas fluxes in terrestrial ecosystems of the Western United States. *Baseline and projected future carbon storage and greenhouse-gas fluxes in ecosystems of the Western United States. Professional Paper*, 45-63 (2012).
24 Zhu, Z. *et al.* Baseline and projected future carbon storage and greenhouse-gas fluxes in the Great Plains Region of the United States: U.S. Geological Survey Professional Paper 1787, 28 p. (Also available at http://pubs.usgs.gov/pp/1787/). (2011).
25 Soussana, J., Klumpp, K. & Ehrhardt, F. The role of grassland in mitigating climate change. *The Future of European Grasslands* **75**, 75-90 (2014).
26 Janssens, I. A. *et al.* Europe's terrestrial biosphere absorbs 7 to 12% of European anthropogenic CO2 emissions. *Science* **300**, 1538-1542, doi:10.1126/science.1083592 (2003).
27 Schulze, E. D. *et al.* Importance of methane and nitrous oxide for Europe's terrestrial greenhouse-gas balance. *Nature Geoscience* **2**, 842-850, doi:10.1038/ngeo686 (2009).
28 Bellamy, P. H., Loveland, P. J., Bradley, R. I., Lark, R. M. & Kirk, G. J. D. Carbon losses from all soils across England and Wales 1978-2003. *Nature* **437**, 245-248, doi:10.1038/nature04038 (2005).
29 Chapman, S. *et al.* Comparison of soil carbon stocks in S cottish soils between 1978 and 2009. *European Journal of Soil Science* **64**, 455-465 (2013).
30 Reynolds, B. *et al.* Countryside Survey: National "Soil Change" 1978–2007 for Topsoils in Great Britain—acidity, carbon, and total nitrogen status. *Vadose Zone Journal* **12** (2013).
31 Goidts, E. & van Wesemael, B. Regional assessment of soil organic carbon changes under agriculture in Southern Belgium (1955–2005). *Geoderma* **141**, 341-354 (2007).
32 Lettens, S., Van Orshoven, J., Van Wesemael, B., Muys, B. & Perrin, D. Soil organic carbon changes in landscape units of Belgium between 1960 and 2000 with reference to 1990. *Global Change Biology* **11**, 2128-2140 (2005).
33 Meersmans, J. *et al.* Changes in organic carbon distribution with depth in agricultural soils in northern Belgium, 1960–2006. *Global Change Biology* **15**, 2739-2750 (2009).
34 Lettens, S., Van Orshoven, J., van Wesemael, B., De Vos, B. & Muys, B. Stocks and fluxes of soil organic carbon for landscape units in Belgium derived from heterogeneous data sets for 1990 and 2000. *Geoderma* **127**, 11-23 (2005).
35 Mestdagh, I. *et al.* Soil organic carbon–stock changes in Flemish grassland soils from 1990 to 2000. *Journal of Plant Nutrition and Soil Science* **172**, 24-31 (2009).
36 Reijneveld, A., van Wensem, J. & Oenema, O. Soil organic carbon contents of agricultural land in the Netherlands between 1984 and 2004. *Geoderma* **152**, 231-238 (2009).
37 Schroeder, P. E. & Winjum, J. K. Assessing Brazil's carbon budget: II. Biotic fluxes and net carbon balance. *Forest Ecology and Management* **75**, 87-99 (1995).
38 Schipper, L. A. *et al.* Gains and losses in C and N stocks of New Zealand pasture soils depend on land use. *Agriculture, Ecosystems & Environment* **139**, 611-617 (2010).

39 Nilsson, S. *et al.* Full carbon account for Russia. *IIASA Interim Report IR-00-021* (2000).
40 Piao, S. *et al.* The carbon balance of terrestrial ecosystems in China. *Nature* **458**, 1009 (2009).
41 Yang, Y. *et al.* Soil carbon stock and its changes in northern China's grasslands from 1980s to 2000s. *Global Change Biology* **16**, 3036-3047 (2010).
42 Dai, E., Huang, Y., Wu, Z. & Zhao, D. Analysis of spatio-temporal features of a carbon source/sink and its relationship to climatic factors in the Inner Mongolia grassland ecosystem. *Journal of Geographical Sciences* **26**, 297-312 (2016).
43 Kou, D. *et al.* Dryland soils in northern China sequester carbon during the early 2000s warming hiatus period. *Functional Ecology* **32**, 1620-1630 (2018).
44 Sui, X. & Zhou, G. Carbon dynamics of temperate grassland ecosystems in China from 1951 to 2007: an analysis with a process-based biogeochemistry model. *Environmental Earth Sciences* **68**, 521-533 (2013).
45 Zhang, L. *et al.* Net ecosystem productivity of temperate grasslands in northern China: An upscaling study. *Agricultural and Forest Meteorology* **184**, 71-81 (2014).
46 Sun, Z., Chen, Y., Ju, W., Zhou, W. & Li, J. Productivity of different types of grassland plots and their responses to climate change in the southern China. *Resources and Environment in the Yangtze Basin* **24**, 609-616 (2015).
47 Zhang, L., Zhou, G., Ji, Y. & Bai, Y. Grassland Carbon Budget and Its Driving Factors of the Subtropical and Tropical Monsoon Region in China During 1961 to 2013. *Sci Rep-Uk* **7**, 14717 (2017).
48 Yang, Y. *et al.* Changes in topsoil carbon stock in the Tibetan grasslands between the 1980s and 2004. *Global Change Biology* **15**, 2723-2729 (2009).
49 Ding, J. *et al.* Decadal soil carbon accumulation across Tibetan permafrost regions. *Nature Geoscience* **10**, 420 (2017).
50 Piao, S. *et al.* Impacts of climate and CO2 changes on the vegetation growth and carbon balance of Qinghai–Tibetan grasslands over the past five decades. *Global and Planetary Change* **98**, 73-80 (2012).
51 Yan, L., Zhou, G., Wang, Y., Hu, T. & Sui, X. The spatial and temporal dynamics of carbon budget in the alpine grasslands on the Qinghai-Tibetan Plateau using the Terrestrial Ecosystem Model. *Journal of Cleaner Production* **107**, 195-201 (2015).
52 Yi, S., Wang, X., Qin, Y., Xiang, B. & Ding, Y. Responses of alpine grassland on Qinghai–Tibetan plateau to climate warming and permafrost degradation: a modeling perspective. *Environmental Research Letters* **9**, 074014 (2014).
53 Zhuang, Q. *et al.* Carbon dynamics of terrestrial ecosystems on the Tibetan Plateau during the 20th century: an analysis with a process‐based biogeochemical model. *Global Ecology and Biogeography* **19**, 649-662 (2010).
54 Senthilkumar, S., Basso, B., Kravchenko, A. & Robertson, G. Contemporary evidence of soil carbon loss in the US corn belt. *Soil Science Society of America Journal* **73**, 2078-2086 (2009).
55 Hopkins, D. *et al.* Soil organic carbon contents in long‐term experimental grassland plots in the UK (Palace Leas and Park Grass) have not changed consistently in recent decades. *Global Change Biology* **15**, 1739-1754 (2009).
56 Clark, H., Pinares-Patino, C. & De Klein, C. 279-293 (Wageningen Academic Publishers, Wageningen, The Netherlands, 2005).
57 Wolf, J., Asrar, G. R. & West, T. O. Revised methane emissions factors and spatially distributed annual carbon fluxes for global livestock. *Carbon Balance and Management* **12**, 16 (2017).

58 EPA. Global Anthropogenic Non-CO2 Greenhouse Gas Emissions: 1990-2030. . *United States Environment Protection Agency, Washington DC.* (2012).

59 EDGARv4.3.2. http://edgar.jrc.ec.europa.eu/, last access: September 2018. (2018).

60 Dangal, S. R. *et al.* Methane emission from global livestock sector during 1890–2014: Magnitude, trends and spatiotemporal patterns. *Global Change Biology* **23**, 4147-4161 (2017).

61 Chang, J. *et al.* Revisiting enteric methane emissions from domestic ruminants and their δ13CCH4 source signature. *Nature Communications* **10**, 3420, doi:10.1038/s41467-019-11066-3 (2019).

62 FAOSTAT. Online Statistical Service (Food and Agriculture Organization (FAO), 2017); http://faostat3.fao.org. (2017).

63 Herrero, M. *et al.* Biomass use, production, feed efficiencies, and greenhouse gas emissions from global livestock systems. *Proceedings of the National Academy of Sciences of the United States of America* **110**, 20888-20893, doi:10.1073/pnas.1308149110 (2013).

64 Smith, F. A. *et al.* Exploring the influence of ancient and historic megaherbivore extirpations on the global methane budget. *Proceedings of the National Academy of Sciences* **113**, 874-879 (2016).

65 Owen‐Smith, N. Contrasts in the large herbivore faunas of the southern continents in the late Pleistocene and the ecological implications for human origins. *Journal of Biogeography* **40**, 1215-1224 (2013).

66 Ellis, E. C., Klein Goldewijk, K., Siebert, S., Lightman, D. & Ramankutty, N. Anthropogenic transformation of the biomes, 1700 to 2000. *Global Ecology and Biogeography* **19**, 589-606 (2010).

67 Ellis, E. C. & Ramankutty, N. Putting people in the map: anthropogenic biomes of the world. *Frontiers in Ecology and the Environment* **6**, 439-447 (2008).

68 Saunois, M. *et al.* The global methane budget 2000–2012. *Earth System Science Data (Online)* **8** (2016).

69 Pérez-Barbería, F. Scaling methane emissions in ruminants and global estimates in wild populations. *Science of the Total Environment* **579**, 1572-1580 (2017).

70 Oenema, O., Velthof, G., Yamulki, S. & Jarvis, S. Nitrous oxide emissions from grazed grassland. *Soil Use and Management* **13**, 288-295 (1997).

71 Dangal, S. R. S. *et al.* Global nitrous oxide emissions from pasturelands and rangelands: Magnitude, spatio-temporal patterns and attribution. *Global Biogeochemical Cycles* **33**, doi:10.1029/2018GB006091 (2019).

72 Guimberteau, M. *et al.* ORCHIDEE-MICT (v8.4.1), a land surface model for the high latitudes: model description and validation. *Geoscientific Model Development* **11**, 121-163, doi:10.5194/gmd-11-121-2018 (2018).

73 Koven, C. *et al.* On the formation of high-latitude soil carbon stocks: Effects of cryoturbation and insulation by organic matter in a land surface model. *Geophysical Research Letters* **36**, doi:10.1029/2009gl040150 (2009).

74 Zhu, D. *et al.* Simulating soil organic carbon in yedoma deposits during the Last Glacial Maximum in a land surface model. *Geophysical Research Letters* **43**, 5133-5142, doi:10.1002/2016gl068874 (2016).

75 Balesdent, J. *et al.* Atmosphere–soil carbon transfer as a function of soil depth. *Nature* **559**, 599-602, doi:10.1038/s41586-018-0328-3 (2018).

76 Oldeman, L., Hakkeling, R., Sombroek, W. & Batjes, N. Global assessment of human-induced soil degradation (GLASOD). *World map of the status of human-induced soil degradation. Wageningen, Netherlands: Winand Staring Centre–ISSSFAO–ITC* (1991).

77 Abdalla, M. *et al.* Critical review of the impacts of grazing intensity on soil organic carbon storage and other soil quality indicators in extensively managed grasslands. *Agriculture, Ecosystems & Environment* **253**, 62-81 (2018).
78 Conant, R. T. & Paustian, K. Potential soil carbon sequestration in overgrazed grassland ecosystems. *Global Biogeochemical Cycles* **16**, doi:10.1029/2001gb001661 (2002).
79 Dlamini, P., Chivenge, P. & Chaplot, V. Overgrazing decreases soil organic carbon stocks the most under dry climates and low soil pH: A meta-analysis shows. *Agriculture, Ecosystems & Environment* **221**, 258-269, doi:10.1016/j.agee.2016.01.026 (2016).
80 Ibrahim, Y., Balzter, H., Kaduk, J. & Tucker, C. Land degradation assessment using residual trend analysis of GIMMS NDVI3g, soil moisture and rainfall in Sub-Saharan West Africa from 1982 to 2012. *Remote Sens-Basel* **7**, 5471-5494 (2015).
81 Wessels, K. J., Prince, S. D., Frost, P. E. & van Zyl, D. Assessing the effects of human-induced land degradation in the former homelands of northern South Africa with a 1 km AVHRR NDVI time-series. *Remote Sensing of Environment* **91**, 47-67, doi:10.1016/j.rse.2004.02.005 (2004).
82 Bai, Z. G., Dent, D. L., Olsson, L. & Schaepman, M. E. Proxy global assessment of land degradation. *Soil Use and Management* **24**, 223-234, doi:10.1111/j.1475-2743.2008.00169.x (2008).
83 Kwon, H.-Y. *et al.* in *Economics of Land Degradation and Improvement – A Global Assessment for Sustainable Development* (eds Ephraim Nkonya, Alisher Mirzabaev, & Joachim von Braun) 197-214 (Springer International Publishing, 2016).
84 Le, Q. B., Nkonya, E. & Mirzabaev, A. in *Economics of Land Degradation and Improvement – A Global Assessment for Sustainable Development* (eds Ephraim Nkonya, Alisher Mirzabaev, & Joachim von Braun) 55-84 (Springer International Publishing, 2016).
85 Olson, D. M. *et al.* Terrestrial Ecoregions of the World: A New Map of Life on Earth: A new global map of terrestrial ecoregions provides an innovative tool for conserving biodiversity. *BioScience* **51**, 933-938, doi:10.1641/0006-3568(2001)051[0933:Teotwa]2.0.Co;2 (2001).
86 Robinson, T. P. *et al.* Mapping the global distribution of livestock. *PloS one* **9**, e96084 (2014).
87 Chang, J. *et al.* Modeled changes in potential grassland productivity and in grass-fed ruminant livestock density in Europe over 1961–2010. *PLoS One* **10**, e0127554, doi:10.1371/journal.pone.0127554 (2015).
88 Grieser, J., Gommes, R. & Bernardi, M. The Miami Model of climatic net primary production of biomass. *FAO of the UN, Italy* (2006).
89 Lieth, H. in *Primary Productivity of the Biosphere* (eds Helmut Lieth & Robert H. Whittaker) 237-263 (Springer Berlin Heidelberg, 1975).
90 Smith, P. *et al.* Greenhouse gas mitigation in agriculture. *Philosophical Transactions of the Royal Society B: Biological Sciences* **363**, 789-813, doi:10.1098/rstb.2007.2184 (2008).
91 Schuman, G. E., Reeder, J. D., Manley, J. T., Hart, R. H. & Manley, W. A. Impact of grazing management on the carbon and nitrogen balance of a mixed-grass rangeland. *Ecological Applications* **9**, 65-71, doi:10.2307/2641168 (1999).
92 Holechek, J. L. A brief history of range management in the United States. *Rangelands Archives* **3**, 16-18 (1981).
93 Naipal, V. *et al.* Global soil organic carbon removal by water erosion under climate change and land use change during AD 1850–2005. *Biogeosciences* **15**, 4459-4480, doi:10.5194/bg-15-4459-2018 (2018).

94 Naipal, V., Reick, C., Pongratz, J. & Van Oost, K. Improving the global applicability of the RUSLE model – adjustment of the topographical and rainfall erosivity factors. *Geoscientific Model Development* **8**, 2893-2913, doi:10.5194/gmd-8-2893-2015 (2015).
95 Naipal, V., Reick, C., Van Oost, K., Hoffmann, T. & Pongratz, J. Modeling long-term, large-scale sediment storage using a simple sediment budget approach. *Earth Surface Dynamics* **4**, 407-423, doi:10.5194/esurf-4-407-2016 (2016).
96 Peng, S. *et al.* Sensitivity of land use change emission estimates to historical land use and land cover mapping. *Global Biogeochemical Cycles* **31**, 626-643 (2017).
97 Panagos, P. *et al.* Global rainfall erosivity assessment based on high-temporal resolution rainfall records. *Sci Rep-Uk* **7**, 4175, doi:10.1038/s41598-017-04282-8 (2017).
98 Chappell, A. *et al.* Minimising soil organic carbon erosion by wind is critical for land degradation neutrality. *Environmental Science & Policy* **93**, 43-52, doi:10.1016/j.envsci.2018.12.020 (2019).
99 Hoffmann, T. *et al.* Short Communication: Humans and the missing C-sink: erosion and burial of soil carbon through time. *Earth Surface Dynamics* **1**, 45-52, doi:10.5194/esurf-1-45-2013 (2013).
100 Van Oost, K. *et al.* The Impact of Agricultural Soil Erosion on the Global Carbon Cycle. *Science* **318**, 626-629, doi:10.1126/science.1145724 (2007).
101 Wang, Z. *et al.* Human-induced erosion has offset one-third of carbon emissions from land cover change. *Nature Climate Change* **7**, 345, doi:10.1038/nclimate3263 (2017).
102 Yue, C. *et al.* Modelling the role of fires in the terrestrial carbon balance by incorporating SPITFIRE into the global vegetation model ORCHIDEE – Part 1: simulating historical global burned area and fire regimes. *Geoscientific Model Development* **7**, 2747-2767, doi:10.5194/gmd-7-2747-2014 (2014).
103 Yue, C., Ciais, P., Cadule, P., Thonicke, K. & van Leeuwen, T. Modelling the role of fires in the terrestrial carbon balance by incorporating SPITFIRE into the global vegetation model ORCHIDEE-Part 2: Carbon emissions and the role of fires in the global carbon balance. *Geoscientific Model Development* **8**, 1321-1338 (2015).
104 Rabin, S. S. *et al.* The Fire Modeling Intercomparison Project (FireMIP), phase 1: experimental and analytical protocols with detailed model descriptions. *Geoscientific Model Development* **10**, 1175-1197, doi:10.5194/gmd-10-1175-2017 (2017).
105 Teckentrup, L. *et al.* Response of simulated burned area to historical changes in environmental and anthropogenic factors: a comparison of seven fire models. *Biogeosciences* **16**, 3883-3910, doi:10.5194/bg-16-3883-2019 (2019).
106 Andela, N. & van der Werf, G. R. Recent trends in African fires driven by cropland expansion and El Niño to La Niña transition. *Nature Climate Change* **4**, 791-795, doi:10.1038/nclimate2313 (2014).
107 Akagi, S. *et al.* Emission factors for open and domestic biomass burning for use in atmospheric models. *Atmospheric Chemistry and Physics* **11**, 4039-4072 (2011).
108 Van Der Werf, G. R. *et al.* Global fire emissions estimates during 1997–2016. *Earth System Science Data* **9**, 697 (2017).
109 Livesley, S. J. *et al.* Seasonal variation and fire effects on CH4, N2O and CO2 exchange in savanna soils of northern Australia. *Agricultural and Forest Meteorology* **151**, 1440-1452, doi:10.1016/j.agrformet.2011.02.001 (2011).
110 Change, I. P. o. C. IPCC special report on climate change, desertification, land degradation, sustainable land management, food security, and greenhouse gas fluxes in terrestrial ecosystems. Summary for policymakers. (2019).
111 Rötter, R. & van de Geijn, S. C. Climate Change Effects on Plant Growth, Crop Yield and Livestock. *Climatic Change* **43**, 651-681, doi:10.1023/A:1005541132734 (1999).

112 Soussana, J. F. & Luscher, A. Temperate grasslands and global atmospheric change: a review. *Grass and Forage Science* **62**, 127-134, doi:DOI 10.1111/j.1365-2494.2007.00577.x (2007).
113 Brookshire, E. N. J. & Weaver, T. Long-term decline in grassland productivity driven by increasing dryness. *Nature Communications* **6**, 7148, doi:10.1038/ncomms8148 (2015).
114 Dukes, J. *et al.* Responses of Grassland Production to Single and Multiple Global Environmental Changes. *PLoS biology* **3**, e319, doi:10.1371/journal.pbio.0030319 (2005).
115 Jones, M. B. & Donnelly, A. Carbon sequestration in temperate grassland ecosystems and the influence of management, climate and elevated CO(2). *New Phytologist* **164**, 423-439, doi:10.1111/j.1469-8137.2004.01201.x (2004).
116 Lee, M., Manning, P., Rist, J., Power, S. A. & Marsh, C. A global comparison of grassland biomass responses to CO2 and nitrogen enrichment. *Philosophical Transactions of the Royal Society B: Biological Sciences* **365**, 2047-2056, doi:10.1098/rstb.2010.0028 (2010).
117 Ehlers, I. *et al.* Detecting long-term metabolic shifts using isotopomers: CO₂-driven suppression of photorespiration in C₃ plants over the 20th century. *Proceedings of the National Academy of Sciences* **112**, 15585-15590, doi:10.1073/pnas.1504493112 (2015).
118 Campbell, J. E. *et al.* Large historical growth in global terrestrial gross primary production. *Nature* **544**, 84-87, doi:10.1038/nature22030 (2017).
119 Gasser, T. *et al.* The compact Earth system model OSCAR v2.2: description and first results. *Geoscientific Model Development* **10**, 271-319, doi:10.5194/gmd-10-271-2017 (2017).
120 Myhre, G. *et al.* in *Climate Change 2013: The Physical Science Basis. Contribution of Working Group I to the Fifth Assessment Report of the Intergovernmental Panel on Climate Change* (eds T. F. Stocker *et al.*) 659-740 (Cambridge University Press, 2013).
121 Archer, D. *et al.* Atmospheric lifetime of fossil fuel carbon dioxide. *Annual Review of Earth and Planetary Sciences* **37**, 117-134 (2009).
122 Joos, F. *et al.* Carbon dioxide and climate impulse response functions for the computation of greenhouse gas metrics: a multi-model analysis. *Atmospheric Chemistry and Physics* **13**, 2793-2825 (2013).
123 Ciais, P. *et al.* Europe-wide reduction in primary productivity caused by the heat and drought in 2003. *Nature* **437**, 529-533, doi:10.1038/nature03972 (2005).
124 Krinner, G. *et al.* A dynamic global vegetation model for studies of the coupled atmosphere-biosphere system. *Global Biogeochemical Cycles* **19**, doi:Gb1015/10.1029/2003gb002199 (2005).
125 Piao, S. *et al.* Changes in climate and land use have a larger direct impact than rising CO2 on global river runoff trends. *Proceedings of the National Academy of Sciences of the United States of America* **104**, 15242-15247, doi:10.1073/pnas.0707213104 (2007).
126 Chang, J. F. *et al.* Incorporating grassland management in ORCHIDEE: model description and evaluation at 11 eddy-covariance sites in Europe. *Geoscientific Model Development* **6**, 2165-2181, doi:10.5194/gmd-6-2165-2013 (2013).
127 Graux, A. I. *et al.* Development of the Pasture Simulation Model for assessing livestock production under climate change. *Agriculture Ecosystems & Environment* **144**, 69-91, doi:10.1016/j.agee.2011.07.001 (2011).

128 Riedo, M., Grub, A., Rosset, M. & Fuhrer, J. A pasture simulation model for dry matter production, and fluxes of carbon, nitrogen, water and energy. *Ecological Modelling* **105**, 141-183, doi:10.1016/s0304-3800(97)00110-5 (1998).
129 Vuichard, N., Ciais, P., Viovy, N., Calanca, P. & Soussana, J.-F. Estimating the greenhouse gas fluxes of European grasslands with a process-based model: 2. Simulations at the continental level. *Global Biogeochemical Cycles* **21**, doi:Gb1005/10.1029/2005gb002612 (2007).
130 Vuichard, N. *et al.* Estimating the greenhouse gas fluxes of European grasslands with a process-based model: 1. Model evaluation from in situ measurements. *Global Biogeochemical Cycles* **21**, doi:Gb1004/10.1029/2005gb002611 (2007).
131 Chang, J. *et al.* Modeled Changes in Potential Grassland Productivity and in Grass-Fed Ruminant Livestock Density in Europe over 1961–2010. *PloS one* **10**, e0127554.(2015).
132 Chang, J. *et al.* Future productivity and phenology changes in European grasslands for different warming levels: implications for grassland management and carbon balance. *Carbon Balance and Management* **12**, 11 (2017).
133 Chang, J. *et al.* Combining livestock production information in a process-based vegetation model to reconstruct the history of grassland management. *Biogeosciences* **13**, 3757-3776 (2016).
134 Harris, I., Jones, P. D., Osborn, T. J. & Lister, D. H. Updated high‐resolution grids of monthly climatic observations–the CRU TS3. 10 Dataset. *International Journal of Climatology* **34**, 623-642 (2014).
135 Wei, Y. *et al.* The North American Carbon Program Multi-scale Synthesis and Terrestrial Model Intercomparison Project-Part 2: Environmental driver data. *Geoscientific Model Development* **7**, 2875-2893 (2014).
136 Keeling, C. D. & Whorf, T. P. Atmospheric CO2 records from sites in the SIO air sampling network. *Trends: a compendium of data on global change*, 16-26 (2005).
137 Bontemps, S. *et al.* Consistent global land cover maps for climate modelling communities: current achievements of the ESA's land cover CCI. *Proceedings of the ESA Living Planet Symposium.* 9-13 (2013).
138 Poulter, B. *et al.* Plant functional type mapping for earth system models. *Geoscientific Model Development* **4**, 993-1010 (2011).
139 Poulter, B. *et al.* Plant functional type classification for earth system models: results from the European Space Agency's Land Cover Climate Change Initiative. *Geoscientific Model Development* **8**, 2315-2328 (2015).
140 Hurtt, G. *et al.* Harmonization of land-use scenarios for the period 1500–2100: 600 years of global gridded annual land-use transitions, wood harvest, and resulting secondary lands. *Climatic Change* **109**, 117-161 (2011).
141 Klein Goldewijk, K., Beusen, A., Doelman, J. & Stehfest, E. Anthropogenic land use estimates for the Holocene–HYDE 3.2. *Earth System Science Data* **9**, 927 (2017).
142 Eyring, V. *et al.* Overview of IGAC/SPARC Chemistry-Climate Model Initiative (CCMI) community simulations in support of upcoming ozone and climate assessments. *Sparc Newsletter* **40**, 48-66 (2013).
143 Reynolds, C. A., Jackson, T. J. & Rawls, W. J. Estimating soil water-holding capacities by linking the Food and Agriculture Organization Soil map of the world with global pedon databases and continuous pedotransfer functions. *Water Resources Research* **36**, 3653-3662, doi:10.1029/2000wr900130 (2000).
144 Hoekstra, J. M. *et al.* The Atlas of Global Conservation: Changes, Challenges, and Opportunities to Make a Difference, University of California Press, Berkeley (2010), 272 pp., ISBN: 978-0-520-26256-0. Urban & Fischer (2010).

145 IPCC. *2006 IPCC guidelines for national greenhouse gas inventories*. (Institute for Global Environmental Strategies Hayama, Japan, 2006).

146 Eurostat. https://ec.europa.eu/eurostat/statistics-explained/index.php/Glossary:Livestock_unit_%28LSU%29. (2013).

147 Robinson, T. P. *et al. Global livestock production systems*. (FAO and ILRI, 2011).

148 Mitchell, B. R. *International Historical Statistics: The Americas*. (MacMillan Publishers Limited, 1993).

149 Mitchell, B. *International historical statistics: Europe 1750-1993*. (Springer, 1998).

150 Mitchell, B. International historical statistics: Africa, Asia and Oceania, 1750-1993. *London: Macmillan Reference/New York: Stockton Press.* **1993**, 1998 (1998).

151 Kreileman, E., Van Woerden, J. & Bakkes, J. RIVM Environmental Research. *CIM Rep. M025* **98** (1998).

152 Leip, A., Weiss, F., Lesschen, J. P. & Westhoek, H. The nitrogen footprint of food products in the European Union. *Journal of Agricultural Science* **152**, S20-S33, doi:10.1017/s0021859613000786 (2014).

153 Leip, A. *et al.* Linking an economic model for European agriculture with a mechanistic model to estimate nitrogen and carbon losses from arable soils in Europe. *Biogeosciences* **5**, 73-94 (2008).

154 Leip, A., Britz, W., Weiss, F. & de Vries, W. Farm, land, and soil nitrogen budgets for agriculture in Europe calculated with CAPRI. *Environmental Pollution* **159**, 3243-3253, doi:10.1016/j.envpol.2011.01.040 (2011).

155 FAO/IFA/IFDC. Fertilizer use by crop, Fourth Edition, Rome. (1999).

156 Association, I. F. I. *Global estimates of gaseous emissions of NH3, NO and N2O from agricultural land*. (Food and Agriculture Organization of the United Nations (FAO), 2001).

157 International Fertilizer Industry Association. Nitrogen-Phosphate-Potash, IFADATA statistics from 1973/74–1973 to 1997/98–1997 including separately world fertilizer consumption statistics. Paris. (1999).

158 Bouwman, A., Boumans, L. & Batjes, N. Modeling global annual N2O and NO emissions from fertilized fields. *Global Biogeochemical Cycles* **16**, 28-21-28-29 (2002).

159 Bouwman, A., Boumans, L. & Batjes, N. Estimation of global NH3 volatilization loss from synthetic fertilizers and animal manure applied to arable lands and grasslands. *Global Biogeochemical Cycles* **16**, 8-1-8-14 (2002).

160 FAO/IFA/IFDC/IPI/PPI. Fertilizer use by crop, Fifth Edition, Rome. (2002).

161 Bouwman, A. F., Van der Hoek, K. W., Eickhout, B. & Soenario, I. Exploring changes in world ruminant production systems. *Agricultural Systems* **84**, 121-153, doi:10.1016/j.agsy.2004.05.006 (2005).

162 Damuth, J. Population density and body size in mammals. *Nature* **290**, 699 (1981).

163 Smith, F. A., Elliott, S. M. & Lyons, S. K. Methane emissions from extinct megafauna. *Nature Geoscience* **3**, 374-375 (2010).

164 Zhu, D. *et al.* The large mean body size of mammalian herbivores explains the productivity paradox during the Last Glacial Maximum. *Nature Ecology & Evolution* **2**, 640-649(2018).

165 Ripple, W. J. *et al.* Collapse of the world's largest herbivores. *Science Advances* **1**, e1400103 (2015).

166 Flechard, C. R. *et al.* Effects of climate and management intensity on nitrous oxide emissions in grassland systems across Europe. *Agriculture Ecosystems & Environment* **121**, 135-152, doi:10.1016/j.agee.2006.12.024 (2007).

167 Peel, M. C., Finlayson, B. L. & McMahon, T. A. Updated world map of the Koppen-Geiger climate classification. *Hydrology and Earth System Sciences* **11**, 1633-1644 (2007).
168 Tans, P. P. & Keeling, R. Trends in Atmospheric Carbon Dioxide – Mauna Loa CO2 annual mean data, available at: http://www.esrl.noaa.gov/gmd/ccgg/trends/, last access: August 2017. . (2017).
169 Prinn, R. G. *et al.* History of chemically and radiatively important atmospheric gases from the Advanced Global Atmospheric Gases Experiment (AGAGE). *Earth System Science Data* **10**, 985-1018 (2018).
170 Ciais, P. *et al.* Attributing the increase in atmospheric CO2 to emitters and absorbers. *Nature Climate Change* **3**, 926 (2013).
171 Gasser, T., Guivarch, C., Tachiiri, K., Jones, C. & Ciais, P. Negative emissions physically needed to keep global warming below 2 C. *Nature Communications* **6**, 7958 (2015).
172 Li, B. G. *et al.* The contribution of China's emissions to global climate forcing. *Nature* **531**, 357-+, doi:10.1038/nature17165 (2016).
173 Gasser, T. *et al.* Historical CO2 emissions from land use and land cover change and their uncertainty. *Biogeosciences* **17**, 4075-4101, doi:10.5194/bg-17-4075-2020 (2020).
174 Bright, R. M. & Kvalevag, M. M. Technical Note: Evaluating a simple parameterization of radiative shortwave forcing from surface albedo change. *Atmospheric Chemistry and Physics* **13**, 11169-11174, doi:10.5194/acp-13-11169-2013 (2013).
175 Lenton, T. M. & Vaughan, N. E. The radiative forcing potential of different climate geoengineering options. *Atmospheric Chemistry and Physics* **9**, 5539-5561 (2009).
176 GEWEX. Surface Radiation Budget (SRB) Release 3.0, available at: https://eosweb.larc.nasa.gov/project/srb/srb_rel3.0_sw_monthly_local_nc_table (last access: June 2013). . (2010).
177 CERES. Energy Balanced And Filled (EBAF) Surface Product, available at: https://ceres.larc.nasa.gov/data/, last access: November 2015. (2015).
178 MERRA. Monthly IAU 2d surface and TOA radiation fluxes, available at: https://disc.gsfc.nasa.gov/datasets/MATMNXRAD_5.2.0/summary, last access: October 2015. (2015).
179 Muller, J.-P. *et al.* The ESA GlobAlbedo Project for mapping the Earth's land surface albedo for 15 Years from European Sensors. *Geophysical Research Abstracts.* 10969 (2012)
180 Schaaf, C. & Wang, Z. MCD43C3 MODIS/Terra+Aqua BRDF/Albedo Albedo Daily L3 Global 0.05Deg CMG V006. 2015, distributed by NASA EOSDIS Land Processes DAAC, https://doi.org/10.5067/MODIS/MCD43C3.006. (2015).
181 Channan, S., Collins, K. & Emanuel, W. Global Mosaics of the Standard MODIS Land Cover Type Data. University of Maryland and the Pacific Northwest National Laboratory, College Park, Maryland, USA. (2014).
182 Bartholomé, E. & Belward, A. GLC2000: a new approach to global land cover mapping from Earth observation data. *International Journal of Remote Sensing* **26**, 1959-1977 (2005).
183 He, T., Liang, S. L. & Song, D. X. Analysis of global land surface albedo climatology and spatial-temporal variation during 1981-2010 from multiple satellite products. *Journal of Geophysical Research-Atmospheres* **119**, doi:10.1002/2014jd021667 (2014).
184 Zhu, Z. *et al.* Global data sets of vegetation leaf area index (LAI) 3g and Fraction of Photosynthetically Active Radiation (FPAR) 3g derived from Global Inventory Modeling and Mapping Studies (GIMMS) Normalized Difference Vegetation Index (NDVI3g) for the period 1981 to 2011. *Remote Sens-Basel* **5**, 927-948 (2013).

185 Scurlock, J. M. O. & Hall, D. O. The global carbon sink: a grassland perspective. *Global Change Biology* **4**, 229-233, doi:10.1046/j.1365-2486.1998.00151.x (1998).

186 Hanegraaf, M., Hoffland, E., Kuikman, P. & Brussaard, L. Trends in soil organic matter contents in Dutch grasslands and maize fields on sandy soils. *European Journal of Soil Science* **60**, 213-222 (2009).

187 Eva, H. D. *et al.* A land cover map of South America. *Global Change Biology* **10**, 731-744 (2004).

188 Klein Goldewijk, K., Beusen, A., Van Drecht, G. & De Vos, M. The HYDE 3.1 spatially explicit database of human ‐ induced global land ‐ use change over the past 12,000 years. *Global Ecology and Biogeography* **20**, 73-86 (2011).
